# Organic photoredox-catalyzed unimolecular PCET of benzylic alcohols†

**DOI:** 10.1039/d4sc07048h

**Published:** 2025-01-08

**Authors:** Tomotoki Matsuo, Masaki Sano, Yuto Sumida, Hirohisa Ohmiya

**Affiliations:** a Institute for Chemical Research, Kyoto University Gokasho Uji Kyoto 611-0011 Japan ohmiya@scl.kyoto-u.ac.jp; b Division of Pharmaceutical Sciences, Graduate School of Medical Sciences, Kanazawa University Kakuma-machi Kanazawa 920-1192 Japan; c Chemical Bioscience Team, Laboratory for Biomaterials and Bioengineering, Institute of Integrated Research, Institute of Science Tokyo Tokyo 101-0062 Japan sumida.yuto@tmd.ac.jp

## Abstract

Proton-coupled electron transfer (PCET) is a crucial chemical process involving the simultaneous or sequential transfer of protons and electrons, playing a vital role in biological processes and energy conversion technologies. This study investigates the use of an organic photoredox catalyst to facilitate a unimolecular PCET process for the generation of alkyl radicals from benzylic alcohols, with a particular focus on alcohols containing electron-rich arene units. By employing a benzophenone derivative as the catalyst, the reaction proceeds efficiently under photoirradiation, achieving significant yields without the need for a Brønsted base. The findings highlight the potential of this unimolecular PCET mechanism to streamline radical generation in organic synthesis, offering a more efficient and flexible alternative to conventional methods.

## Abstract 2

Proton-coupled electron transfer (PCET) is a crucial chemical process involving the simultaneous or sequential transfer of protons and electrons, playing a vital role in biological processes and energy conversion technologies.

## Graphical abstract

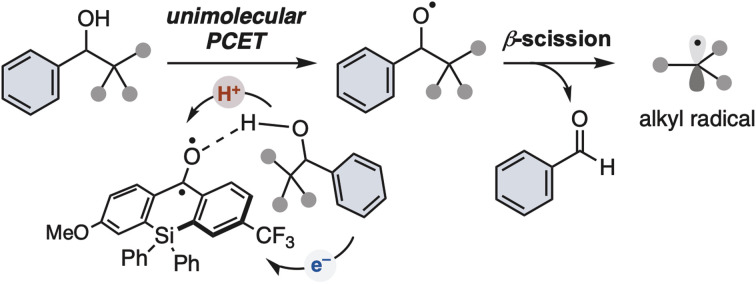

Proton-coupled electron transfer (PCET) is a fundamental chemical process in which protons and electrons are transferred simultaneously or sequentially (Fig. 1A).^1,2^ This process plays a significant role as an elementary step in biological processes (*e.g.*, photosynthesis and cellular respiration) and energy conversion technologies (*e.g.*, fuel cells and solar cells).^3^ The PCET reaction combines two basic steps, electron transfer (ET) and proton transfer (PT), which proceed either concertedly or stepwise. The reaction mechanism of PCET is strongly influenced by oxidants and bases. For example, the oxidative PT/ET mechanism predominates when a moderately strong base is used, and an equilibrium concentration of the substrate's conjugate base is present in the reaction solution. The ET/PT-type mechanism also prevails when a moderately strong oxidant is used to oxidise the substrate directly, promoting the activation of more electron-rich substrates. The use of the PCET mechanism for bond cleavage in organic synthesis has not been extensively explored. In the past decade, it has been demonstrated that free alkyl radical intermediates can be generated under mild conditions and directly from readily available starting materials based on the PCET mechanism, with notable contributions by the Knowles group (Fig. 1B-I).^4^ They described that photoredox-catalysed multi-site PCET (MS-PCET) with mild Brønsted bases in a cooperative manner allows the direct generation of energetic intermediates, such as alkoxy radicals from alcohols, despite their high BDFE (*ca.* 105 kcal mol^−1^).^5^ Conventional methods for alkoxy radical generation require the preparation of pre-functionalised radical precursors with readily activatable O–X bonds.^6^ These designed precursors can efficiently undergo thermal/photo/radical-induced bond cleavage to generate alkoxy radicals, which serve as synthetically useful intermediates, commonly producing C-centred radicals along with aldehyde or ketone *via* β-scission. Recent advances in alkoxy radical generation have been achieved in conjunction with transition-metal-based photoredox-catalysed, photoinduced PCET and LMCT.^7^ More recently, organic photoredox catalysis based on acridinium salt (Fukuzumi catalyst) has served as a strong single-electron oxidant, though examples are limited.^8^ Alkyl radicals generated from alcohols have been applied to C–C bond formations, such as Giese addition, Minisci reaction, and Ni-catalysed cross-coupling (Fig. 1B-II).^8^ Overall, oxidative MS-PCET enables direct activation of C–H or O/N–H bonds *via* electron and proton transfers to two distinct acceptors. Recent developments typically employ a photoredox catalyst as the electron-transfer agent and a Brønsted base as the proton-transfer reagent separately.^4,6–8^ By physically partitioning the photoredox catalyst and proton-transfer reagent, the MS-PCET mechanism covers a broader thermodynamic range and alters chemoselectivity compared to conventional hydrogen atom transfer (HAT) mechanisms, offering more flexibility in reaction conditions. However, this approach is entropically unfavourable and often results in reduced reaction efficiency due to multiple intermolecular interactions. In this context, establishing a unimolecular organic photocatalytic PCET process not only leads to more efficient alkoxy radical generation but also eliminates the previously essential Brønsted base.

**Fig. 1:**
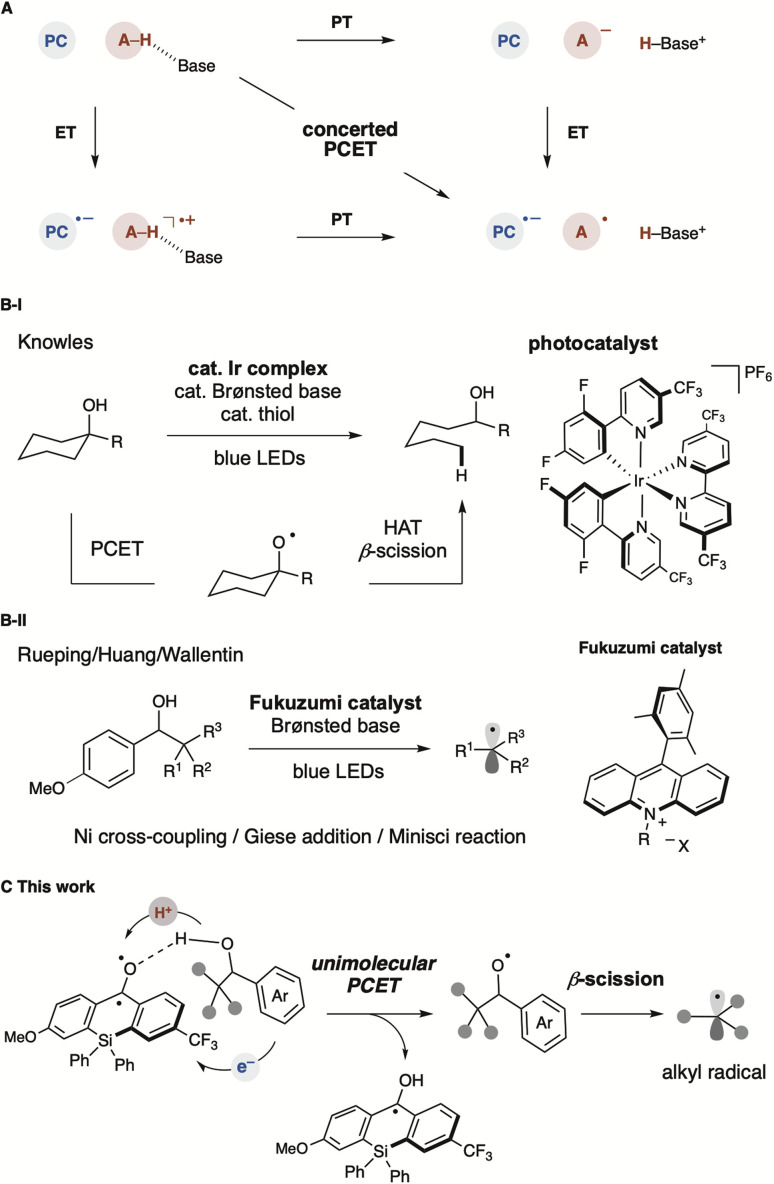
Proton-coupled electron transfer process (A) schematic model for PCET processes. Flavin-promoted PCET mechanism in biological processes. (B) Ir photoredox-catalysed PCET enabling generation of alkoxy radical. Application of alkyl radical *via* β-scission to organic synthesis. (C) Organic photoredox-catalysed unimolecular PCET enabling alkyl radical generation (this work).

We envisioned that the benzophenone catalyst could facilitate the generation of alkyl radicals from alcohols containing electron-rich arene units *via* β-scission through a Brønsted base-free PCET process (Fig. 1C). Benzophenone and its derivatives exhibit closely matched energies in the S1 and T1 states, facilitating almost quantitative and expeditious intersystem crossing (*φ*_ISC_ = 1.0, *k*_ISC_ = 1 × 10^11^ s^−1^).^9^ This characteristic enables these molecules to rapidly form excited triplet states upon photoirradiation, leading to oxidation and reduction reactions *via* electron or energy transfer, as well as HAT processes *via* oxygen radicals. In contrast, the unprecedented unimolecular PCET described in this study is achieved by forming a hydrogen bonding network between the carbonyl group of benzophenone and the alcohol, followed by single-electron oxidation in the excited state.

To verify our hypothesis, we initially prepared the benzylic alcohol 1a as a model substrate having an electron-rich arene unit, which contributes to efficient reductive quenching of the excited photocatalyst. We evaluated catalytic activity by the production efficiency of aromatic aldehyde 2a with a variety of benzophenone derivatives. Delightfully, with a catalytic amount of benzophenone (PC1) (*E*_red_ = +1.53 V *vs.* SCE in MeCN)^10^ and substoichiometric 2,4,6-triisopropylbenzenethiol (TRIP thiol), the reaction of alcohol 1a provided 2a in 61% yield under photoirradiation (Table 1, entry 1). Thioxanthone (PC2) (*E*_red_ = +1.43 V *vs.* SCE in MeCN)^10^ showed higher activity as a photocatalyst, and the product was obtained in high yield even with 1 mol% of the catalyst (entry 2). While anthraquinone (PC3), working as a strong oxidant (*E*_red_ = +1.98 V *vs.* SCE in MeCN),^11^ afforded the product 2a in 90% yield, low loading of photocatalyst reduced reaction efficiency (entry 3). We found that silicon-bridged benzophenone derivative PC4 (*E*_red_ = +1.43 V *vs.* SCE in MeCN)^12^ revealed comparable activity with PC2 and PC3 (entry 4). Consequently, we evaluated disubstituted benzophenone catalyst PC5, bearing an electron-withdrawing and an electron-donating group on each arene, reported by the Molander group 
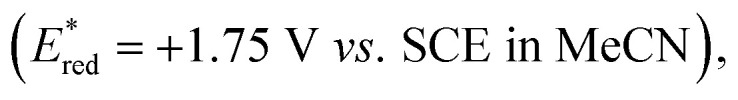
^13^ and higher yield was observed compared to unsubstituted benzophenone PC1 (entry 5). This result can be attributed to the captodative effect, which stabilises both the triplet ketyl radical and the protonated radical intermediate *via* PCET. Consequently, we designed and synthesised the push–pull type silicon-bridged benzophenone PC6, which exhibited the most effective catalytic activity for PCET-promoted β-scission (entry 6). Several conventional photoredox catalysts used in recent PCET chemistry were also evaluated without Brønsted bases. In the presence of [Ir(dFCF_3_ppy)_2_(5,5′-dCF_3_bpy)]PF_6_ (PC7) (*E*_red_ = +1.68 V *vs.* SCE in MeCN),^14^ no reaction of 1a was observed. Despite the advantage of its high reduction potential in the excited state, only a low yield of aldehyde 2a was obtained when using [(*t*Bu)_2_MesAcr]BF_4_ (PC8) (*E*_red_ = +2.15 V *vs.* SCE in MeCN),^15^ a photocatalyst effective for PCET in the presence of Brønsted base (entry 8). The reaction using 4CzIPN (PC9) 
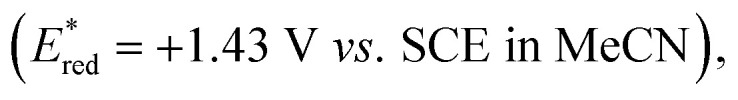
^16^ which has a redox potential comparable to PC6, provided product 2a in a 17% yield (entry 9). We also investigated the effects of thiols and solvents. Arylthiol had no significant influence on yield (entry 10), whereas aliphatic thiol or the absence of thiol as an additive significantly hindered product formation (entries 11 and 12). Solvent changes had little impact on the reaction outcome (entry 13, see ESI†). It was confirmed that no reaction occurred without photocatalyst or photoirradiation (entries 14 and 15). Considering the mechanism of photoredox reactions, the redox potentials of photocatalysts and substrates are critical factors for catalytic activity. Therefore, we calculated the redox potentials for the reductive quenching of benzophenone derivatives and other photocatalysts, including PC7–PC9, to optimise reaction conditions (Table 1). However, no clear correlation with yields was observed (Fig. 2A). Absorption wavelength, molar absorption coefficient, and excitation lifetime also affect photoredox catalysis efficiency. Among the photocatalysts that gave high yields, the push–pull type benzophenone catalysts PC5 and PC6 displayed red-shifted absorptions compared to the unsubstituted benzophenones PC1 and PC4. Thioxanthone (PC2) and anthraquinone (PC3), which also showed high catalytic activity, had relatively longer absorption wavelengths, extending to around the reaction wavelength of 390 nm, although their molar absorption coefficients were low (Fig. 2B). These results suggest that wavelength may influence catalytic efficiency. Subsequently, we evaluated the triplet state and redox potential of PC6 using computational calculations, UV/vis spectroscopy, fluorescence, and cyclic voltammetry (Fig. 2B and D). The Kohn–Sham orbital, calculated at the UM062X/6-311++G(d,p) level, showed the distribution of the LUMO (Fig. 2C). The excited state of benzophenone acts as a biradical, with the C–O bond lengthening from 1.22 Å to 1.33 Å compared to the C=O bond in the X-ray structure^17^ (Fig. 2E).

**Table 1:** Screening of reaction conditions

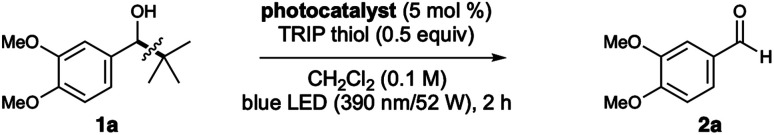		
Entry	Photocatalyst^a^	Yield^b^ (%) of 2a
1	Benzophenone (PC1)	61
2	Thioxanthone (PC2)	83(83)^c^
3	Anthraquinone (PC3)	90(65)
4	PC4	87(59)^c^
5	PC5	77
6	PC6	93^d^(86)^c^
7	[Ir(dFCF_3_ppy)_2_(dtbpy)]PF_6_ (PC7)	0^c^
8	[(*t*Bu)_2_MesAcr]BF_4_ (PC8)	6^c^
9	4CzIPN (PC9)	17^c^
10	C_6_H_5_SH instead of TRIP thiol	84^e^
11	*n*-Dodecanethiol instead of TRIP thiol	28
12	W/o thiol	12^e^
13	MeCN instead of CH_2_Cl_2_	80^e^
14	W/o photocatalyst	0
15	Under dark	0^e^
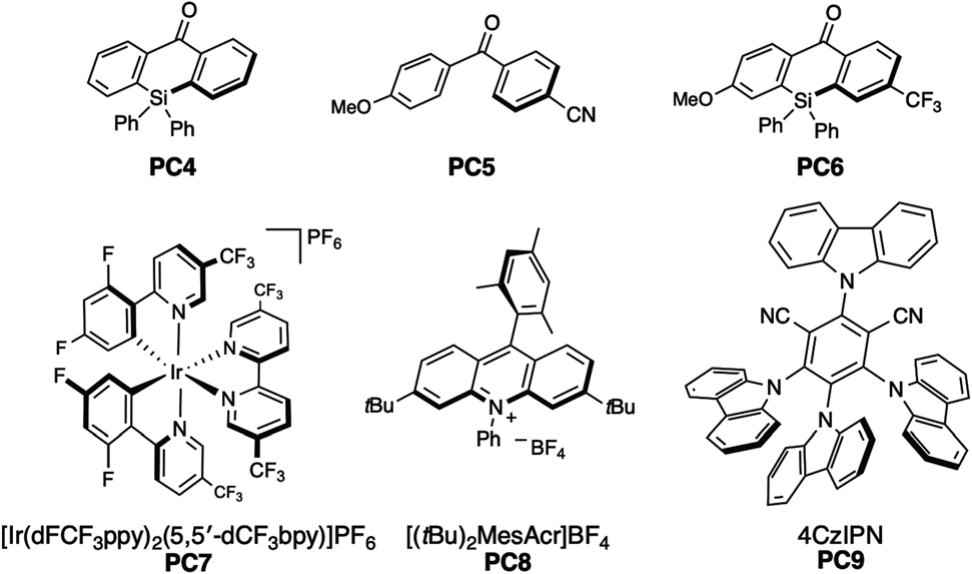		

a Reaction was carried out with 1a (0.1 mmol), photocatalyst (5 μmol), and TRIP thiol (0.05 mmol) in CH_2_Cl_2_ (1 mL) under 390 nm blue LED (Kessil lamp) irradiation at ambient temperature for 2 h.

b ^1^H NMR yield.

c 1 mol% of photocatalyst was used.

d Isolated yield.

e 5 mol% of PC6 was used as photocatalyst.

**Fig. 2:**
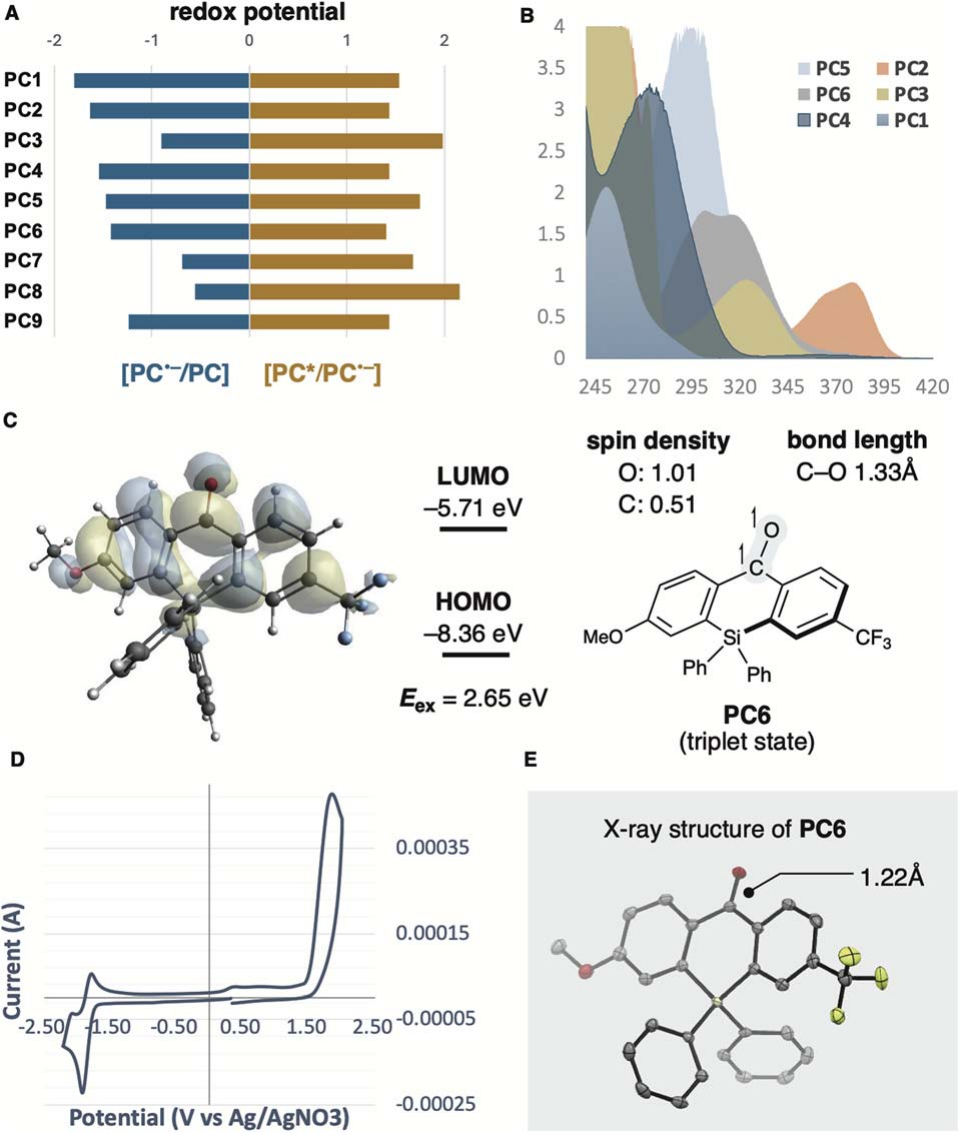
(A) Redox potentials for reductive quenching of PC1–9. (B) UV/vis spectra of PC1–PC6 (20 μM in MeCN). (C) Triplet state energies and structural property of PC6 calculated by DFT at the UM062X/6-311++G(dp) level. (D) Cyclic voltammogram PC6 (100 μM in MeCN), electrolyte: *n*Bu_4_NClO_4_ RE: Ag/AgNO_3_, WE: glassy carbon, CE: Pt wire. (E) ORTEP diagram of PC6 (CCDC 2352222).

The PC6-catalysed β-scission of electron-rich benzylic alcohols *via* unimolecular photo-PCET demonstrated excellent functional group compatibility (Fig. 3A). Reactions were conducted under optimised conditions: using dichloromethane as solvent, exposure to blue LED light at 390 nm for two hours, 5 mol% PC6 as catalyst, and a substoichiometric amount of TRIP thiol as a co-catalyst. The effects of each additive (A1–A26) were assessed in terms of aldehyde 2a formation and additive recovery.^18^ Fortunately, almost all additives had no significant effect on the target transformation, with quantitative recovery of additives. Despite the HAT ability of benzophenone-type catalysts, additives containing benzylic protons did not inhibit the β-scission process, and aldehyde 2a was obtained in high yield with quantitative recovery of A1 or A2. Additives A3–9, containing reactive functional groups or protic compounds, such as alcohols and acids (A10–12), were also ineffective.

**Fig. 3:**
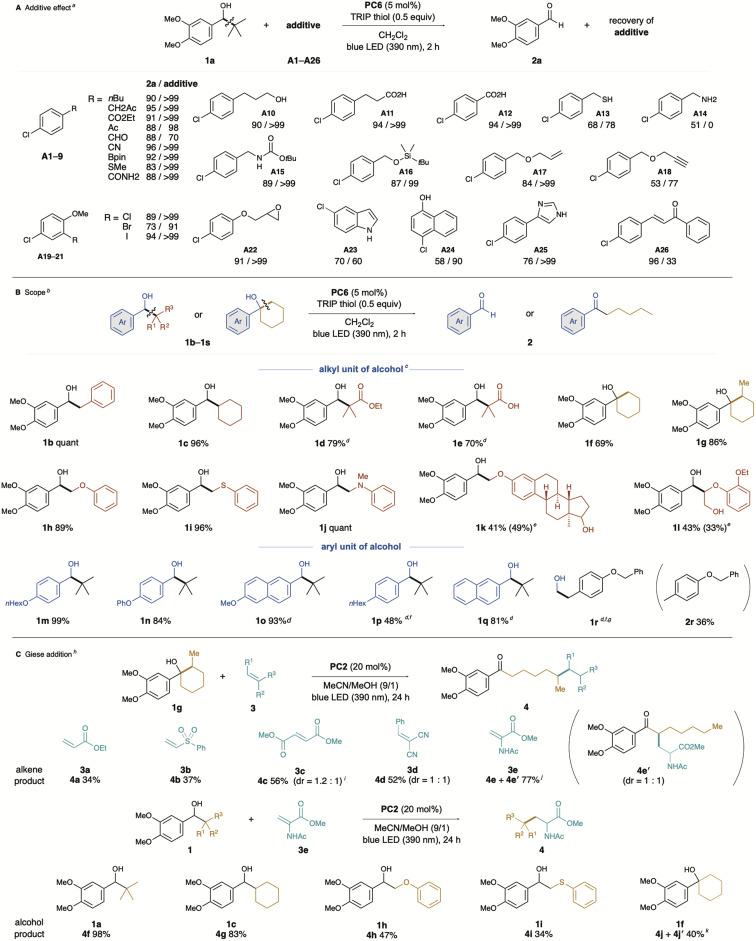
(A) Additive effect on catalytic photo-PCET. (a) 1 equivalent of additive was added. (B) Substrate scope of alcohols. (b) *Cis*-isomerisation was observed. (c) Reaction was carried out with 1 (0.2 mmol), PC6 (0.01 mmol), and TRIP thiol (0.1 mmol) in CH_2_Cl_2_ (2 mL) under 390 nm blue LED (Kessil lamp) irradiation at ambient temperature for 2 h. (d) Anthraquinone was used as a photocatalyst. (e) Yield of HAT product in parenthesis. (f) 10 mol% of photocatalyst was used. (g) Reaction was stirred for 6 h. (C) Giese addition (h) reaction was carried out with 1 (0.3 mmol), PC2 (0.04 mmol), and alkene 3 (0.2 mmol) in MeCN/MeOH (9/1, 2 mL) under 390 nm blue LED (Kessil lamp) irradiation at ambient temperature for 24 h. (i) Reaction was carried out with 1g (0.2 mmol), PC2 (0.04 mmol), and alkene 3 (0.4 mmol) in MeCN (2 mL) under 390 nm blue LED (Kessil lamp) irradiation at ambient temperature for 24 h. (j) 4e : 4e′ = 1.8 : 1, dr of 4e = 1 : 1. (k) 4j : 4j′ = 1 : 1.9, dr of 4j′ = 1 : 1.3.

Recent reports of catalytic photo-PCET of alcohols or amides usually require a base for concerted deprotonation, suggesting that acidic substrates may not be compatible. However, some additives showed moderate to low efficiency in the PCET process, affecting either conversion rates or additive recovery.

For instance, additive A13 (thiol) resulted in a 68% conversion rate and 77% recovery of thiol, while A14 (benzylamine) had a detrimental effect, with a 51% conversion and no recovery. Additives A18, A24, and A25 affected alcohol 1a conversion, while A23 and A26 resulted in low additive recovery, likely due to undesired alkyl radical addition pathways. Although A25 could act as a hydrogen atom transfer (HAT) donor or a mild base, comparison with the calculated BDFEs for thiol (81.6 and 67.5 kcal mol^−1^) suggests no significant impact on the reaction (see ESI†).

Next, we investigated the substrate scope (Fig. 3B), using PC6 as an organic photocatalyst to initiate unimolecular PCET, leading to the formation of aldehydes or ketones *via* β-scission of the hydroxyl group. The substrate scope included various alcohols with different substituents, demonstrating the versatility of the reaction. Initially, we examined alkyl units generating the alkyl radical from substrates with electron-rich arenes. In addition to 1a, which has *t*Bu groups, substrates with benzyl and cyclohexyl groups (1b and 1d) gave 3,4-dimethoxybenzaldehyde (2a) efficiently. β-Hydroxyesters 1d and 1e, easily prepared by the Aldol reaction, also yielded 2a in high yields. This suggests that the C–C bond formed in the two-electron process can behave as an alkyl radical *via* β-scission. Additionally, 1e, with an intramolecular carboxylic acid, was challenging for conventional photocatalyst-base cooperative PCET but performed well here. Ring-opening occurred in the case of cyclic alkyl alcohols, such as 1f and 1g, yielding the corresponding ketones 2f and 2g. Similarly, alcohols with a heteroatom substituted at the β-position, such as 1h, 1i, and 1j, were easily synthesised by nucleophilic substitution of the corresponding α-bromoketone with a nucleophile such as alcohol, followed by reduction of the carbonyl group. C–C bond cleavage proceeded efficiently with these substrates, and 2a was obtained in high yield. In the estradiol derivative 1k,^8*d*^ while PCET proceeded only with the electron-rich arene-substituted alcohol, the other alcohol unit remained intact, yielding aldehyde 2a and a product in which the phenolic hydroxyl group of estradiol was formally methylated. Furthermore, photo-PCET has recently gained attention as a powerful tool in lignin degradation,^19^ a representative biomass. Our catalytic photo-PCET system similarly worked with the lignin model molecule 1l, leading to degradation that afforded the corresponding aldehydes 2a and phenols. Next, we examined the reaction efficiency by varying the aryl unit of the substrate.

The monoalkoxy-substituted substrate 1m achieved quantitative conversion, showcasing the reaction's applicability with different structural motifs. The phenoxy group-substituted substrate 1n yielded the corresponding aldehyde in 84%, further illustrating the effectiveness of the method. Using anthraquinone (PC3) as a photocatalyst, which acts as a stronger oxidant, alcohols with less activated arenes, such as naphthyl and alkylphenyl-substituted alcohols (1o, 1p, and 1q), were successfully converted into the corresponding aldehydes. Pleasingly, non-benzylic alcohol 1r was also applicable to our catalytic system, yielding 2r in moderate yield *via* C–C bond cleavage, with formaldehyde also formed. We then explored the potential of this method for generating alkyl radicals from aliphatic alcohols (Fig. 3C). After re-screening the photocatalytic conditions for Giese addition, we found that PC2, thioxanthone, provided the best results. We attribute this to the distinct mechanism of the Giese addition, which slightly alters the required photocatalytic performance. The β-alkylation of acrylate 3a and vinylsulfone 3b proceeded, albeit with low yields of the corresponding products 4a and 4b. The reaction of malonate 3c and benzylidene malononitrile 3d, both more electron-deficient alkenes, afforded moderate yields (4c and 4d). Fortunately, high efficiency in alkyl radical addition was observed with dehydroalanine derivative 3e, suggesting that this protocol could be applied to protein modification.^20^ Whereas the reaction using 1g as an alkyl radical source resulted in a regioisomeric mixture (4e + 4e′), the regioisomer 4e′ likely formed *via* a 1,5-HAT process after PCET-promoted ring-opening β-scission of 3e. Based on the bond dissociation energy (BDE) comparison between a typical C–H bond (100 kcal mol^−1^)^21^ and an α-carbonyl C–H bond (92 kcal mol^−1^),^22^ intramolecular HAT can readily occur, thus allowing the generated α-carbonyl radical to attack 3e, forming the regioisomer 4e′. We also explored the alcohol scope in Giese addition. Using alcohols 1a and 1c, which generate tertiary-butyl and cyclohexyl radicals, Giese addition produced high yields of the corresponding adducts (4f and 4g). This method also enabled the generation of α-oxy and α-thiomethyl radicals, applying them to radical additions that furnished the corresponding products 4h and 4i. The electron-rich arene-pendant cyclohexanol 1f gave results similar to 1g, producing a regioisomer 4j′*via* the intramolecular HAT process.

To gain insight into the reaction mechanism, we conducted several experiments and photophysical analyses. Minisci-type reaction was investigated using lepidine treated with trifluoroacetic acid as a substrate (Fig. 4A). When MnO_2_ was used as an oxidant in the presence of PC6, the corresponding alkylated product 5 was obtained in good yield, suggesting that our protocol can work even under acidic conditions and expanding its versatility. Using a stoichiometric amount of thioxanthone (PC2) in the presence of the radical trapping reagent TEMPO, the reaction afforded the *t*Bu radical adduct 7 in 55% yield. This suggests the formation of alkoxy radicals, followed by β-C–C bond cleavage to generate alkyl radicals (Fig. 4B). We also examined the interaction between the photocatalyst and the substrate in the reaction solution and the formation of charge transfer complexes (EDA complexes). The UV-vis spectrum of each component was measured, as well as that of the mixture (Fig. 4C). As a result, no data supporting the formation of an EDA complex were obtained. Substrate 1a exhibited no absorption in the visible light range as expected (Fig. 4C, red line), and no change in the absorption spectrum was observed for the mixture of PC2 and 1a. A photo-quenching experiment (Stern–Volmer plot) also provided mechanistic insight into the organic catalytic photo-PCET process. While no photo-quenching was observed for thioxanthone (PC2) with TRIP thiol (Fig. 4D, blue line), the Stern–Volmer plot for the substrate in the presence of PC2 showed significant photo-quenching of the photocatalyst (Fig. 4D, yellow line). Given the redox potential of PC6 [*E*_red_(PC*/PC˙^−^) = +1.43 V *vs.* SCE] and 1,2-dimethoxybenzene [*E*_ox_ = 1.42 V *vs.* SCE],^23^ SET oxidation of the substrate was feasible, but the SET efficiency for other benzylic alcohols with higher oxidation potentials, such as 1m–1s, could be significantly lower.

**Fig. 4:**
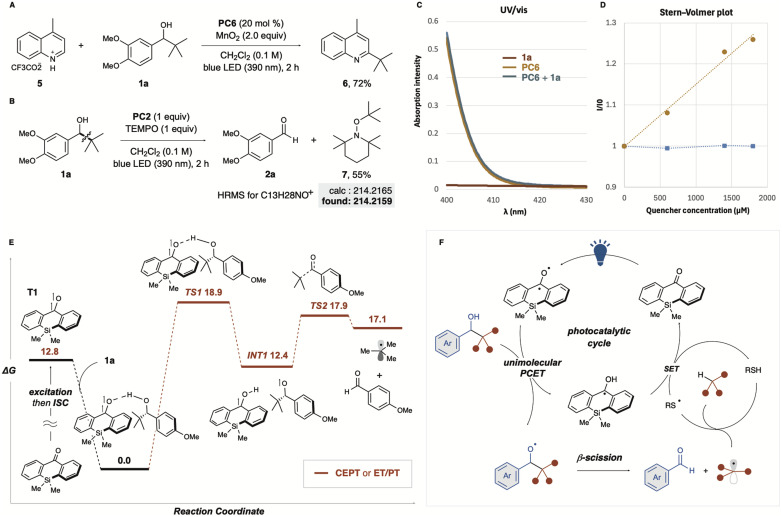
(A) Minisci-type reaction (B) radical trapping experiment (C) UV-vis absorption spectra of substrates. (D) Photo-quenching experiment. Yellow line (●): PC2 (200 μM) with alcohol 1a. Blue line (■): photocatalyst (40 μM) with TRIP thiol. (E) Energy profiles for the proposed pathway. Density functional theory calculations were carried out at the (U)M06-2X/6-311+G(d,p) level of theory, the unit of Gibbs free energies (Δ*G*) is kcal mol^−1^. (F) Plausible mechanism.

To further understand the reaction mechanism, we used computational methods to evaluate plausible pathways and energy profiles (Fig. 4E). The triplet state (T1) of the silicon-bridged benzophenone exhibited a higher energy level. Interestingly, the triplet state (T1) formed a highly stabilised complex through interaction with substrate 1a, which we defined as the starting point (0 kcal mol^−1^). The pathways leading to the formation of alkoxy and ketyl radicals (INT1) *via* transition state TS1 are proposed to involve either concerted electron/proton transfer (CEPT) or a stepwise electron transfer followed by proton transfer (ET/PT). However, distinguishing between these two pathways using DFT calculations proved challenging. TS2 represents the transition state for the formation of the *t*Bu radical *via* C–C bond cleavage of the generated alkoxy radical. Although the energy of TS2 was relatively high at 17.9 kcal mol^−1^, this value was reasonable. Based on experimental, analytical, and computational analyses, we propose the catalytic mechanism outlined in Fig. 4F. Unimolecular PCET of the excited photocatalyst with electron-rich benzyl alcohol derivatives generates an alkoxy radical, followed by β-C–C bond cleavage to form alkyl radicals and aldehydes. HAT with the cocatalytic thiol quenches the resulting alkyl radicals, forming thiyl radicals. Finally, ET/PT occurs between the ketyl radical and the thiyl radical, regenerating the catalyst. BDFE analysis provides additional support for the proposed PCET mechanism. The O–H bond dissociation free energy (BDFE) of the optimal catalyst PC6 (95.8 kcal mol^−1^) closely matches that of the substrate alcohol (95.5 kcal mol^−1^), enabling efficient hydrogen atom transfer. In contrast, thioxanthone, which has a higher BDFE (98.9 kcal mol^−1^), demonstrates slightly reduced activity, likely due to its less favourable energy match with the substrate. Furthermore, the thiol cocatalyst's low BDFE (67.5 kcal mol^−1^) highlights its role as HAT donor, facilitating the regeneration of the photocatalyst during the catalytic cycle. Considering that the Giese addition proceeds efficiently without the addition of thiol, thiol is not strictly required for the generation of alkyl radicals *via* the PCET process. Nonetheless, in the HAT system with thiol addition, it remains possible that the thiolate, generated through the reduction of the thiyl radical, acts as a mild base to facilitate the PCET process.

## Conclusions

In summary, we developed a Brønsted base-free, unimolecular PCET process catalysed by a benzophenone-type catalyst, which facilitates the generation of alkyl radicals from alcohols containing electron-rich arene units. The base-free PCET enables β-scission of substrates with protic functional groups, including carboxylic acids, probably due to the formation of a hydrogen-bonding network between the excited triplet state of benzophenone and the hydroxy group. This method presents a more efficient and flexible alternative to conventional approaches, with potential applications in C–C bond formations and other organic transformations.

## Data availability

The data supporting this article have been included as part of the ESI.†

## Author contributions

T. M., M. S., Y. S., and H. O. designed, performed and analysed the experiments. Y. S. and H. O. co-wrote the manuscript. All authors contributed to discussions.

## Conflicts of interest

There are no conflicts to declare.

## Supplementary Material

SC-016-D4SC07048H-s001

SC-016-D4SC07048H-s002

## References

[cit1] Weinberg D. R., Gagliardi C. J., Hull J. F., Murphy C. F., Kent C. A., Westlake B. C., Paul A., Ess D. H., McCafferty D. G., Meyer T. J. (2012). Chem. Rev..

[cit2] Murray P. R. D., Cox J. H., Chiappini N. D., Roos C. B., McLoughlin E. A., Hejna B. G., Nguyen S. T., Ripberger H. H., Ganley J. M., Tsui E. (2022). et al.. Chem. Rev..

[cit3] Mittra K., Green M. T. (2019). J. Am. Chem. Soc..

[cit4] Yayla H. G., Wang H., Tarantino K. T., Orbe H. S., Knowles R. R. (2016). J. Am. Chem. Soc..

[cit5] Blanksby S. J., Ellison G. B. (2003). Acc. Chem. Res..

[cit6] Chang L., An Q., Duan L., Feng K., Zuo Z. (2022). Chem. Rev..

[cit7] Guo J.-J., Hu A., Chen Y., Sun J., Tang H., Zuo Z. (2016). Angew. Chem., Int. Ed..

[cit8] Huang L., Ji T., Rueping M. (2020). J. Am. Chem. Soc..

[cit9] Romero N. A., Nicewicz D. A. (2016). Chem. Rev..

[cit10] Timpe H. J., Kronfeld K. P. (1989). J. Photochem. Photobiol., A.

[cit11] Bachman J. E., Curtiss L. A., Assary R. S. (2014). J. Phys. Chem. A.

[cit12] See ESI.†

[cit13] Campbell M. W., Yuan M., Polites V. C., Gutierrez O., Molander G. A. (2021). J. Am. Chem. Soc..

[cit14] Zhu Q., Gentry E. C., Knowles R. R. (2016). Angew. Chem., Int. Ed..

[cit15] Romero N. A., Margrey K. A., Tay N. E., Nicewicz D. A. (2015). Science.

[cit16] Uoyama H., Goushi K., Shizu K., Nomura H., Adachi C. (2012). Nature.

[cit17] Sumida Y. , CSD Communication, 2024, 10.5517/ccdc.csd.cc2jyp4b

[cit18] Saito N., Nawachi A., Kondo Y., Choi J., Morimoto H., Ohshima T. (2023). Bull. Chem. Soc. Jpn..

[cit19] Nguyen J. D., Matsuura B. S., Stephenson C. R. J. (2014). J. Am. Chem. Soc..

[cit20] Josephson B., Fehl C., Isenegger P. G., Nadal S., Wright T. H., Poh A. W. J., Bower B. J., Giltrap A. M., Chen L., Batchelor-McAuley C. (2020). et al.. Nature.

[cit21] Capaldo L., Ravelli D., Fagnoni M. (2022). Chem. Rev..

[cit22] John P. C. St., Guan Y., Kim Y., Kim S., Paton R. S. (2020). Nat. Commun..

[cit23] Luo P., Feinberg E. C., Guirado G., Farid S., Dinnocenzo J. P. (2014). J. Org. Chem..

