# Supplementary material for: Organic photoredox-catalyzed unimolecular PCET of benzylic alcohols
Source: Chem Sci. 2025 Jan 8;16(7):3150–6. doi: 10.1039/d4sc07048h (PMC11740339; doi:10.1039/d4sc07048h)
Supplement: SC-016-D4SC07048H-s001 [file SC-016-D4SC07048H-s001.pdf]

Supplementary Information (SI) for Chemical Science.
This journal is © The Royal Society of Chemistry 2025

Organic Photoredox-Catalyzed Unimolecular PCET of Benzylic Alcohols

Tomotoki Matsuo,[1] Masaki Sano,[2] Yuto Sumida,[3]* and Hirohisa Ohmiya[1]*

[1] Institute for Chemical Research, Kyoto University, Gokasho, Uji, Kyoto 611-0011, Japan
[2] Division of Pharmaceutical Sciences, Graduate School of Medical Sciences, Kanazawa University, Kakuma-machi, Kanazawa 920-1192, Japan
[3] Chemical Bioscience Team, Laboratory for Biomaterials and Bioengineering, Institute of Integrated Research, Institute of Science Tokyo, Tokyo, Japan.
*E-mail: Yuto Sumida : sumida.yuto@tmd.ac.jp
Hirohisa Ohmiya: ohmiya@scl.kyoto-u.ac.jp

Table of Contents

■ Supplementary Methods ■

1. Instrumentation and Chemicals
2. Synthesis of Photocatalysts **PC4**, **PC6**, **PC12** S3
3. Characterization Data for Photocatalysts S4
4. Characterization Data for Substrates S7
5. Optimization for *β*-Scission (HAT) S18
6. Procedure for *β*-Scission (HAT) S20
7. Characterization Data for Products of *β*-Scission (HAT) S20
8. Optimization for Giese Addition S25
9. Procedure for Giese Addition S26
10. Characterization Data for Products of Giese Addition S26
11. Minisci-type Reaction S32
12. Procedure for Additive Effect S33
13. UV/Vis Spectra of Photocatalysts S34
14. Fluorescence Spectra of Photocatalysts S39
15. Cyclic Voltammetry (CV) of Photocatalysts S44
16. Summary of Redox Properties of Organophotocatalysts S48
17. X-ray Diffraction Analysis for **PC6** S49
18. Procedure for Radical Trap Experiment S50
19. Stern–Volmer Experiment S50
20. EDA Complex S51
21. Computational Studies S52

■ Supplementary References ■ S77
■ NMR Spectra ■ S78

■ **Supplementary Methods** ■

1. Instrumentation and Chemicals

NMR spectra were recorded on a Bruker AVANCE NEO 400N spectrometer, operating at 400 MHz for ^{1}H NMR, 100.6 MHz for ^{13}C NMR, 376.5 MHz for ^{19}F NMR. Chloroform-d_1 ($CDCl_3$) containing 0.03% tetramethylsilane (TMS) (>99.8%D, Cambridge Isotope Laboratories, Inc., Cat. No. DLM-7) were used as solvents for NMR measurements at ambient temperature. Chemical shifts (δ) for ^{1}H NMR are given in parts per million (ppm) relative to TMS (δ 0.00 ppm in $CDCl_3$). Chemical shifts (δ) for ^{13}C NMR are given in ppm relative to $CDCl_3$ (δ 77.0 ppm). Chemical shifts were reported in δ ppm. The abbreviations s, d, t, q, quin, and m signify singlet, doublet, triplet, quartet, quintet and multiplet, respectively.

Mass spectra were obtained with a Bruker timsTOF mass spectrometer (ESI).

TLC analyses were performed on commercial glass plates of Merck Silica gel $60F_{254}$. Silica gel (Wakosil® 60, 64~210 µm) was used for column chromatography.

Biotage Selekt®, **Biotage Isolera One®** and **LaboACE LC-5060** (for Gel Permeation Chromatography) and were used for purification.

IR spectra were measured with an ATR Accessory (QATR-S) for the Shimadzu IRSpirit FT-IR Spectrometer.

Melting points were measured on a Stanford Research Systems MPA100.

UV-Vis absorption spectra were recorded on a Shimadzu UV-1900.

Fluorescence spectra were recorded on a Shimadzu RF-6000.

Cyclic voltammetry measurements were recorded with a Hokuto Denko HZ-7000 potentiostat.
Single-crystal X-ray diffraction data were collected on a Bruker Single Crystal CCD X-ray Diffractometer (SMART APEX II).

Reaction set-up and materials: Kessil PR-160 390 nm (max 52W) was used as a light source. TEKNOS MG9 was used as a fan.

All reactions were carried out under nitrogen atmosphere. Materials were obtained from commercial suppliers or prepared according to standard procedures unless otherwise noted. Before reactions, ethyl acrylate was passed through a short pad of Aluminium oxide 60. **PC1**, **PC3** and **PC10** were purchased from Tokyo Chemical Industry Co., stored under nitrogen, and used as received. **PC2** and **PC7** were purchased from BLD Pharmatech Ltd., stored under nitrogen, and used as received. **PC8** and **PC11** were purchased from Merk Sigma–Aldrich Japan Inc., stored under nitrogen, and used as received. **PC5** was prepared by the reported procedure.[1] TRIP thiol was prepared by the reported procedure.[2] 1-Dodecanthiol and Benzenethiol were purchased from Tokyo Chemical Industry Co., stored under nitrogen, and used as received. Triisopropylsilanethiol was purchased from Merk Sigma–Aldrich Japan Inc., stored under nitrogen, and used as received. Dichloromethane (super dehydrated), acetonitrile (super dehydrated), tetrahydrofuran (super dehydrated, stabilizer free), diethyl ether (super dehydrated) and methanol (super special grade) were purchased from Fujifilm Wako Pure Chemical Co., stored under nitrogen, and used as received.

2. Synthesis of Photocatalysts PC4, PC6, PC12

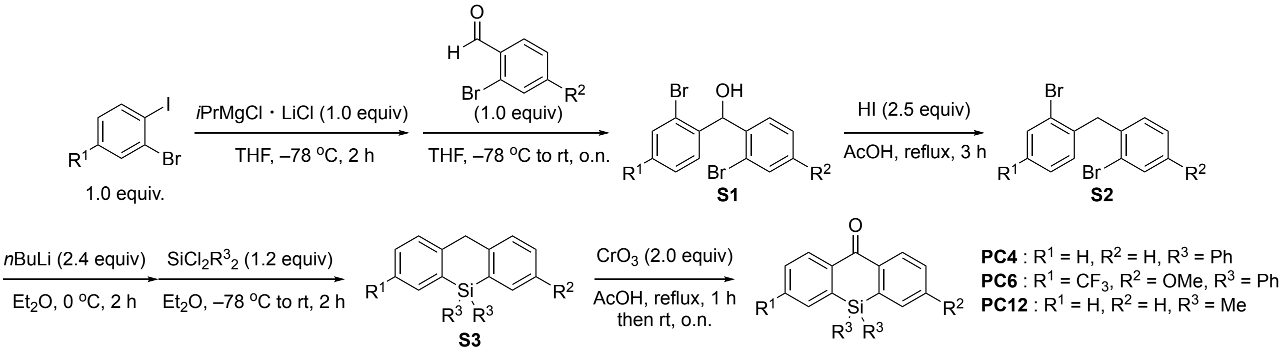

Figure S1. Synthesis of Photocatalysts

Synthesis of PC4 as a representative (Figure S1) To a solution of aryl iodide (626 μL, 5.0 mmol) in dry THF (33.3 mL), *i*PrMgCl･LiCl (1.3 M in THF) (3.85 mL, 5 mmol) was added dropwise at –78 °C. The reaction mixture was stirred for 2 h at –78 °C under a nitrogen atmosphere. In a separate round-bottom flask, THF (6.8 mL) and aldehyde (578 μL, 5.0 mmol) were added, the temperature was cooled to –78 °C, and the prepared Grignard solution was added dropwise. The reaction mixture was stirred for over night at room temperature under a nitrogen atmosphere. The mixture was quenched with NH_4Cl aq. and extracted with Et_2O three times. The organic layer was dried over sodium sulfate and filtered. The crude material was concentrated under reduced pressure. After volatiles were removed, flash column chromatography on silica gel (Biotage Selekt, 100:0–84:16, hexane/EtOAc) gave **S1$_{PC4}$** or **S1$_{PC12}$** (1.46 g, 4.27 mmol, 85%) as a white solid.

To a solution of **S1$_{PC4}$** and **S1$_{PC12}$** (1.46 g, 4.27 mmol) in AcOH (13.8 mL), HI (57 wt% in water) (1.36 mL, 2.42 mmol) was added. The reaction mixture was stirred for 3 h at reflux under a nitrogen atmosphere. The mixture was quenched with Na_2SO_3 aq. and extracted with AcOEt three times. The organic layer was washed with KOH, dried over sodium sulfate, and filtered. The crude material was concentrated under reduced pressure. After volatiles were removed, flash column chromatography on silica gel (Biotage Selekt, hexane only) gave **S2$_{PC4}$** and **S2$_{PC12}$** (1.23 g, 3.79 mmol, 89%) as a red oil.

To a solution of **S2$_{PC4}$** (2.19 g, 6.7 mmol) in dry Et_2O (27 mL), *n*BuLi (1.56 M in hexane) (10.3 mL, 16.1 mmol) was added dropwise at 0 °C. The reaction mixture was stirred for 2 h at 0 °C under a nitrogen atmosphere, and then cooled to –78 °C. In a separate round-bottom flask, Ph_2SiCl_2 (1.66 mL, 8.0 mmol) was added, the temperature was cooled to –78 °C, and the aryl lithium solution was added dropwise. The reaction mixture was stirred for 2 h at room temperature under a nitrogen atmosphere. The mixture was quenched with NH_4Cl aq. and extracted with Et_2O three times. The organic layer was dried over sodium sulfate and filtered. The crude material was concentrated under reduced pressure. After volatiles were removed, the resulting solid was washed with hexane and water. Slightly impure **S3$_{PC4}$** (1.58 g, 4.82 mmol, 72%) was obtained and used in the next reaction without further purification.

To a solution of **S3$_{PC4}$** (360.5 mg, 1.1 mmol) in AcOH (3.7 mL), CrO_3 (220.0 mg, 2.2 mmol) was added. The reaction mixture was stirred for 15 min at room temperature, then refluxed for 1 h, and finally stirred overnight at room temperature under a nitrogen atmosphere. The mixture was quenched with ice water and extracted with Et_2O three times. The organic layer was dried over sodium sulfate

and filtered. The crude material was concentrated under reduced pressure. The resulting solid was passed through a silica gel short-path column with CH_2Cl_2 to give **PC4** (332.3 mg, 0.92 mmol, 83%) as a white solid.

3. Characterization Data for Photocatalysts

Bis(2-bromophenyl)methanol ($\mathbf{S1_{PC4}}$ and $\mathbf{S1_{PC12}}$)

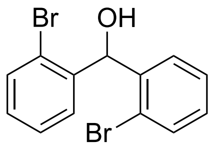

^{1}H NMR (400 MHz, $CDCl_3$) δ 7.59–7.57 (m, 2H), 7.35–7.29 (m, 4H), 7.19 (ddd, J = 8.0, 6.4, 2.4 Hz, 2H), 6.42 (d, J = 4.0 Hz, 1H), 2.55 (dd, J = 4.0, 3.2 Hz, 1H).
^{13}C NMR (100.6 MHz, $CDCl_3$) δ 140.9 (2C), 132.9 (2C), 129.4 (2C), 128.6 (2C), 127.6 (2C), 123.8 (2C), 74.2.
The ^{1}H and ^{13}C NMR spectra data of $\mathbf{S1_{PC4}}$ and $\mathbf{S1_{PC12}}$ were consistent with the literature.[3]

Bis(2-bromophenyl)methane ($\mathbf{S2_{PC4}}$ and $\mathbf{S2_{PC12}}$)

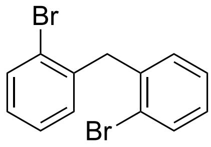

^{1}H NMR (400 MHz, $CDCl_3$) δ 7.60 (dd, J = 8.0, 1.2 Hz, 2H), 7.22 (ddd J = 7.6, 7.6, 1.2 Hz, 2H), 7.11 (ddd, J = 8.0, 7.6, 1.6 Hz, 2H), 6.99 (dd, J = 7.6, 1.6 Hz, 2H) 4.21 (s, 2H).
^{13}C NMR (100.6 MHz, $CDCl_3$) δ 138.9 (2C), 132.8 (2C), 130.7 (2C), 128.1 (2C), 127.5 (2C), 125.1 (2C), 42.1.
The ^{1}H and ^{13}C NMR spectra data of $\mathbf{S2_{PC4}}$ and $\mathbf{S2_{PC12}}$ were consistent with the literature.[3]

5,5-Diphenyldibenzo[*b*,*e*]silin-10(5*H*)-one (PC4)

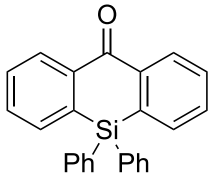

M.p. 190–195 °C.
IR (neat) 1649, 1581, 1428, 1284, 1232, 1132, 1110, 924, 745, 728 cm^{-1}.
^{1}H NMR (400 MHz, $CDCl_3$) δ 8.48–8.46 (m, 2H), 7.72–7.69 (m, 2H), 7.64–7.59 (m, 3H), 7.57–7.54 (m, 5H), 7.45–7.41 (m, 2H), 7.37–7.33 (m, 4H).
^{13}C NMR (100.6 MHz, $CDCl_3$) δ 188.1, 141.9 (2C), 136.0 (4C), 135.6 (2C), 135.0 (2C), 132.8 (2C), 131.9 (2C), 130.5 (2C), 130.2 (2C), 129.7 (2C), 128.2 (4C).

HRMS–ESI (m/z): $[M+H]^+$ calcd for $C_{25}H_{19}OSi^+$, 363.1200; found, 363.1210.

5,5-Dimethyl-5,10-dihydrodibenzo[*b*,*e*]siline ($S3_{PC12}$)

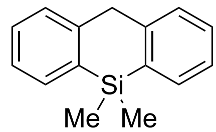

The reaction was carried out in 2.0 mmol scale. The product was purified by flash chromatography on silica gel (Biotage Selekt, hexane only) to afford **$S3_{PC12}$** (300.0 mg, 1.34 mmol, 67%) as a colorless oil.

^{1}H NMR (400 MHz, $CDCl_3$) δ 7.61–7.59 (m, 2H), 7.33–7.31 (m, 4H), 7.29–7.24 (m, 2H), 4.11 (s, 2H), 0.47 (s, 6H).

^{13}C NMR (100.6 MHz, $CDCl_3$) δ 145.9 (2C), 135.7 (2C), 133.0 (2C), 129.0 (2C), 127.9 (2C), 125.6 (2C), 41.5, –3.1 (2C).

The ^{1}H and ^{13}C NMR spectra data of **$S3_{PC12}$** were consistent with the literature.[4]

5,5-Dimethyldibenzo[*b*,*e*]silin-10(5*H*)-one (PC12)

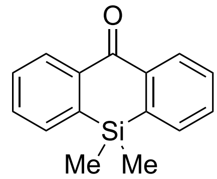

The reaction was carried out in 1.0 mmol scale. The product was purified by flash chromatography on silica gel (Biotage Selekt, 98:2–90:10, hexane/CH_2Cl_2) to afford **PC12** (201.2 mg, 0.84 mmol, 84%) as a white solid.

M.p. 77–82 °C.

IR (neat) 1652, 1583, 1564, 1284, 1232, 1133, 925, 842, 812, 733 cm^{-1}.

^{1}H NMR (400 MHz, $CDCl_3$) δ 8.44–8.41 (m, 2H), 7.70–7.68 (m, 2H), 7.62–7.56 (m, 4H), 0.50 (s, 6H).

^{13}C NMR (100.6 MHz, $CDCl_3$) δ 188.1, 140.9 (2C), 139.0 (2C), 133.2 (2C), 131.7 (2C), 130.0 (2C), 129.6 (2C), –1.5 (2C).

HRMS–ESI (m/z): $[M+H]^+$ calcd for $C_{15}H_{15}OSi^+$, 239.0887; found, 239.0887.

(2-Bromo-4-(trifluoromethyl)phenyl)(2-bromo-4-methoxyphenyl)methanol ($S1_{PC6}$)

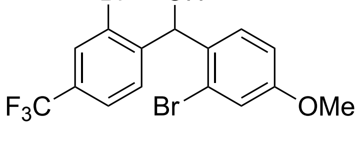

The reaction was carried out in 10.0 mmol scale. The product **$S1_{PC6}$** was purified by flash chromatography on silica gel (Biotage Selekt, 97:3–87:13, hexane/EtOAc) to afford **$S1_{PC6}$** (4.29 g, 9.75 mmol, 97%) as a pale-yellow oil.

IR (neat) 3269, 1602, 1489, 1319, 1231, 1169, 1123, 1079, 1030, 839 cm^{-1}.
^{1}H NMR (400 MHz, $CDCl_3$) δ 7.82 (d, *J* = 0.8 Hz, 1H), 7.68 (d, *J* = 8.0 Hz, 1H), 7.62 (dd, *J* = 8.0, 0.8 Hz, 1H), 7.16 (d, *J* = 2.4 Hz, 1H), 6.99 (d, *J* = 8.8 Hz, 1H), 6.81 (dd, *J* = 8.8, 2.4 Hz, 1H), 6.36 (d, *J* = 4.0 Hz, 1H), 3.80 (s, 3H), 2.55 (m, 1H).
^{13}C NMR (100.6 MHz, $CDCl_3$) δ 159.9, 145.3, 132.3, 131.3 (q, *J* = 33.1 Hz), 129.8 (q, *J* = 3.8 Hz), 129.2, 128.9, 124.6, 124.3 (q, *J* = 3.8 Hz), 123.4, 123.1 (q, *J* = 272.7 Hz), 118.4, 113.6, 73.6, 55.6.
^{19}F NMR (376.5 MHz, $CDCl_3$) δ –62.6.
HRMS–ESI (m/z): $[M+NH_4]^+$ calcd for $C_{15}H_{15}NBr_2F_3O_2^+$, 457.9396; found, 457.9384.

2-Bromo-1-(2-bromo-4-(trifluoromethyl)benzyl)-4-methoxybenzene (S2$_{PC6}$)

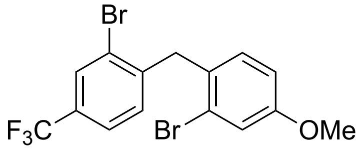

The reaction was carried out in 9.67 mmol scale. The product was purified by flash chromatography on silica gel (Biotage Selekt, 100:0–95:5, hexane/EtOAc) to afford **S1$_{PC6}$** (3.04 g, 7.17 mmol, 74%) as a colorless oil.
IR (neat) 1604, 1567, 1489, 1393, 1317, 1238, 1169, 1122, 1077, 1036 cm^{-1}.
^{1}H NMR (400 MHz, $CDCl_3$) δ 7.85 (d, *J* = 0.8 Hz, 1H), 7.45 (dd, *J* = 8.0, 0.8 Hz, 1H), 7.18 (d, *J* = 2.8 Hz, 1H), 7.02 (d, *J* = 8.0 Hz, 1H), 6.95 (d, *J* = 8.4 Hz, 1H), 6.81 (dd, *J* = 8.4, 2.4 Hz, 1H), 4.17 (s, 2H), 3.80 (s, 3H).
^{13}C NMR (100.6 MHz, $CDCl_3$) δ 159.0, 145.6, 131.3, 130.5, 130.2 (q, *J* = 32.9 Hz), 129.67, 129.65 (q, *J* = 3.8 Hz), 125.2, 124.9, 124.6, 124.2 (q, *J* = 3.8 Hz), 123.2 (q, *J* = 272.5 Hz), 118.2, 55.5, 41.2.
^{19}F NMR (376.5 MHz, CDCl3) δ –62.5.
HRMS–ESI (m/z): $[M+H]^+$ calcd for $C_{15}H_{12}Br_2F_3O^+$, 424.9182; found, 424.9197.

3-Methoxy-5,5-diphenyl-7-(trifluoromethyl)-5,10-dihydrodibenzo[*b*,*e*]siline (S3$_{PC6}$)

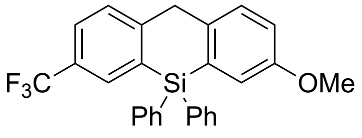

To a solution of **S2$_{PC6}$** (1.11 g, 2.6 mmol) in dry Et_2O (10.4 mL), *n*BuLi (1.51 M in hexane) (4.13 mL, 6.24 mmol) was added dropwise at 0 °C. The reaction mixture was stirred for 2 h at 0 °C under a nitrogen atmosphere, and then cooled to –78 °C. In a separate round-bottom flask, Ph_2SiCl_2 (601.5 μL, 3.12 mmol) was added, the temperature was cooled to –78°C, and the aryl lithium solution was added dropwise. The reaction mixture was stirred for 2 h at room temperature under a nitrogen atmosphere. The mixture was quenched with NH_4Cl aq. and extracted with Et_2O three times. The organic layer was dried over sodium sulfate and filtered. The crude material was concentrated under reduced pressure. After volatiles were removed, filtered using Kiriyama funnel through hexane: Et_2O (9:1) and obtain the filtrate. The filtrate was concentrated under reduced pressure. After volatiles were

removed, flash column chromatography on silica gel (Biotage Selekt, 100:0–90:10, hexane/CH_2Cl_2) gave **S3$_{PC6}$** (801.3 mg, 1.79 mmol, 69%) as a colorless oil.

IR (neat) 1606, 1560, 1484, 1429, 1324, 1252, 1168, 1120, 1080, 1036 cm^{-1}.

^{1}H NMR (400 MHz, $CDCl_3$) δ 7.72 (s, 1H), 7.60 (dd, *J* = 8.0, 1.6 Hz, 1H), 7.50–7.43 (m, 7H), 7.39–7.35 (m, 4H), 7.31 (d, *J* = 8.4 Hz, 1H), 7.03 (d, *J* = 2.8 Hz, 1H), 6.92 (dd, *J* = 8.4, 2.8 Hz, 1H), 4.02 (s, 2H), 3.74 (s, 3H).

^{13}C NMR (100.6 MHz, $CDCl_3$) δ 157.8, 151.1, 137.7, 136.1 (4C), 135.0 (q, *J* = 26.2 Hz), 134.2, 133.4, 131.7, 131.3 (q, *J* = 3.6 Hz), 130.2 (2C), 129.1, 128.1 (4C), 127.8, 126.3 (q, *J* = 3.6 Hz), 124.4 (q, *J* = 272.5 Hz), 120.5 (2C), 114.9, 55.3, 40.6.

^{19}F NMR (376.5 MHz, CDCl3) δ –62.1.

HRMS–ESI (m/z): [M+H]$^+$ calcd for $C_{27}H_{22}F_3OSi^+$, 447.1387; found, 447.1389.

3-Methoxy-5,5-diphenyl-7-(trifluoromethyl)dibenzo[*b*,*e*]silin-10(5*H*)-one (PC6)

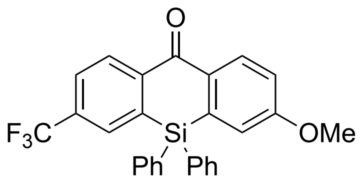

The reaction was carried out in 1.79 mmol scale. The product was purified by flash chromatography on silica gel (Biotage Selekt, 97:3–80:20, hexane/CH_2Cl_2) to afford **PC6** (667.5 mg, 1.45 mmol, 81%) as a white solid.

M.p. 167–172°C.

IR (neat) 1647, 1586, 1429, 1327, 1270, 1247, 1133, 1084, 928, 865 cm^{-1}.

^{1}H NMR (400 MHz, $CDCl_3$) δ 8.59 (d, *J* = 8.4 Hz, 1H), 8.49 (m, 1H), 7.90 (m, 1H), 7.84 (ddd, *J* = 8.4, 2.0, 0.4 Hz, 1H), 7.56–7.53 (m, 4H), 7.49–7.44 (m, 2H), 7.40–7.36 (m, 4H), 7.15–7.11 (m, 2H), 3.84 (s, 3H).

^{13}C NMR (100.6 MHz, $CDCl_3$) δ 185.7, 162.5, 144.6, 137.8, 136.7, 135.9 (4C), 134.3, 133.0, 132.8 (q, *J* = 32.4 Hz), 131.7, 131.4 (q, *J* = 3.6 Hz), 130.6 (2C), 130.1, 128.4 (4C), 127.2 (q, *J* = 3.6 Hz), 123.8 (q, *J* = 272.4 Hz), 119.8 (2C), 116.2, 55.4.

^{19}F NMR (376.5 MHz, CDCl3) δ –63.0.

HRMS–ESI (m/z): [M+H]$^+$ calcd for $C_{27}H_{20}F_3O_2Si^+$, 461.1179; found, 461.1171.

4. Characterization Data for Substrates

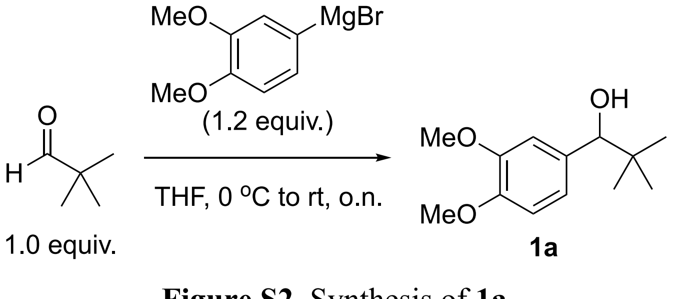

Figure S2. Synthesis of **1a**

Synthesis of 1a as a representative (Figure S2) (Procedure A). Benzyl alcohols were prepared according to the conditions reported in the literature.[1] To a flame-dried 50 mL round-bottom flask equipped with a stir bar was added pivalaldehyde (1.10 mL, 10.0 mmol) and THF (3 mL). The mixture was cooled to 0 °C and a Grignard reagent (0.6 M in THF) (20 mL, 12 mmol) was then added dropwise over 30 min. The reaction mixture was stirred for overnight at room temperature under a nitrogen atmosphere. The reaction was quenched with saturated NH_4Cl (10.0 mL) and extracted with EtOAc three times. The organic layers were combined, dried over Na_2SO_4, and concentrated under reduced pressure. The resultant oil was purified by silica flash chromatography (Biotage Selekt, 97:3–85:15, hexane/EtOAc) to yield benzyl alcohol **1a** as a white solid (1.58 g, 7.06 mmol, 71%).

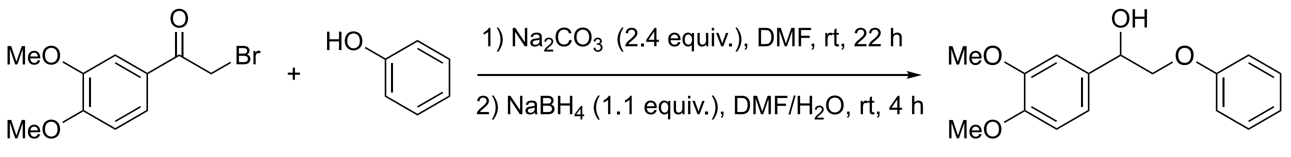

Figure S3. Synthesis of **1h**

Synthesis of 1h as a representative (Figure S3) (Procedure B). Benzyl alcohols were prepared according to the conditions reported in the literature.[1] To a 100 mL round-bottom flask was added Na_2CO_3 (508.8 mg, 4.8 mmol), followed by phenol (225.9 mg, 2.4 mmol) and lastly DMF (9.3 mL). The reaction mixture was stirred at room temperature for 10 min before adding 2-bromo-1-(3,4-dimethoxyphenyl)ethan-1-one (518.2 mg, 2 mmol) in a portion-wise fashion over 10 min. The reaction mixture was further stirred for 22 h at room temperature (monitored by TLC until full conversion). Next, $NaBH_4$ (83.2 mg, 2.2 mmol) dissolved in a 1:1 DMF/H_2O (8 mL) solution was added slowly to the reaction vessel, and subsequently stirred for 4 h. The reaction was carefully quenched with saturated NH_4Cl solution, before extracting the aqueous layer with EtOAc (3x). The organic layer was dried with Na_2SO_4 and concentrated. The crude product was purified by silica flash chromatography (Biotage Selekt, 95:5–75:25, hexane/EtOAc) to yield benzyl alcohol 1h as a white solid (290.5 mg, 1.06 mmol, 53%).

1-(3,4-Dimethoxyphenyl)-2,2-dimethylpropan-1-ol (1a)

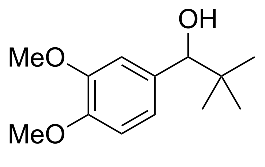

^{1}H NMR (400 MHz, $CDCl_3$) δ 6.88 (s, 1H), 6.83–6.82 (m, 2H), 4.36 (d, J = 2.4 Hz, 1H), 3.88 (s, 3H), 3.88 (s, 3H), 1.80 (d, J = 2.4 Hz, 1H), 0.93 (s, 9H).

^{13}C NMR (100.6 MHz, $CDCl_3$) δ 148.2 (2C), 134.8, 119.9, 110.8, 110.2, 82.2, 55.9 (2C), 35.7, 26.0 (3C).

The ^{1}H and ^{13}C NMR spectra data of **1a** were consistent with the literature.[5]

1-(3,4-Dimethoxyphenyl)-2-phenoxyethan-1-ol (1h)

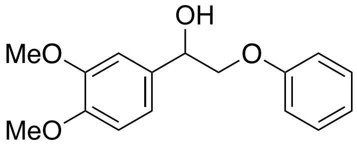

^{1}H NMR (400 MHz, $CDCl_3$) δ 7.32–7.27 (m, 2H), 7.02 (d, J = 2.0 Hz, 1H), 7.00–6.96 (m, 2H), 6.95–6.91 (m, 2H), 6.88 (d, J = 8.4 Hz, 1H), 5.08 (dt, J = 8.8, 2.8 Hz, 1H), 4.09 (dd, J = 9.2, 3.2 Hz, 1H), 4.01 (t, J = 9.2 Hz, 1H), 3.91 (s, 3H), 3.89 (s, 3H), 2.75 (d, J = 2.8 Hz, 1H).
^{13}C NMR (100.6 MHz, $CDCl_3$) δ 158.4, 149.1, 148.9, 132.2, 129.6 (2C), 121.3, 118.6, 114.6 (2C), 111.1, 109.3, 73.3, 72.4, 56.0, 55.9.
The ^{1}H and ^{13}C NMR spectra data of **1h** were consistent with the literature.[6]

1-(3,4-Dimethoxyphenyl)-2-phenylethan-1-ol (1b)

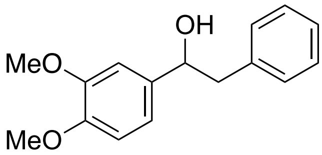

The reaction was carried out with Procedure A in 3.0 mmol scale using Et_2O as solvent instead of THF. The product **1b** was purified by flash chromatography on silica gel (Biotage Selekt, 99:1–85:15, hexane/EtOAc) to afford **1b** (375.2 mg, 1.45 mmol, 48%) as a white solid.
M.p. 50–55 °C.
IR (neat) 3483, 2936, 1594, 1517, 1464, 1454, 1419, 1263, 1234, 1138 cm^{-1}.
^{1}H NMR (400 MHz, $CDCl_3$) δ 7.32–7.28 (m, 2H), 7.23 (m, 1H), 7.20–7.18 (m, 2H), 6.89–6.87 (m, 2H), 6.83 (m, 1H), 4.86 (ddd, J = 7.2, 6.0, 2.4 Hz, 1H), 3.88 (s, 3H), 3.87 (s, 3H), 3.01 (m, 2H), 1.92 (d, J = 2.4 Hz, 1H).
^{13}C NMR (100.6 MHz, $CDCl_3$) δ 148.9, 148.4, 138.0, 136.5, 129.6 (2C), 128.5 (2C), 126.6, 118.1, 110.9, 109.0, 75.2, 55.9, 55.8, 46.1.
HRMS–ESI (m/z): $[M+Na]^+$ calcd for $C_{16}H_{18}O_3Na^+$, 281.1148; found, 281.1147.

Cyclohexyl(3,4-dimethoxyphenyl)methanol (1c)

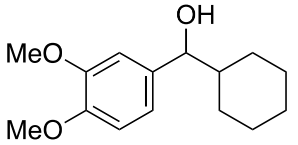

The reaction was carried out with Procedure A in 3.0 mmol scale. The product was purified by flash chromatography on silica gel (Biotage Selekt, 95:5–80:20, hexane/EtOAc) to afford **1c** (364.0 mg, 1.45 mmol, 48%) as a white solid.

^{1}H NMR (400 MHz, $CDCl_3$) δ 6.87 (m, 1H), 6.84–6.80 (m, 2H), 4.30 (d, J = 7.2 Hz, 1H), 3.90 (s, 3H), 3.88 (s, 3H), 2.02 (m, 1H), 1.79–1.76 (m, 2H), 1.68–1.57 (m, 3H), 1.36 (m, 1H), 1.30–0.99 (m, 4H), 0.90 (dq, J = 12.0, 3.2 Hz, 1H).
^{13}C NMR (100.6 MHz, $CDCl_3$) δ 148.9, 148.3, 136.3, 118.9, 110.6, 109.5, 79.3, 55.89, 55.86, 45.0, 29.3, 29.1, 26.4, 26.1, 26.0.
The ^{1}H and ^{13}C NMR spectra data of **1c** were consistent with the literature.[7]

Ethyl 3-(3,4-dimethoxyphenyl)-3-hydroxy-2,2-dimethylpropanoate (1d)

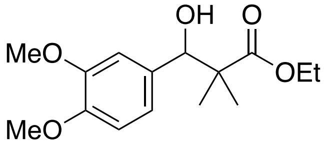

1d were prepared according to the literature.[1]
^{1}H NMR (400 MHz, $CDCl_3$) δ 6.88 (s, 1H), 6.85–6.80 (m, 2H), 4.84 (d, J = 4.0 Hz, 1H), 4.18 (q, J = 7.6 Hz, 2H), 3.88 (s, 6H), 3.15 (m, 1H), 1.27 (td, J = 10.8, 0.8 Hz, 3H), 1.15 (s, 3H), 1.11 (s, 3H).
^{13}C NMR (100.6 MHz, $CDCl_3$) δ 177.9, 148.5, 148.3, 132.6, 120.1, 110.8, 110.3, 78.5, 60.9, 55.8 (2C), 47.6, 23.1, 19.2, 14.1.
The ^{1}H and ^{13}C NMR spectra data of **1d** were consistent with the literature.[5]

3-(3,4-Dimethoxyphenyl)-3-hydroxy-2,2-dimethylpropanoic acid (1e)

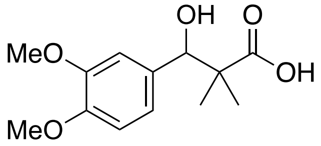

1e were prepared according to the modified procedure in literature.[1] In a 50 mL round-bottom flask, equipped with a magnetic stirring bar, **1d** (2.22 g, 7.86 mmol) was dissolved in THF. To the solution, H_2O and NaOH (628.0 mg, 15.7 mmol) were added, and the obtained mixture was stirred for 4 h at room temperature. The THF was evaporated under reduced pressure and the aqueous residue was diluted with H_2O, acidified with HCl (conc.) to pH 0 and extracted with EtOAc. The combined organic layers were dried over $MgSO_4$, filtered, and evaporated under reduced pressure to afford **1e** (1.80 g, 7.08 mmol, 90%).
^{1}H NMR (400 MHz, $CDCl_3$) δ 6.90–6.82 (m, 3H), 4.88 (s, 1H), 3.88 (m, 6H), 1.18 (s, 3H), 1.17 (s, 3H). The signals for the protons of hydroxy group and carboxy group were not observed.
^{13}C NMR (100.6 MHz, $CDCl_3$) δ 181.4, 148.8, 148.5, 132.0, 120.1, 110.6, 110.5, 78.5, 55.90, 55.88, 47.5, 23.4, 19.1.
The ^{1}H and ^{13}C NMR spectra data of **1e** were consistent with the literature.[5]

1-(3,4-Dimethoxyphenyl)cyclohexan-1-ol (1f)

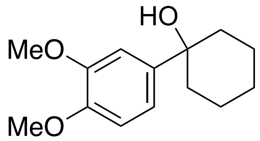

The reaction was carried out with Procedure A. The product was purified by flash chromatography on silica gel (Biotage Selekt, 97:3–85:15, hexane/EtOAc) to afford **1f** (474.0 mg, 2.01 mmol, 20%) as a white solid.

^{1}H NMR (400 MHz, $CDCl_3$) δ 7.11(d, *J* = 2.0 Hz, 1H), 7.01 (dd, *J* = 8.4, 2.0 Hz, 1H), 6.84 (d, *J* = 8.4 Hz, 1H), 3.91 (s, 3H), 3.88 (s, 3H), 1.87–1.70 (m, 7H), 1.66–1.61 (m, 2H), 1.54 (s, 1H), 1.29 (m, 1H).

^{13}C NMR (100.6 MHz, $CDCl_3$) δ 148.7, 147.7, 142.3, 116.5, 110.7, 108.4, 72.9, 55.88, 55.86, 38.9 (2C), 25.5, 22.2 (2C).

The ^{1}H and ^{13}C NMR spectra data of **1f** were consistent with the literature.[8]

1-(3,4-Dimethoxyphenyl)-2-methylcyclohexan-1-ol (1g)

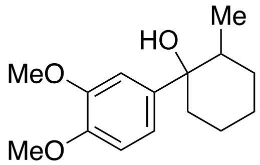

The reaction was carried out with Procedure A. The product was purified by flash chromatography on silica gel (Biotage Selekt, 98:2–85:15, hexane/EtOAc) to afford **1g** (1.38 g, 5.51 mmol, 55%, 1:1 of diastereomixture) as a white solid.

M.p. 59–64 °C.

IR (neat) 3525, 2930, 1511, 1408, 1257, 1166, 1142, 1027, 968, 855 cm^{-1}.

^{1}H NMR (400 MHz, $CDCl_3$) δ 7.03 (d, *J* = 2.0 Hz, 1H), 6.95 (d, *J* = 2.0 Hz, 0.5 × 1H), 6.93 (d, *J* = 2.0 Hz, 0.5 × 1H), 6.84 (m, 1H), 3.90 (s, 3H), 3.88 (s, 3H), 1.90 (m, 1H), 1.80–1.59 (m, 6H), 1.51–1.38 (m, 2H), 0.65 (s, 0.5 × 3H), 0.63 (s, 0.5 × 3H). The signal for the proton of hydroxy group was not observed.

^{13}C NMR (100.6 MHz, $CDCl_3$) δ 148.5, 147.2, 141.3, 116.6, 110.7, 108.4, 75.6, 55.9, 55.8, 41.3, 39.9, 30.5, 26.1, 22.1, 15.6.

HRMS–ESI (m/z): $[M+H]^+$ calcd for $C_{15}H_{23}O_3^+$, 251.1642; found, 251.1635.

1-(3,4-Dimethoxyphenyl)-2-(phenylthio)ethan-1-ol (1i)

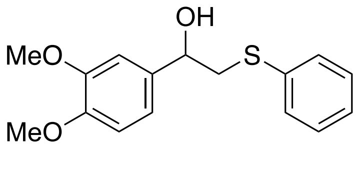

The reaction was carried out with Procedure B. The product was purified by flash chromatography on silica gel (Biotage Selekt, 95:5–75:25, hexane/EtOAc) to afford **1i** (461.0 mg, 1.59 mmol, 79%) as a white solid.

M.p. 70–75 °C.

IR (neat) 3482, 2933, 2834, 1515, 1481, 1464, 1438, 1418, 1260, 1232 cm^{-1}.

^{1}H NMR (400 MHz, $CDCl_3$) δ 7.43–7.40 (m, 2H), 7.34–7.29 (m, 2H), 7.24 (m, 1H), 6.92 (d, *J* = 2.0 Hz, 1H), 6.86 (dd, *J* = 8.4, 2.0 Hz, 1H), 6.83 (d, *J* = 8.4 Hz, 1H), 4.69 (dd, *J* = 9.2, 3.6 Hz, 1H), 3.88 (s, 3H), 3.87 (s, 3H), 3.31 (dd, *J* = 13.6, 3.6 Hz, 1H), 3.12 (dd, *J* = 13.6, 9.2 Hz, 1H), 2.82 (s, 1H).

^{13}C NMR (100.6 MHz, $CDCl_3$) δ 149.1, 148.7, 134.9, 134.7, 130.2 (2C), 129.1 (2C), 126.8, 118.2, 111.0, 108.8, 71.5, 55.91, 55.86, 43.9.

HRMS–ESI (m/z): $[M+H]^+$ calcd for $C_{16}H_{19}O_3S^+$, 291.1049; found, 291.1059.

1-(3,4-Dimethoxyphenyl)-2-(methyl(phenyl)amino)ethan-1-ol (1j)

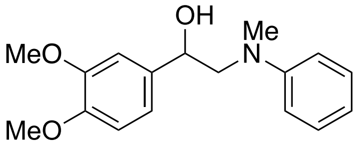

The reaction was carried out with Procedure B. The product was purified by flash chromatography on silica gel (Biotage Selekt, 95:5–75:25, hexane/EtOAc) to afford **1j** (417.6 mg, 1.45 mmol, 73%) as a white solid.

M.p. 90–95 °C.

IR (neat) 3513, 2934, 1599, 1505, 1263, 1234, 1139, 1207, 750, 694 cm^{-1}.

^{1}H NMR (400 MHz, $CDCl_3$) δ 7.29–7.25 (m, 2H), 6.98–6.92 (m, 2H), 6.88–6.84 (m, 3H), 6.78 (m, 1H), 4.95 (ddd, *J* = 8.8, 4.4, 1.6 Hz, 1H), 3.91 (s, 3H), 3.89 (s, 3H), 3.53 (dd, *J* = 14.8, 8.8 Hz, 1H), 3.41 (dd, *J* = 14.8, 4.4 Hz, 1H), 2.94 (s, 3H), 2.47 (d, *J* = 1.6 Hz, 1H).

^{13}C NMR (100.6 MHz, $CDCl_3$) δ 150.0, 149.2, 148.6, 134.6, 129.2 (2C), 118.2, 117.5, 113.3 (2C), 111.1, 108.9, 71.5, 62.0, 55.9 (2C), 39.4.

HRMS–ESI (m/z): $[M+H]^+$ calcd for $C_{17}H_{22}NO_3^+$, 288.1594; found, 288.1587.

(8*R*,9*S*,13*S*,14*S*)-3-(2-(3,4-Dimethoxyphenyl)-2-hydroxyethoxy)-13-methyl-7,8,9,11,12,13,14,15,16,17-decahydro-6*H*-cyclopenta[*a*]phenanthren-17-ol (1k)

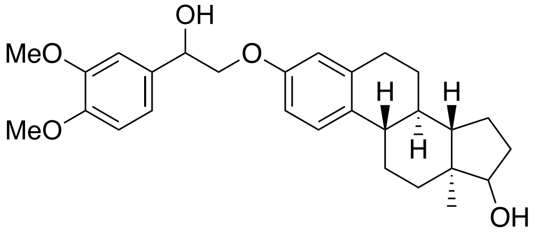

The reaction was carried out with Procedure B in 6.0 mmol scale using K_2CO_3 as base instead of Na_2CO_3. The product was purified by flash chromatography on silica gel (Biotage Selekt, 85:15–65:35, hexane/EtOAc) to afford **1k** (1.37 mg, 3.02 mmol, 50%) as a white solid.

M.p. 117–122 °C.

IR (neat) 3368, 2932, 2855, 1521, 1507, 1255, 1234, 1138, 1087, 1050 cm^{-1}.

^{1}H NMR (400 MHz, $CDCl_3$) δ 7.21 (d, *J* = 8.4 Hz, 1H), 7.01 (d, *J* = 1.8 Hz, 1H), 6.97 (d, *J* = 8.4 Hz, 1H), 6.87 (d, *J* = 8.4 Hz, 1H), 6.73 (dd, *J* = 8.4, 2.8 Hz, 1H), 6.65 (d, *J* = 2.8 Hz, 1H), 5.05 (dd, *J* = 8.8, 3.2 Hz, 1H), 4.06 (m, 1H), 3.96 (m, 1H), 3.91 (s, 3H), 3.89 (s, 3H), 3.73 (t, *J* = 8.4 Hz, 1H), 2.87–2.78 (m, 3H), 2.31 (m, 1H), 2.22–2.07 (m, 2H), 1.95 (m, 1H), 1.87 (m, 1H), 1.70 (m, 1H), 1.54–1.15 (m, 8H), 0.78 (s, 3H).
^{13}C NMR (100.6 MHz, $CDCl_3$) δ 156.2, 149.1, 148.8, 138.2, 133.4, 132.3, 126.4, 118.6, 114.6, 112.2, 111.0, 109.3, 81.9, 73.5, 72.4, 55.93, 55.88, 50.0, 43.9, 43.2, 38.8, 36.7, 30.6, 29.8, 27.2, 26.3, 23.1, 11.0.
HRMS–ESI (m/z): $[M+H]^+$ calcd for $C_{28}H_{37}O_5^+$, 453.2636; found, 453.2633.

1-(3,4-Dimethoxyphenyl)-2-(2-ethoxyphenoxy)propane-1,3-diol (1l)

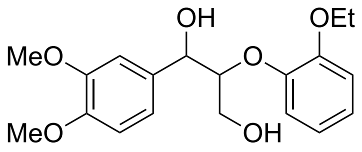

The reaction was carried out with the literature.[9] white solid. The ratio (3:1) of the diastereomers was determined by ^{1}H NMR analysis.
M.p. 75–80 °C.
IR (neat) 3485, 2936, 1593, 1515, 1498, 1454, 1252, 1205, 1139, 1119 cm^{-1}.
^{1}H NMR (400 MHz, $CDCl_3$) δ 7.16–6.81 (m, 7H), 5.00 (m, 1H), 4.17–4.08 (m, 2H + 0.75 × 1H), 3.95–3.88 (m, 6H), 3.79 (m, 0.25 × 1H), 3.66–3.60 (m, 1H + 0.75 × 1H), 3.46 (m, 0.25 × 1H), 2.80 (m, 1H), 1.60 (m, 1H), 1.53–1.46 (m, 3H).
^{13}C NMR (100.6 MHz, $CDCl_3$) δ 151.2, 150.9, 149.0, 148.4, 147.5, 146.9, 132.2, 132.0, 124.5, 121.9, 121.6, 119.7, 118.3, 113.1, 111.0, 109.8, 109.0, 90.1, 88.1, 73.9, 72.5, 64.4, 60.8, 60.6, 55.9, 14.7 (only observed peaks).
HRMS–ESI (m/z): $[M+Na]^+$ calcd for $C_{19}H_{24}O_6Na^+$, 371.1465; found, 371.1454.

1-(4-(Hexyloxy)phenyl)-2,2-dimethylpropan-1-ol (1m)

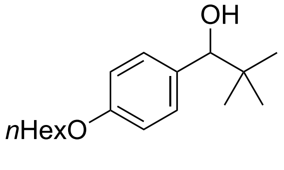

The reaction was carried out with Procedure A in 4.0 mmol scale. The product was purified by flash chromatography on silica gel (Biotage Selekt, 99:1–93:7, hexane/EtOAc) to afford **1m** (588.3 mg, 2.11 mmol, 53%) as a colorless oil.
IR (neat) 3462, 2953, 2932, 2870, 1612, 1511, 1241, 1173, 1005, 832 cm^{-1}.
^{1}H NMR (400 MHz, $CDCl_3$) δ 7.21 (d, *J* = 8.8 Hz, 2H), 6.84 (d, *J* = 8.8 Hz, 2H), 4.35 (d, *J* = 2.8 Hz, 1H), 3.95 (t, *J* = 6.8 Hz, 2H), 1.81–1.74 (m, 3H), 1.49–1.42 (m, 2H), 1.36–1.32 (m, 4H), 0.92–0.89 (m, 12H).

^{13}C NMR (100.6 MHz, $CDCl_3$) δ 158.4, 134.2, 128.6 (2C), 113.5 (2C), 82.0, 68.0, 35.7, 31.6, 29.3, 25.9 (3C), 25.7, 22.6, 14.0.
HRMS–ESI (m/z): $[M+Na]^+$ calcd for $C_{17}H_{28}O_2Na^+$, 287.1982; found, 287.1978.

2,2-Dimethyl-1-(4-phenoxyphenyl)propan-1-ol (1n)

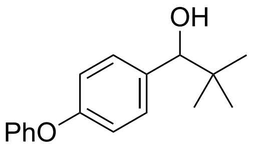

The reaction was carried out with Procedure A in 4.0 mmol scale. The product was purified by flash chromatography on silica gel (Biotage Selekt, 99:1–95:5, hexane/EtOAc) to afford **1n** (716.7 mg, 2.80 mmol, 70%) as a white solid.
M.p. 80–85 °C.
IR (neat) 3421, 2952, 2868, 1589, 1505, 1488, 1363, 1232, 1168, 1046 cm^{-1}.
^{1}H NMR (400 MHz, $CDCl_3$) δ 7.36–7.31 (m, 2H), 7.29–7.26 (m, 2H), 7.10 (m, 1H), 7.03–6.99 (m, 2H), 6.98–6.94 (m, 2H), 4.40 (d, J = 2.8 Hz, 1H), 1.82 (d, J = 2.8 Hz, 1H), 0.93 (s, 9H).
^{13}C NMR (100.6 MHz, $CDCl_3$) δ 157.2, 156.4, 137.0, 129.7 (2C), 128.9 (2C), 123.2, 118.8 (2C), 117.9 (2C), 82.0, 35.7, 25.9 (3C).
HRMS–ESI (m/z): $[M+H]^+$ calcd for $C_{17}H_{21}O_2^+$, 257.1536; found, 257.1527.

1-(6-Methoxynaphthalen-2-yl)-2,2-dimethylpropan-1-ol (1o)

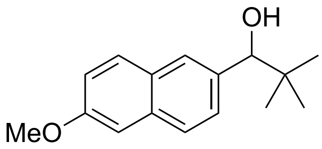

The reaction was carried out with Procedure A. The product **1o** was purified by flash chromatography on silica gel (Biotage Selekt, 99:1–95:5, hexane/EtOAc) to afford **1o** (1.76 g, 7.20 mmol, 72%) as a white solid.
M.p. 109–114 °C.
IR (neat) 3459, 2953, 1633, 1607, 1507, 1482, 1464, 1392, 1363, 1264 cm^{-1}.
^{1}H NMR (400 MHz, $CDCl_3$) δ 7.74–7.68 (m, 3H), 7.43 (dd, J = 8.4, 1.6 Hz, 1H), 7.16–7.13 (m, 2H), 4.54 (d, J = 2.8 Hz, 1H), 3.92 (s, 3H), 1.92 (d, J = 2.8 Hz, 1H), 0.97 (s, 9H).
^{13}C NMR (100.6 MHz, $CDCl_3$) δ 157.6, 137.5, 133.9, 129.5, 128.3, 126.5, 126.1, 125.8, 118.1, 105.5, 82.5, 55.3, 35.9, 26.0 (3C).
HRMS–ESI (m/z): $[M+Na]^+$ calcd for $C_{16}H_{20}O_2Na^+$, 267.1356; found, 267.1353.

1-(4-Hexylphenyl)-2,2-dimethylpropan-1-ol (1p)

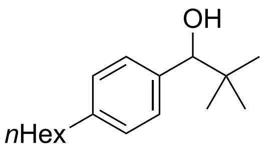

The reaction was carried out with Procedure A in 3.0 mmol scale. The product was purified by flash chromatography on silica gel (Biotage Selekt, 99:1–95:5, hexane/EtOAc) to afford **1p** (437.1 mg, 1.67 mmol, 56%) as a colorless oil.

IR (neat) 3447, 2955, 2927, 2857, 1458, 1363, 1179, 1049, 1005, 905 cm^{-1}.

^{1}H NMR (400 MHz, $CDCl_3$) δ 7.21 (d, J = 8.0 Hz, 2H), 7.12 (d, J = 8.0 Hz, 2H), 4.37 (d, J = 2.8 Hz, 1H), 2.58 (t, J = 7.6 Hz, 2H), 1.79 (d, J = 2.8 Hz, 1H), 1.64–1.58 (m, 2H), 1.36–1.27 (m, 6H), 0.92 (s, 9H), 0.88 (t, J = 6.8 Hz, 3H).

^{13}C NMR (100.6 MHz, $CDCl_3$) δ 142.0, 139.4, 127.6 (2C), 127.5 (2C), 82.3, 35.6, 31.7 (2C), 31.5, 29.0, 25.9 (3C), 22.6, 14.1.

HRMS–ESI (m/z): $[M+H]^+$ calcd for $C_{17}H_{29}O^+$, 249.2213; found, 249.2218.

2,2-Dimethyl-1-(naphthalen-2-yl)propan-1-ol (1q)

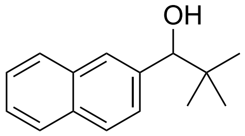

The reaction was carried out with Procedure A in 3.0 mmol scale. The product was purified by flash chromatography on silica gel (Biotage Selekt, 99:1–92:8, hexane/EtOAc) to afford **1q** (514.0 mg, 2.40 mmol, 80%) as a white solid.

M.p. 61–66 °C.

IR (neat) 3444, 2953, 2868, 1479, 1365, 1123, 1051, 1008, 856, 822 cm^{-1}.

^{1}H NMR (400 MHz, $CDCl_3$) δ 7.84–7.76 (m, 4H), 7.50–7.44 (m, 3H), 4.58 (d, J = 2.8 Hz, 1H), 1.95 (d, J = 2.8 Hz, 1H), 0.98 (s, 9H).

^{13}C NMR (100.6 MHz, $CDCl_3$) δ 139.8, 132.83, 132.82, 128.0, 127.6, 127.0, 126.3, 126.0, 125.9, 125.7, 82.5, 36.0, 26.0 (3C).

HRMS–ESI (m/z): $[M+H]^+$ calcd for $C_{15}H_{19}O^+$, 215.1430; found, 215.1434.

2-(4-(Benzyloxy)phenyl)ethan-1-ol (1r)

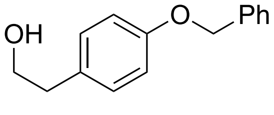

1r were prepared according to the literature.[10]

^{1}H NMR (400 MHz, $CDCl_3$) δ 7.45–7.42 (m, 2H), 7.40–7.36 (m, 2H), 7.32 (m, 1H), 7.17–7.13 (m, 2H), 6.95–6.92 (m, 2H), 5.05 (s, 2H), 3.83 (dd, J = 12.4, 6.4 Hz, 2H), 2.82 (t, J = 6.4 Hz, 2H), 1.36 (t, J = 6.0 Hz, 1H).

^{13}C NMR (100.6 MHz, $CDCl_3$) δ 157.5, 137.1, 130.7, 130.0(2C), 128.6(2C), 127.9, 127.4 (2C), 115.0 (2C), 70.0, 63.8, 38.3.
The ^{1}H and ^{13}C NMR spectra data of **1s** were consistent with the literature.[11]

Ethyl 3-(3,4-dimethoxyphenyl)-2,2-difluoro-3-hydroxypropanoate (1s)

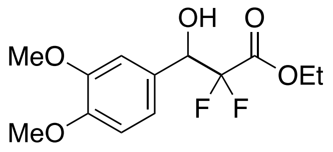

In a 50 mL round-bottom flask, equipped with a magnetic stirring bar, 3,4-dimethoxybenzaldehyde (1.66 g, 10.0 mmol) and ethyl bromodifluoroacetate (1.55 mL, 12.0 mmol) were dissolved in THF (10 mL). $ZnEt_2$ (1.09 M in Hexane) (18.3 mL, 20.0 mmol) was dropwised at 0 ºC, and the obtained mixture was stirred for 40 h at room temperature. Then, acidified with HCl (10%) to pH 7 and extracted with EtOAc. The organic layer was dried with Na_2SO_4 and concentrated. The crude was purified by flash chromatography on silica gel (Biotage Selekt, 85:15–70:30, hexane/EtOAc) to yield benzyl alcohol **1s** as a pale-yellow solid (2.14 g, 7.36 mmol, 74%).
M.p. 55–60 ºC.
IR (neat) 3485, 1755, 1517, 1307, 1261, 1235, 1140, 1070, 1023, 761 cm^{-1}.
^{1}H NMR (400 MHz, $CDCl_3$) δ 7.00–6.97 (m, 2H), 6.87 (d, J = 8.0 Hz, 1H), 7.32 (m, 1H), 5.12 (dd, J = 15.6, 8.0 Hz, 1H), 4.32 (q, J = 7.2 Hz, 2H), 3.90 (s, 3H), 3.89 (s, 3H), 1.32 (t, J = 7.2 Hz, 3H).
^{13}C NMR (100.6 MHz, $CDCl_3$) δ 163.6 (t, J = 31.6 Hz), 149.7, 149.0, 126.7, 120.4, 113.7 (dd, J = 259.4, 253.7 Hz), 110.7, 110.4, 73.6 (dd, J = 27.9, 24.1 Hz), 63.1, 55.89, 55.87, 13.9.
^{19}F NMR (376.5 MHz, CDCl3) δ –114.1 (d, J = 261.1 Hz), –120.6 (d, J = 261.1 Hz).
HRMS–ESI (m/z): $[M+Na]^+$ calcd for $C_{13}H_{16}F_2O_5Na^+$, 313.0849; found, 313.0858.

2,2-Dimethyl-1-phenylpropan-1-ol (1t)

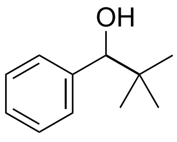

The reaction was carried out with Procedure A in 5.0 mmol scale. The product was purified by flash chromatography on silica gel (Biotage Selekt, 99:1–92:8, hexane/EtOAc) to afford **1t** (711.7 mg, 4.33 mmol, 87%) as a white solid.
M.p. 43–48 °C.
IR (neat) 3439, 2953, 1479, 1452, 1363, 1046, 1005, 899, 734, 702 cm^{-1}.
^{1}H NMR (400 MHz, $CDCl_3$) δ 7.32–7.31 (m, 4H), 7.26 (m, 1H), 4.41 (d, J = 2.8 Hz, 1H), 1.83 (d, J = 2.8 Hz, 1H), 0.93 (s, 9H).
^{13}C NMR (100.6 MHz, $CDCl_3$) δ 142.1, 127.6 (2C), 127.5 (2C), 127.3, 82.4, 35.6, 25.9 (3C).
HRMS–ESI (m/z): $[M+Na]^+$ calcd for $C_{11}H_{16}ONa^+$, 187.1101; found, 187.1093.

1-(3,4-Dimethoxyphenyl)ethan-1-ol (1u)

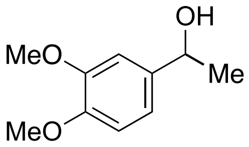

In a 50 mL round-bottom flask, equipped with a magnetic stirring bar, 3,4-dimethoxybenzaldehyde (997.1 mg, 6.0 mmol) was dissolved in THF (10 mL). MeLi (3.1 M in diethoxymethane) (1.6 mL, 5.0 mmol) was dropwised at –78 ºC, and the obtained mixture was stirred for 1 h at room temperature. Then, acidified with NH_4Cl aq and extracted with EtOAc. The organic layer was dried with Na_2SO_4 and concentrated. The crude product was purified by silica flash chromatography (Biotage Selekt, 95:5–70:30, hexane/EtOAc) to yield benzyl alcohol **1u** as a pale-yellow oil (898.4 mg, 4.93 mmol, 99%).

IR (neat) 3394, 2967, 1366, 1515, 1464, 1418, 1257, 1231, 1138, 1024 cm^{-1}.

^{1}H NMR (400 MHz, $CDCl_3$) δ 6.95 (d, J = 1.6 Hz, 1H), 6.90 (dd, J = 8.4, 1.6 Hz, 1H), 6.84 (d, J = 8.4 Hz, 1H), 4.86 (q, J = 6.4 Hz, 1H), 3.91 (s, 3H), 3.88 (s, 3H), 1.79 (bs, 1H), 1.50 (d, J = 6.4 Hz, 3H).

^{13}C NMR (100.6 MHz, $CDCl_3$) δ 149.0, 148.3, 138.5, 117.5, 110.9, 108.5, 70.2, 55.9, 55.8, 25.1.

HRMS–ESI (m/z): $[M+Na]^+$ calcd for $C_{10}H_{14}O_3Na^+$, 205.0834; found, 205.0835.

5. Optimization for *β*-Scission (HAT)

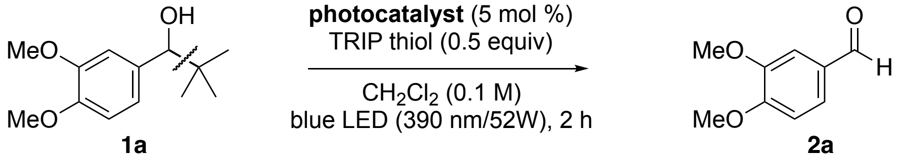

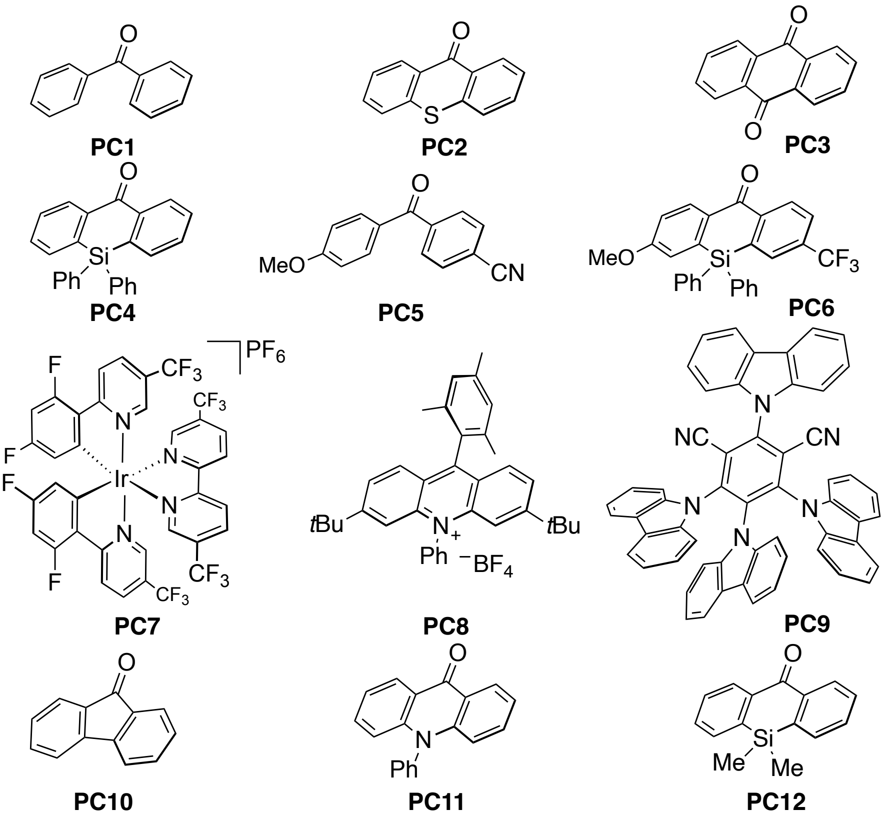

Entry[a]	photocatalyst	yield (%)[b]
1	**PC1**	61
2	**PC2**	83(83)[c]
3	**PC3**	90(65)[c]
4	**PC4**	87(59)[c]
5	**PC5**	77
6	**PC6**	93[d](86)[c]
7	**PC7**	0[c]
8	**PC8**	6[c]
9	**PC9**	17[c]
10	**PC10**	0
11	**PC11**	73
12	**PC12**	85
13	**PC6**	93[e]

[a]Reaction was carried out with **1a** (0.1 mmol), photocatalyst (5 μmol), and TRIP thiol (0.05 mmol) in CH_2Cl_2 (1 mL) under 390 nm blue LED (Kessil lamp) irradiation at ambient temperature for 2 h. [b]^{1}H NMR yield. [c]1 mol% of photocatalyst was used. [d]Isolated yield. [e]The reaction was carried out with Half intesity of LED light.

Table S1. Effect of Photocatalyst

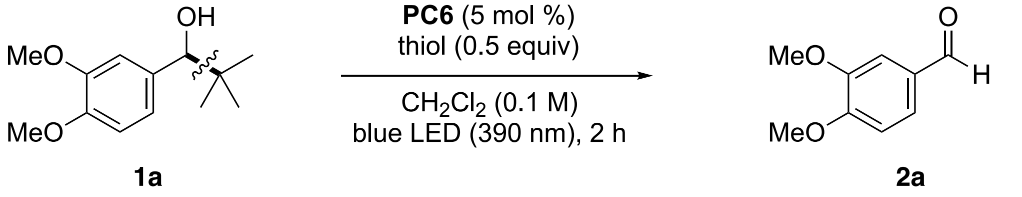

Entry[a]	thiol	yield (%)[b]
1	TRIP thiol	93[c]
2	*n*-dodecanthiol	28
3	C_6H_5SH	84
4	$(i\text{-}Pr)_3SiSH$	12

[a] Reaction was carried out with **1a** (0.1 mmol), **PC6** (5 μmol), and thiol (0.05 mmol) in CH_2Cl_2 (1 mL) under 390 nm blue LED (Kessil lamp) irradiation at ambient temperature for 2 h. [b] ^{1}H NMR yield. [c] Isolated yield.

Table S2. Effect of thiol

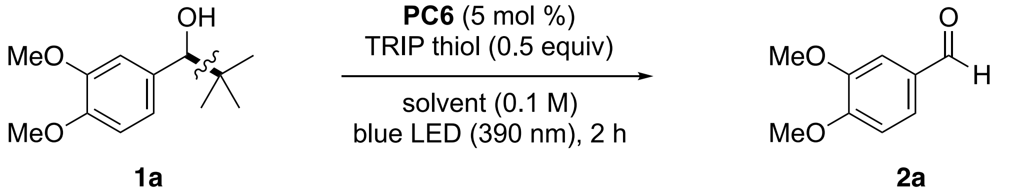

Entry[a]	solvent	yield (%)[b]
1	CH_2Cl_2	93[c]
2	MeCN	80
3	THF	65
4	AcOEt	77

[a] Reaction was carried out with **1a** (0.1 mmol), **PC6** (5 μmol), and TRIP thiol (0.05 mmol) in solvent (1 mL) under 390 nm blue LED (Kessil lamp) irradiation at ambient temperature for 2 h. [b] ^{1}H NMR yield. [c] Isolated yield.

Table S3. Effect of solvent

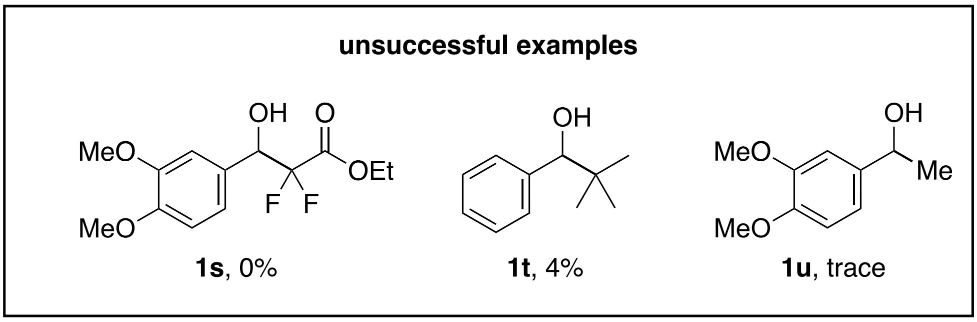

6. Procedure for *β*-Scission (HAT)

The reaction in Table 1, entry 6 is representative.

In a glovebox, a vial equipped with a stirring bar was charged with photoredox catalyst **PC6** (2.3 mg, 5 µmol), benzyl alcohol **1a** (22.4 mg, 0.1 mmol), TRIP thiol (12.6 µL, 0.05 mmol), and CH_2Cl_2 (1.0 mL). After sealing the vial with a cap and removing it from the glovebox, the reaction was stirred and irradiated with a 390 nm blue LED placed 0.5 cm away, with a cooling fan to keep the temperature below 50 °C (Figure S4). After 2 h, the volatileass were removed under reduced pressure, and purification by flash column chromatography on silica gel (97:3–88:12, hexane/EtOAc) gave **2a** (15.5 mg, 0.09 mmol, 93% isolated yield) as a pale-yellow oil.

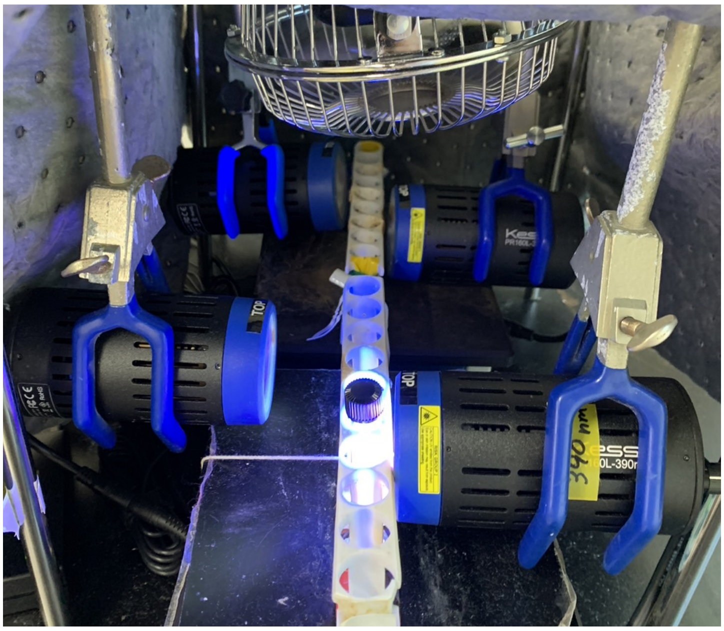

Figure S4. Light set up

7. Characterization Data for Products of *β*-Scission (HAT)

3,4-Dimethoxybenzaldehyde (2a)

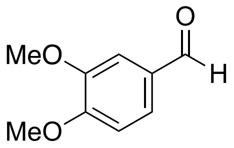

The product was purified by flash chromatography on silica gel (95:5–85:15, hexane/EtOAc) to afford **2a** (Table 1 entry 6; 15.5 mg, 0.093 mmol, 93% isolated yield) as a pale-yellow oil.

^{1}H NMR (400 MHz, $CDCl_3$) δ 9.86 (s, 1H), 7.47 (dd, J = 8.0, 2.0 Hz, 1H), 7.42 (d, J = 2.0 Hz, 1H), 6.99 (d, J = 8.0 Hz, 1H), 3.98 (s, 3H), 3.95 (s, 3H).

^{13}C NMR (100.6 MHz, $CDCl_3$) δ 190.9, 154.5, 149.6, 130.1, 126.9, 110.4, 108.9, 56.2, 56.0.

The ^{1}H and ^{13}C NMR spectra data of **2a** was consistent with the literature.[12]

1-(3,4-Dimethoxyphenyl)hexan-1-one (2f)

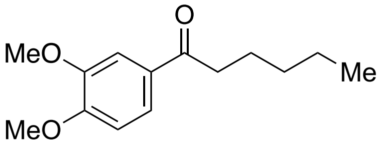

The product was purified by flash chromatography on silica gel (98:2–93:7, hexane/EtOAc) to afford **2f** (Figure 3B; 32,6 mg, 0.14 mmol, 69% isolated yield) as a pale-yellow oil.

^{1}H NMR (400 MHz, $CDCl_3$) δ 7.59 (dd, J = 8.4, 2.0 Hz, 1H), 7.54 (d, J = 2.0 Hz, 1H), 6.89 (d, J = 8.4 Hz, 1H), 3.95 (s, 3H), 3.94 (s, 3H), 2.92 (t, J = 7.6 Hz, 2H), 1.77–1.70 (m, 2H), 1.39–1.34 (m, 4H), 0.93–0.90 (m, 3H).

^{13}C NMR (100.6 MHz, $CDCl_3$) δ199.3, 153.0, 149.0, 130.4, 122.6, 110.2, 109.9, 56.04, 55.95, 38.1, 31.6, 24.5, 22.5, 14.0.

The ^{1}H and ^{13}C NMR spectra data of **2f** were consistent with the literature.[13]

1-(3,4-Dimethoxyphenyl)heptan-1-one (2g)

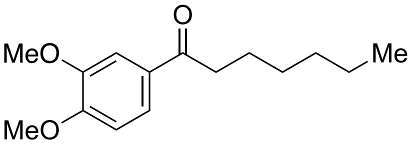

The product was purified by flash chromatography on silica gel (98:2–93:7, hexane/EtOAc) to afford **2g** (Figure 3B; 43.1 mg, 0.17 mmol, 86% isolated yield) as a pale yellow oil.

IR (neat) 2930, 1672, 1586, 1514, 1416, 1343, 1260, 1148, 1132,1023 cm^{-1}.

^{1}H NMR (400 MHz, $CDCl_3$) δ 7.59 (dd, J = 8.4, 2.0 Hz, 1H), 7.54 (d, J = 2.0 Hz, 1H), 6.89 (d, J = 8.4 Hz, 1H), 3.95 (s, 3H), 3.94 (s, 3H), 2.92 (t, J = 7.6 Hz, 2H), 1.73 (tt, J = 7.6, 7.6 Hz, 2H), 1.40–1.29 (m, 6H), 0.89 (t, J = 6.8 Hz, 3H).

^{13}C NMR (100.6 MHz, $CDCl_3$) δ 199.3, 153.1, 149.0, 130.3, 122.6, 110.1, 109.9, 56.0, 55.9, 38.2, 31.7, 29.1, 24.7, 22.5, 14.0.

HRMS–ESI (m/z): $[M+H]^+$ calcd for $C_{15}H_{23}O_3^+$, 251.1642; found, 251.1634.

(8*R*,9*S*,13*S*,14*S*)-3-Methoxy-13-methyl-7,8,9,11,12,13,14,15,16,17-decahydro-6*H*-cyclopenta[*a*]phenanthren-17-ol (2k)

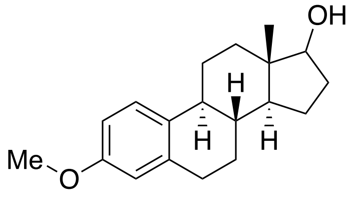

The product was purified by flash chromatography on silica gel (95:5–85:15, hexane/EtOAc) to afford **2k** (Figure 3B; 28.2 mg, 0.098 mmol, 49% isolated yield) as a white solid.

^{1}H NMR (400 MHz, $CDCl_3$) δ 7.21 (d, J = 8.4 Hz, 1H), 6.71 (dd, J = 8.4, 2.8 Hz, 1H), 6.63 (d, J = 2.8 Hz, 1H), 3.78 (s, 3H), 3.73 (t, J = 8.4 Hz, 1H), 2.88–2.84 (m, 2H), 2.32 (m, 1H), 2.22–2.08 (m, 2H), 1.95 (m, 1H), 1.88 (m, 1H), 1.70 (m, 1H), 1.55–1.16 (m, 8H), 0.78 (s, 3H).

^{13}C NMR (100.6 MHz, $CDCl_3$) δ 157.4, 138.0, 132.6, 126.3, 113.8, 111.4, 81.9, 55.2, 50.0, 43.9, 43.2, 38.8, 36.7, 30.6, 29.8, 27.2, 26.3, 23.1, 11.0.

The ^{1}H and ^{13}C NMR spectra data of **2k** were consistent with the literature.[14]

2-(2-Ethoxyphenoxy)ethan-1-ol (2l)

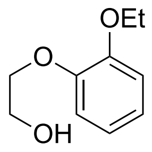

The reaction was carried out using **PC6** (10 mol %). The product **2l** was purified by flash chromatography on silica gel (95:5–85:15, hexane/EtOAc) to afford **2l** (Figure 3B; 11.9 mg, 0.065 mmol, 33% isolated yield) as a colorless oil.

IR (neat) 3416, 2932, 1593, 1501, 1478, 1452, 1392, 1326, 1250, 1214 cm^{-1}.

^{1}H NMR (400 MHz, $CDCl_3$) δ 6.99–6.95 (m, 2H), 6.92–6.87 (m, 2H), 4.14–4.12 (m, 2H), 4.10–4.05 (m, 2H), 3.88 (t, J = 4.4 Hz, 2H), 2.93 (brs, 1H), 1.45 (t, J = 7.2 Hz, 3H).

^{13}C NMR (100.6 MHz, $CDCl_3$) δ 149.6, 148.2, 122.5, 121.1, 116.6, 113.3, 72.3, 64.4, 61.2, 14.8.

HRMS–ESI (m/z): $[M+H]^+$ calcd for $C_{10}H_{15}O_3^+$, 183.1016; found, 183.1010.

4-(Hexyloxy)benzaldehyde (2m)

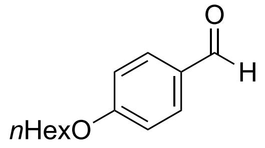

The product was purified by flash chromatography on silica gel (100:0–97:3, hexane/EtOAc) to afford **2m** (Figure 3B; 37.6 mg, 0.18 mmol, 91% isolated yield) as a pale-yellow oil.

^{1}H NMR (400 MHz, $CDCl_3$) δ 9.88 (s, 1H), 7.83 (d, J = 8.8 Hz, 2H), 6.99 (d, J = 8.8 Hz, 2H), 4.04 (t, J = 6.8 Hz, 2H), 1.81 (tt, J = 6.8, 6.8 Hz, 2H), 1.47 (tt, J = 6.8, 6.8 Hz, 2H), 1.37–1.33 (m, 4H), 0.91 (t, J = 6.8 Hz, 3H).

^{13}C NMR (100.6 MHz, $CDCl_3$) δ 190.8, 164.3, 132.0 (2C), 129.7, 114.7 (2C), 68.4, 31.5, 29.0, 25.6, 22.6, 14.0.

The ^{1}H and ^{13}C NMR spectra data of **2m** were consistent with the literature.[15]

4-Phenoxybenzaldehyde (2n)

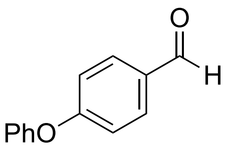

The product **2n** was purified by flash chromatography on silica gel (100:0–97:3, hexane/EtOAc) to afford **2n** (Figure 3B; 33.2 mg, 0.17 mmol, 84% isolated yield) as a pale-yellow oil.

^{1}H NMR (400 MHz, $CDCl_3$) δ 9.93 (s, 1H), 7.87–7.83 (m, 2H), 7.44–7.39 (m, 2H), 7.23 (t, *J* = 7.6 Hz, 1H), 7.09 (d, *J* = 7.6 Hz, 2H), 7.06 (d, *J* = 8.8 Hz, 2H).

^{13}C NMR (100.6 MHz, $CDCl_3$) δ 190.8, 163.2, 155.1, 131.9 (2C), 131.2, 130.1 (2C), 124.9, 120.4 (2C), 117.6 (2C).

The ^{1}H and ^{13}C NMR spectra data of **2n** were consistent with the literature.[16]

6-Methoxy-2-naphthaldehyde (2o)

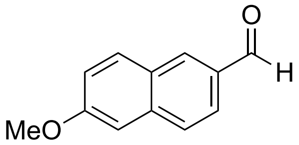

The reaction was carried out using **PC3** instead of **PC6**. The product was purified by flash chromatography on silica gel (97:3–90:10, hexane/EtOAc) to afford **2o** (Figure 3B; 43.6 mg, 0.18 mmol, 93% isolated yield) as a light brown solid.

^{1}H NMR (400 MHz, $CDCl_3$) δ 10.10 (s, 1H), 8.26 (s, 1H), 7.93 (dd, *J* = 8.4, 1.6 Hz, 1H), 7.90 (d, *J* = 8.8 Hz, 1H), 7.81 (d, *J* = 8.4 Hz, 1H), 7.24 (dd, *J* = 8.8, 2.4 Hz, 1H), 7.19 (d, *J* = 2.4 Hz, 1H), 3.97 (s, 3H).

^{13}C NMR (100.6 MHz, $CDCl_3$) δ 192.0, 160.3, 138.3, 134.2, 132.4, 131.1, 128.0, 127.7, 123.7, 120.0, 106.1, 55.5.

The ^{1}H and ^{13}C NMR spectra data of **2o** were consistent with the literature.[17]

4-Hexylbenzaldehyde (2p)

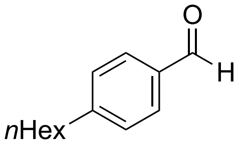

The reaction was carried out using **PC3** (10 mol %) instead of **PC6**. The product was purified by flash chromatography on silica gel (100:0–93:7, hexane/EtOAc) to afford **2p** (Figure 3B; 18.3 mg, 0.096 mmol, 48% isolated yield) as a colorless oil.

^{1}H NMR (400 MHz, $CDCl_3$) δ 9.97 (s, 1H), 7.80 (d, *J* = 8.0 Hz, 2H), 7.34 (d, *J* = 8.0 Hz, 2H), 2.68 (t, *J* = 7.6 Hz, 2H), 1.64 (tt, *J* = 7.6, 7.6 Hz, 2H), 1.37–1.28 (m, 6H), 0.88 (t, *J* = 7.2 Hz, 3H).

^{13}C NMR (100.6 MHz, $CDCl_3$) δ 192.0, 150.5, 134.4, 129.9 (2C), 129.1 (2C), 36.2, 31.6, 31.0, 28.9, 22.5, 14.0.

The ^{1}H and ^{13}C NMR spectra data of **2p** were consistent with the literature.[18]

2-Naphthaldehyde (2q)

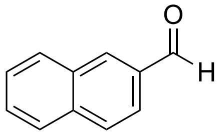

The reaction was carried out using **PC3** instead of **PC6**. The product was purified by flash chromatography on silica gel (100:0–96:4, hexane/EtOAc) to afford **2q** (Figure 3B; 25.3 mg, 0.16 mmol, 81% isolated yield) as a light brown solid.

^{1}H NMR (400 MHz, $CDCl_3$) δ 10.17 (s, 1H), 8.35 (s, 1H), 8.03–7.91 (m, 4H), 7.66 (t, *J* = 7.2 Hz, 1H), 7.60 (t, *J* = 7.2 Hz, 1H).

^{13}C NMR (100.6 MHz, $CDCl_3$) δ 192.3, 136.5, 134.5, 134.1, 132.6, 129.5, 129.1 (2C), 128.1, 127.1, 122.8.

The ^{1}H and ^{13}C NMR spectra data of **2q** were consistent with the literature.[16]

1-(Benzyloxy)-4-methylbenzene (2r)

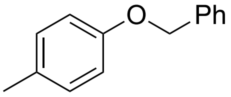

The reaction was carried out using **PC3** (10 mol %) instead of **PC6** and 6 h. The product was purified by flash chromatography on silica gel (100:0–95:5, hexane/EtOAc) to afford **2r** (Figure 3B; 14.1 mg, 0.071 mmol, 36% isolated yield) as a pale-yellow solid.

^{1}H NMR (400 MHz, $CDCl_3$) δ 7.44–7.42 (m, 2H), 7.40–7.36 (m, 2H), 7.31 (m, 1H), 7.08 (d, *J* = 8.4 Hz, 2H), 6.89–6.86 (m, 2H), 5.04 (s, 2H), 2.29 (s, 3H).

^{13}C NMR (100.6 MHz, $CDCl_3$) δ 156.7, 137.2, 130.1 (2C), 129.9 (2C), 128.5, 127.8, 127.4 (2C), 114.7(2C), 70.1, 20.5.

The ^{1}H and ^{13}C NMR spectra data of **2r** were consistent with the literature.[19]

8. Optimization for Giese Addition

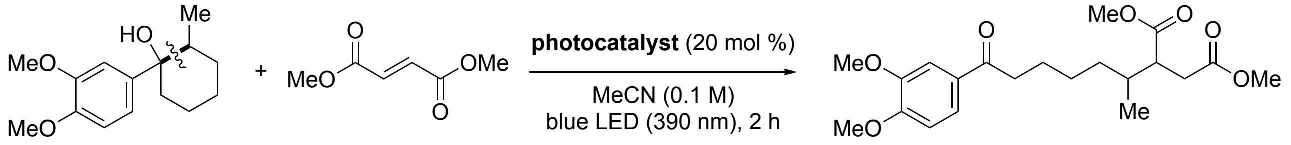

1g 3c 4c

Entry[a]	photocatalyst	yield (%)[b]
1	**PC2**	71
2	**PC3**	28
3	**PC6**	59

[a] Reaction was carried out with **1g** (0.1 mmol), **3c** (0.2 mmol) and photocatalyst (20 μmol) in MeCN (1 mL) under 390 nm blue LED (Kessil lamp) irradiation at ambient temperature for 2 h. [b] ^{1}H NMR yield.

Table S4. Effect of Photocatalyst

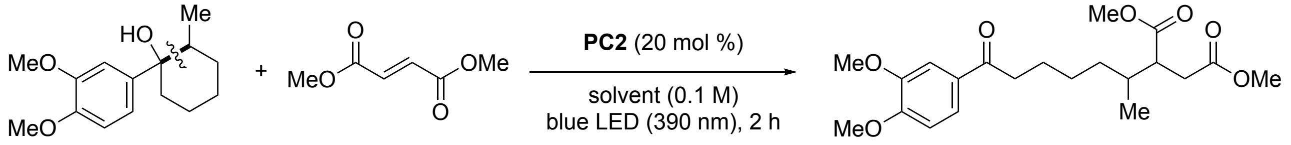

1g 3c 4c

Entry[a]	solvent	yield (%)[b]
1	MeCN	71
2	CH_2Cl_2	52
3	AcOEt	53
4	MeCN : MeOH (9:1)	41

[a] Reaction was carried out with **1g** (0.1 mmol), **3c** (0.2 mmol) and **PC2** (20 μmol) in solvent (1 mL) under 390 nm blue LED (Kessil lamp) irradiation at ambient temperature for 2 h. [b] ^{1}H NMR yield.

Table S5. Effect of Solvent

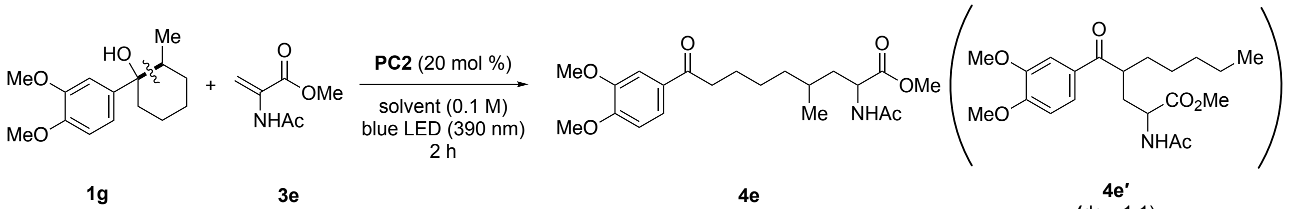

1g 3e 4e 4e′ (dr = 1:1)

Entry[a]	**1g** (mmol)	**3e** (mmol)	solvent	yield (%)[b]
1	0.2	0.4	MeCN	10
2	0.3	0.2	MeCN : MeOH (9:1)	76(77)[c]

[a] Reaction was carried out with **1g**, **3e** and **PC2** (20 μmol) in solvent (1 mL) under 390 nm blue LED (Kessil lamp) irradiation at ambient temperature for 2 h. [b] ^{1}H NMR combined yield of **4e** and **4e′**. [c] Isolated yield.

Table S6. Effect of Substrate Ratio

9. Procedure for Giese Addition

The reaction of 4a in Figure 3C is representative.

In a glovebox, a vial equipped with a stirring bar was charged with photoredox catalyst **PC2** (8.5 mg, 0.04 mmol), benzyl alcohol **1g** (75.1 mg, 0.3 mmol), alkene **3a** (21.8 µL, 0.2 mmol), and MeCN/MeOH (9:1) (2.0 mL). After sealing the vial with a cap and removing it from the glovebox, the reaction was stirred and irradiated with a 390 nm blue LED placed 0.5 cm away, with a cooling fan to keep the temperature below 50 °C (Figure S4). After 2 h, the solvent was evaporated from the reaction solution, and purification by flash column chromatography on silica gel (95:5–85:15, hexane/EtOAc), which gave **4a** (23.9 mg, 0.068 mmol, 34% isolated yield) as a colorless oil.

10. Characterization Data for Products of Giese Addition

Ethyl 9-(3,4-dimethoxyphenyl)-4-methyl-9-oxononanoate (4a)

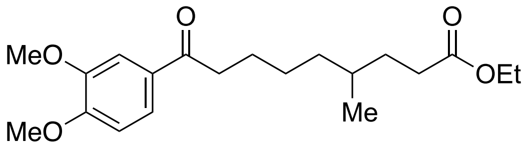

IR (neat) 2932, 1731, 1675, 1586, 1515, 1464, 1416, 1260, 1149, 1023 cm^{-1}.
^{1}H NMR (400 MHz, $CDCl_3$) δ 7.59 (dd, J = 8.4, 2.0 Hz, 1H), 7.54 (d, J = 2.0 Hz, 1H), 6.89 (d, J = 8.4 Hz, 1H), 4.12 (q, J = 7.2 Hz, 2H), 3.95 (s, 3H), 3.94 (s, 3H), 2.92 (t, J = 7.6 Hz, 2H), 2.37–2.22 (m, 2H), 1.75–1.64 (m, 4H), 1.47–1.32 (m, 4H), 1.29–1.17 (m, 4H), 0.88 (d, J = 6.4 Hz, 3H).
^{13}C NMR (100.6 MHz, $CDCl_3$) δ199.1, 174.1, 153.1, 149.0, 130.3, 122.7, 110.2, 109.9, 60.2, 56.04, 55.96, 38.1, 36.5, 32.3, 32.1, 31.8, 26.7, 24.9, 19.2, 14.2.
HRMS–ESI (m/z): $[M+H]^+$ calcd for $C_{20}H_{31}O_5^+$, 351.2166; found, 351.2151.

1-(3,4-Dimethoxyphenyl)-6-methyl-8-(phenylsulfonyl)octan-1-one (4b)

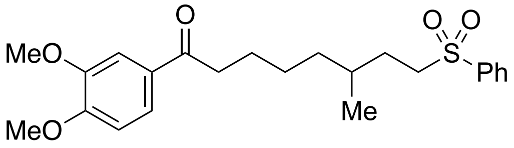

The product was purified by flash chromatography on silica gel (90:10–60:40, hexane/EtOAc) to afford **4b** (Figure 3C; 31.0 mg, 0.074 mmol, 37% isolated yield) as a pale-yellow oil.
IR (neat) 2933, 1672, 1586, 1514, 1446, 1418, 1306, 1260, 1143, 1086 cm^{-1}.
^{1}H NMR (400 MHz, $CDCl_3$) δ 7.93–7.90 (m, 2H), 7.66 (m, 1H), 7.60–7.55 (m, 3H), 7.53 (d, J = 2.0 Hz, 1H), 6.89 (d, J = 8.4 Hz, 1H), 3.95 (s, 3H), 3.94 (s, 3H), 3.18–3.01 (m, 2H), 2.90 (t, J = 7.2 Hz, 2H), 1.79–1.64 (m, 3H), 1.59–1.49 (m, 2H), 1.39–1.24 (m, 3H), 1.18 (m, 1H), 0.85 (d, J = 6.4 Hz, 3H).

^{13}**C NMR** (100.6 MHz, $CDCl_3$) δ 198.9, 153.2, 149.0, 139.2, 133.6, 130.2, 129.2 (2C), 128.0 (2C), 122.6, 110.1, 110.0, 56.03, 55.96, 54.4, 37.9, 36.2, 31.8, 29.1, 26.5, 24.7, 19.1.
HRMS–ESI (m/z): $[M+H]^+$ calcd for $C_{23}H_{31}SO_5^+$, 419.1887; found, 419.1870.

Dimethyl 2-(7-(3,4-dimethoxyphenyl)-7-oxoheptan-2-yl)succinate (4c)

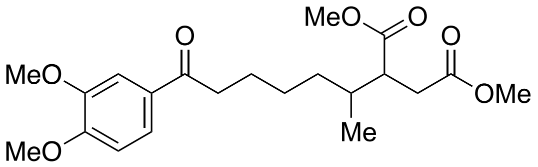

The reaction was carried out using **1g** (50.1 mg, 0.2 mmol), **3c** (57.7 mg, 0.4 mmol), MeCN (2.0 ml). The product was purified by flash chromatography on silica gel (85:15–70:30, hexane/EtOAc) to afford **4c** (Figure 3C; 44.4 mg, 0.11 mmol, 56% isolated yield) as a pale-yellow oil. The ratio (1.2:1) of the diastereomers was determined by ^{1}H NMR analysis.
IR (neat) 2937, 1731, 1672, 1514, 1436, 1416, 1260, 1151, 1021, 750 cm^{-1}.
^{1}**H NMR** (400 MHz, $CDCl_3$) δ 7.58 (dd, J = 8.4, 2.0 Hz, 1H), 7.53 (d, J = 2.0 Hz, 1H), 6.89 (d, J = 8.4 Hz, 1H), 3.95 (s, 3H), 3.94 (s, 3H), 3.692–3.687 (m, 3H), 3.674–3.671 (m, 3H), 2.94–2.85 (m, 3H), 2.75 (m, 1H), 2.35 (td, J = 25.2, 4.0 Hz, 1H), 1.94 (m, 0.55 × 1H), 1.80 (m, 0.45 × 1H), 1.76–1.67 (m, 2H), 1.50–1.17 (m, 4H), 0.91 (d, J = 6.8 Hz, 0.45 × 3H), 0.87 (d, J = 6.8 Hz, 0.55 × 3H).
^{13}**C NMR** (100.6 MHz, $CDCl_3$) δ 198.92, 198.87, 175.0, 174.5, 173.1, 172.9, 153.1, 149.0, 130.3, 122.6, 110.1, 109.9, 56.05, 55.96, 51.78, 51.76, 51.7, 51.6, 45.9, 45.7, 37.9, 35.0, 34.5, 34.2, 33.8, 33.3, 31.4, 27.0, 26.9, 24.62, 24.59, 16.8, 16.2 (only observed peaks).
HRMS–ESI (m/z): $[M+H]^+$ calcd for $C_{21}H_{31}O_7^+$, 395.2064; found, 395.2056.

2-(8-(3,4-Dimethoxyphenyl)-3-methyl-8-oxo-1-phenyloctyl)malononitrile (4d)

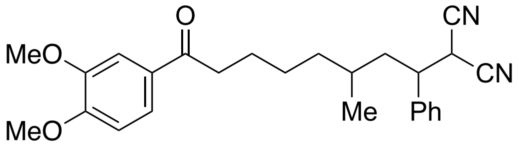

The product was purified by flash chromatography on silica gel (95:5–70:30, hexane/EtOAc) to afford **4d** (Figure 3C; 41.9 mg, 0.10 mmol, 52% isolated yield) as a pale-yellow oil. The ratio (1:1) of the diastereomers was determined by ^{1}H NMR analysis.
IR (neat) 2937, 1670, 1514, 1418, 1261, 1149, 1021, 748, 706, 668 cm^{-1}.
^{1}**H NMR** (400 MHz, $CDCl_3$) δ 7.59 (dd, J = 8.4, 2.0 Hz, 0.5 × 1H), 7.55–7.48 (m, 0.5 × 3H), 7.42–7.37 (m, 3H), 7.34–7.27 (m, 2H), 6.90 (d, J = 8.4 Hz, 0.5 × 1H), 6.87 (d, J = 8.4 Hz, 0.5 × 1H), 4.22 (dd, J = 6.8, 0.4 Hz, 0.5 × 1H), 4.18 (d, J = 5.2 Hz, 0.5 × 1H), 3.96–3.93 (m, 6H), 3.03 (dd, J = 8.4, 6.8 Hz, 0.5 × 1H), 2.98 (dd, J = 7.2, 2.0 Hz, 1H), 2.91 (dd, J = 10.0, 5.2 Hz, 0.5 × 1H), 2.81 (t, J = 2.8 Hz, 1H), 2.25 (m, 1H), 1.78 (m, 1H), 1.67–1.36 (m, 4H), 1.28 (m, 1H), 1.14 (d, J = 6.8 Hz, 0.5 × 3H), 0.82 (d, J = 6.8 Hz, 0.5 × 3H).

^{13}C NMR (100.6 MHz, $CDCl_3$) δ 198.70, 198.66, 153.23, 153.16, 149.1, 149.0, 136.6, 136.1, 130.1, 129.2, 129.1, 128.8, 128.7, 128.4, 128.2, 122.7, 122.6, 112.2, 112.1, 112.0, 111.8, 110.1, 110.03, 109.96, 109.89, 56.06, 56.05, 55.98, 55.96, 52.2, 51.4, 37.63, 37.60, 34.6, 34.1, 33.3, 27.8, 27.4, 26.00, 25.95, 24.5, 24.3, 17.4, 16.3 (only observed peaks).
HRMS–ESI (m/z): $[M+H]^+$ calcd for $C_{25}H_{29}N_2O_3^+$, 405.2173; found, 405.2172.

Methyl 2-acetamido-9-(3,4-dimethoxyphenyl)-4-methyl-9-oxononanoate (4e)

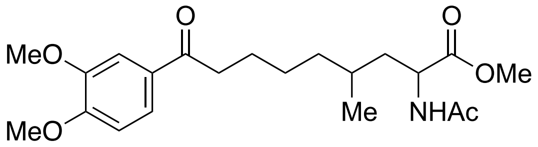

The product was purified by flash chromatography on silica gel (60:40–40:60, hexane/EtOAc) to afford **4e** and **4e′** (Figure 3C; 60.4 mg, 0.15 mmol, 77% isolated yield). **4e** and **4e′** were separated by GPC ($CHCl_3$). Colorless oil. The ratio (1.8:1) of **4e**:**4e′** and the diastereomer ratio of **4e** (1:1) were determined by ^{1}H NMR analysis.
IR (neat) 3365, 2936, 1744, 1655, 1586, 1515, 1418, 1737, 1260, 1148 cm^{-1}.
^{1}H NMR (400 MHz, $CDCl_3$) δ 7.60 (m, 1H), 7.53 (d, J = 2.0 Hz, 1H), 6.89 (d, J = 8.4 Hz, 1H), 5.96 (d, J = 8.4 Hz, 0.6 × 1H), 5.87 (d, J = 8.0 Hz, 0.4 × 1H), 4.66 (m, 1H), 3.95–3.94 (m, 6H), 3.734 (s, 0.4 × 3H), 3.730 (s, 0.6 × 3H), 2.95–2.91 (m, 2H), 2.03 (s, 0.6 × 3H), 2.01 (s, 0.4 × 3H), 1.78–1.65 (m, 2H), 1.59–1.13 (m, 7H), 0.96–0.92 (m, 3H).
^{13}C NMR (100.6 MHz, $CDCl_3$) δ 199.10, 199.07, 173.7, 173.6, 170.0, 169.7, 153.2, 153.1, 149.04, 149.01, 130.30, 130.278, 122.68, 122.66, 110.2, 110.1, 110.0, 56.1, 56.0, 52.3, 50.6, 50.5, 40.1, 39.7, 37.94, 38.87, 36.8, 36.0, 29.4, 29.2, 26.5, 26.4, 24.7, 24.4, 23.2, 23.1, 19.7, 19.3 (only observed peaks).
HRMS–ESI (m/z): $[M+H]^+$ calcd for $C_{21}H_{32}NO_6^+$, 394.2224; found, 394.2210.

Methyl 2-acetamido-4-(3,4-dimethoxybenzoyl)nonanoate (4e′)

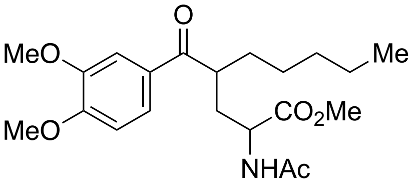

Colorless oil. The diastereomer ratio of **4e′** (1:1) was determined by ^{1}H NMR analysis.
IR (neat) 3283, 2932, 1744, 1659, 1584, 1514, 1419, 1375, 1261, 1021 cm^{-1}.
^{1}H NMR (400 MHz, $CDCl_3$) δ 7.58–7.52 (m, 2H), 6.93–6.90 (m, 1H), 6.07 (d, J = 7.6 Hz, 0.77 × 1H), 5.69 (d, J = 9.2 Hz, 0.23 ×1H), 4.67 (m, 0.23 × 1H), 4.54 (td, J = 7.6, 6.4 Hz, 0.77 × 1H), 3.96 (s, 3H), 3.94 (s, 3H), 3.72 (s, 0.23 × 3H), 3.62 (s, 0.77 × 3H), 3.50 (m, 1H), 2.50 (m, 0.77 × 1H), 2.35 (m, 0.23 × 1H), 2.06 (m, 0.23 × 1H), 1.94 (s, 0.77 × 3H), 1.83 (m, 0.77 × 1H), 1.69 (m, 1H), 1.62 (s, 0.23 × 3H), 1.45 (m, 1H), 1.22 (m, 6H), 0.85–0.82 (m, 3H).

^{13}C NMR (100.6 MHz, $CDCl_3$) δ 202.5, 201.3, 172.8, 172.5, 170.0, 169.6, 153.3, 149.1, 129.8, 122.9, 122.7, 110.5, 110.3, 110.1, 56.04, 55.96, 52.4, 52.3, 51.6, 51.4, 43.0, 42.3, 34.1, 34.0, 33.6, 31.80, 31.76, 26.7, 23.1, 22.8, 22.4, 14.0 (only observed peaks).
HRMS–ESI (m/z): $[M+H]^+$ calcd for $C_{21}H_{32}NO_6^+$, 394.2224; found, 394.2209.

Methyl 2-acetamido-4,4-dimethylpentanoate (4f)

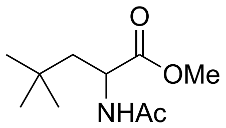

The product was purified by flash chromatography on silica gel (EtOAc) followed by GPC ($CHCl_3$) to afford **4f** (Figure 3C; 39.4 mg, 0.20 mmol, 98% isolated yield) as a colorless oil.
^{1}H NMR (400 MHz, $CDCl_3$) δ 5.79 (brs, 1H), 4.65 (td, J = 13.2, 4.0 Hz, 1H), 3.72 (s, 3H), 2.00 (s, 3H), 1.76 (dd, J = 14.4, 4.0 Hz, 1H), 1.47 (dd, J = 14.4, 8.8 Hz, 1H), 0.96 (s, 9H).
^{13}C NMR (100.6 MHz, $CDCl_3$) δ 174.1, 169.5, 52.3, 49.8, 46.2, 30.7, 29.5 (3C), 23.2.
The ^{1}H and ^{13}C NMR spectra data of **4f** were consistent with the literature.[20]

Methyl 2-acetamido-3-cyclohexylpropanoate (4g)

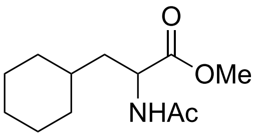

The product **4g** was purified by flash chromatography on silica gel (40:60 hexane/EtOAc) followed by GPC ($CHCl_3$) to afford **4g** (Figure 3C; 37.7 mg, 0.17 mmol, 83% isolated yield) as a colorless oil.
IR (neat) 2923, 1744, 1652, 1541, 1436, 1373, 1261, 1201, 1153, 1008 cm^{-1}.
^{1}H NMR (400 MHz, $CDCl_3$) δ 5.91 (brs, 1H), 4.66 (dt, J = 5.2, 8.8 Hz, 1H), 3.73 (s, 3H), 2.03 (s, 3H), 1.79 (m, 1H), 1.71–1.63 (m, 5H), 1.51 (m, 1H), 1.37–1.08 (m, 4H), 1.00–0.85 (m, 2H).
^{13}C NMR (100.6 MHz, $CDCl_3$) (100.6 MHz, $CDCl_3$) δ 173.8, 169.8, 52.2, 50.1, 40.2, 34.1, 33.4, 32.5, 26.3, 26.1, 25.9, 23.1.
HRMS–ESI (m/z): $[M+H]^+$ calcd for $C_{12}H_{22}NO_3^+$, 228.1594; found, 228.1585.

Methyl *N*-acetyl-*O*-phenylhomoserinate (4h)

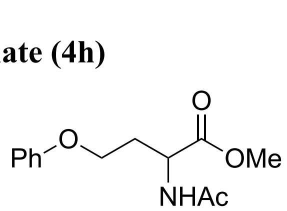

The product was purified by flash chromatography on silica gel (70:30–50:50, hexane/EtOAc) to afford **4h** (Figure 3C; 21.7 mg, 0.086 mmol, 47% isolated yield) as a white solid.
M.p. 105–110 °C.
IR (neat) 3281, 1741, 1652, 1599, 1541, 1497, 1436, 1373, 1240, 1148 cm^{-1}.

^{1}H NMR (400 MHz, $CDCl_3$) δ 7.30–7.28 (m, 2H), 6.96 (t, *J* = 7.2 Hz, 1H), 6.85 (dd, *J* = 8.8, 0.8 Hz, 2H), 6.34 (d, *J* = 4.4 Hz, 1H), 4.76 (dt, *J* = 7.2, 4.4 Hz, 1H), 4.08–3.99 (m, 2H), 3.76 (s, 3H), 2.42–2.28 (m, 2H), 2.03 (s, 3H).
^{13}C NMR (100.6 MHz, $CDCl_3$) δ 172.5, 169.9, 158.2, 129.5 (2C), 121.1, 114.4 (2C), 64.0, 52.5, 50.4, 31.2, 23.2.
HRMS–ESI (m/z): $[M+H]^+$ calcd for $C_{13}H_{18}NO_4^+$, 252.1230; found, 252.1218.

Methyl *N*-acetyl-*S*-phenylhomocysteinate (4i)

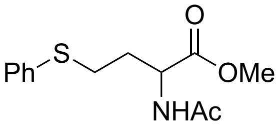

The product was purified by flash chromatography on silica gel (70:30–50:50, hexane/EtOAc) to afford **4i** (Figure 3C; 18.0 mg, 0.067 mmol, 34% isolated yield) as a white solid.
M.p. 111–116 °C.
IR (neat) 3260, 1745, 1637, 1556, 1481, 1438, 1369, 1301, 1230, 1212 cm^{-1}.
^{1}H NMR (400 MHz, $CDCl_3$) δ 7.34–7.27 (m, 4H), 7.20 (m, 1H), 6.11 (s, 1H), 4.73 (dt, *J* = 7.6, 5.2 Hz, 1H), 3.74 (s, 3H), 2.99–2.87 (m, 2H), 2.19 (m, 1H), 2.03 (s, 3H), 1.98 (m, 1H).
^{13}C NMR (100.6 MHz, $CDCl_3$) δ 172.4, 169.9, 135.5, 129.6 (2C), 129.0 (2C), 126.4, 52.6, 51.5, 32.1, 29.8, 23.2.
HRMS–ESI (m/z): $[M+H]^+$ calcd for $C_{13}H_{18}NO_3S^+$, 268.1002; found, 268.0991.

Methyl 2-acetamido-9-(3,4-dimethoxyphenyl)-9-oxononanoate (4j)

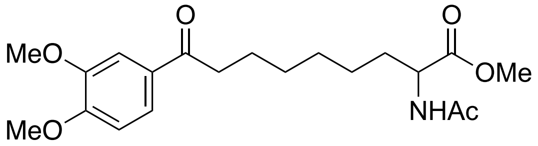

The product was purified by flash chromatography on silica gel (60:40–40:60, hexane/EtOAc) to afford **4j** and **4j′** (Figure 3C; 30.1 mg, 0.079 mmol, 40% isolated yield). **4j** and **4j′** were separated by GPC ($CHCl_3$). colorless oil. The ratio (1:1.9) of **4j**:**4j′** was determined by ^{1}H NMR analysis.
IR (neat) 2934, 1744, 1656, 1586, 1514, 1464, 1418, 1261, 1204, 1149 cm^{-1}.
^{1}H NMR (400 MHz, $CDCl_3$) δ 7.58 (dd, *J* = 8.4, 2.0 Hz, 1H), 7.53 (d, *J* = 2.0 Hz, 1H), 6.89 (d, *J* = 8.4 Hz, 1H), 5.97 (d, *J* = 8.0 Hz, 1H), 4.61 (dt, *J* = 8.0, 5.2 Hz, 1H), 3.95 (s, 3H), 3.94 (s, 3H), 3.74 (s, 3H), 2.91 (t, *J* = 7.2 Hz, 2H), 2.03 (s, 3H), 1.83 (m, 1H), 1.73–1.62 (m, 3H), 1.36–1.26 (m, 6H).
^{13}C NMR (100.6 MHz, $CDCl_3$) δ 199.1, 173.2, 169.7, 153.1, 149.0, 130.3, 122.7, 110.1, 109.9, 56.05, 55.97, 52.3, 52.1, 37.9, 32.4, 29.04, 28.98, 24.9, 24.4, 23.2.
HRMS–ESI (m/z): $[M+H]^+$ calcd for $C_{20}H_{30}NO_6^+$, 380.2068; found, 380.2053.

Methyl 2-acetamido-4-(3,4-dimethoxybenzoyl)octanoate (4j′)

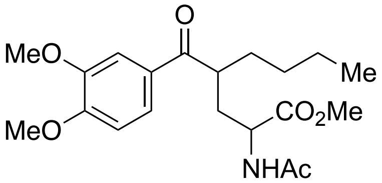

Colorless oil. The ratio (1.3:1) of the diastereomers was determined by ^{1}H NMR analysis.

IR (neat) 2933, 1744, 1655, 1584, 1512, 1438, 1419, 1375, 1261, 1212 cm^{-1}.

^{1}H NMR (400 MHz, $CDCl_3$) δ 7.58–7.53 (m, 2H), 6.91 (dd, J = 8.4, 2.4 Hz, 1H), 6.09 (m, 0.6 × 1H), 5.71 (m, 0.4 × 1H), 4.69 (m, 0.4 × 1H), 4.54 (td, J = 10.8, 7.2 Hz, 0.6 × 1H), 3.96 (s, 3H), 3.94 (s, 3H), 3.72 (s, 0.4 × 3H), 3.62 (s, 0.6 × 3H), 3.49 (m, 1H), 2.50 (m, 0.6 × 1H), 2.35 (m, 0.4 × 1H), 2.06 (m, 0.4 × 1H), 1.94 (s, 0.6 × 3H), 1.84 (m, 0.6 × 1H), 1.68 (m, 1H), 1.62 (s, 0.4 × 3H), 1.48 (m, 1H), 1.25 (brs, 4H), 0.84 (t, J = 6.4 Hz, 3H).

^{13}C NMR (100.6 MHz, $CDCl_3$) δ 202.5, 201.3, 172.8, 172.5, 170.0, 169.6, 153.4, 153.3, 149.12, 149.11, 129.81, 129.80, 122.9, 122.7, 110.5, 110.3, 110.1, 56.05, 56.03, 55.9, 52.4, 52.3, 51.6, 51.4, 42.9, 42.3, 34.1, 34.0, 33.7, 33.4, 29.2, 23.1, 22.8, 22.71, 22.66, 13.82, 13.80 (only observed peaks).

HRMS–ESI (m/z): $[M+H]^+$ calcd for $C_{20}H_{30}NO_6^+$, 380.2068; found, 380.2060.

11. Minisci Reaction

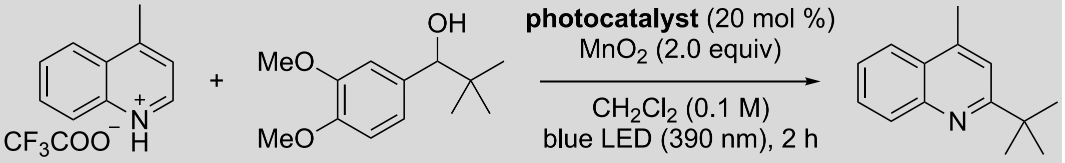

5 **1a** **6**

Entry[a]	photocatalyst	yield (%)[b]
1	**PC2**	55
2	**PC3**	34
3	**PC6**	68(72)[c]

[a] Reaction was carried out with **5** (0.2 mmol), **1a** (0.3 mmol), MnO_2 (0.4 mmol) and photocatalyst (40 μmol) in CH_2Cl_2 (2 mL) under 390 nm blue LED (Kessil lamp) irradiation at ambient temperature for 2 h. [b] ^1H NMR yield. [c] Isolated yield.

Table S7. Optimization of reaction conditions

To a screw-top 4 mL vial with a stirring bar was added lepidine (28.6 mg, 0.2 mmol), MeOH (1.0 mL) and trifluoroacetic acid (34.2 mg, 0.3 mmol). The mixture was stirred for 30 min. Then, the solvent was under reduced pressure. In a glovebox, a vial equipped with a stirring bar was charged with photoredox catalyst **PC** (40 μmol), benzyl alcohol **1a** (67.3 mg, 0.3 mmol), MnO_2 (34.8 mg, 0.4 mmol), and CH_2Cl_2 (2.0 mL). After removing the vial from the glovebox, the reaction was stirred and irradiated with a 390 nm blue LED placed 0.5 cm away, with a cooling fan to keep the temperature below 50 ºC. After stirring for 2 h, the reaction was quenched with saturated K_2CO_3 aq (5 mL) and $Na_2S_2O_3$ aq (5 mL) and extracted with diethyl ether (5 mL × 3). The combined organic layers were dried over Na_2SO_4, filtered, and concentrated under reduced pressure. The residue was purified by flash column chromatography on silica gel (100:0–95:5, hexane/Et_2O) gave **6** (28.5 mg, 0.143 mmol, 72% isolated yield) as a colorless oil.

^1H NMR (400 MHz, $CDCl_3$) δ 8.06 (d, J = 8.4 Hz, 1H), 7.94 (d, J = 8.4 Hz, 1H), 7.66 (t, J = 7.6 Hz, 1H), 7.49 (t, J = 7.6 Hz, 1H), 7.35 (s, 1H), 2.69 (s, 3H), 1.46 (s, 9H).

^{13}C NMR (100,6 MHz, $CDCl_3$) δ 168.9, 147.2, 143.6, 129.9, 128.7, 126.5, 125.4, 123.4, 118.9, 37.9, 30.1 (3C), 19.0.

The ^1H and ^{13}C NMR spectra data of **6** were consistent with the literature.[21]

12. Procedure for Additive Effect

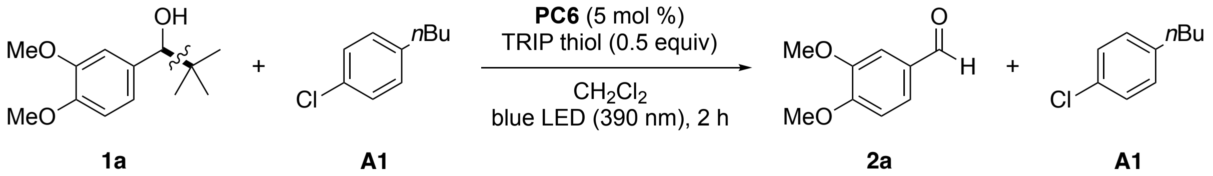

(Supplementary Fig. 4). In a glovebox, a vial equipped with a stirring bar was charged with photoredox catalyst **PC6** (2.3 mg, 0.005 mmol), benzyl alcohol **1a** (22.4 mg, 0.1 mmol), TRIP thiol (12.6 μL, 0.05 mmol), additive **A1** (16.6 mg, 0.98 mmol), and CH_2Cl_2 (1.0 mL). After sealing the vial with a cap and removing it from the glovebox, the reaction was stirred and irradiated with a 390 nm blue LED placed 0.5 cm away, with a cooling fan to keep the temperature below 50 °C (Figure S4). After 2 h, the solvent was removed under reduced pressure, the residue was dissolved in $CDCl_3$ (500 μL), and 1,1,2,2-tetrachloroethane (17.5 mg, 10.4 μmol) was added as an internal standard. ^{1}H NMR (400 MHz) yields of **2a** (90%) and **A1** (quantitative) were determined by comparing the relative integration values of the singlet peak observed at 9.86 ppm of **2a** and the triplet peak observed at 2.57 ppm of **A1** to that of 1,1,2,2-tetrachloroethane observed at 5.96 ppm. Results are summarized in Table S7.

Entry	2a (%)	Additive remaining (%)	Note
A1	90	>99	
A2	95	>99	
A3	91	>99	
A4	88	98	
A5	88	70	
A6	96	>99	
A7	92	>99	
A8	83	>99	
A9	88	>99	
A10	90	>99	
A11	94	>99	
A12	94	>99	
A13	68	78	

Entry	2a (%)	Additive remaining (%)	Note
A14	51	0	celite short pass with CH_2Cl_2.
A15	89	>99	
A16	87	99	
A17	84	>99	1,2-dibromoethane instead of 1,1,2,2-tetrachloroethane.
A18	53	77	
A19	89	>99	
A20	73	91	
A21	94	>99	
A22	91	>99	
A23	67	84	
A24	58	90	
A25	76	>99	
A26	96	33	

Table S8. Result on Additive Effect

13. UV/Vis Spectra of Photocatalysts

UV-Vis absorption spectra of organophotocatalysts were measured with 200 μM acetonitrile solution.

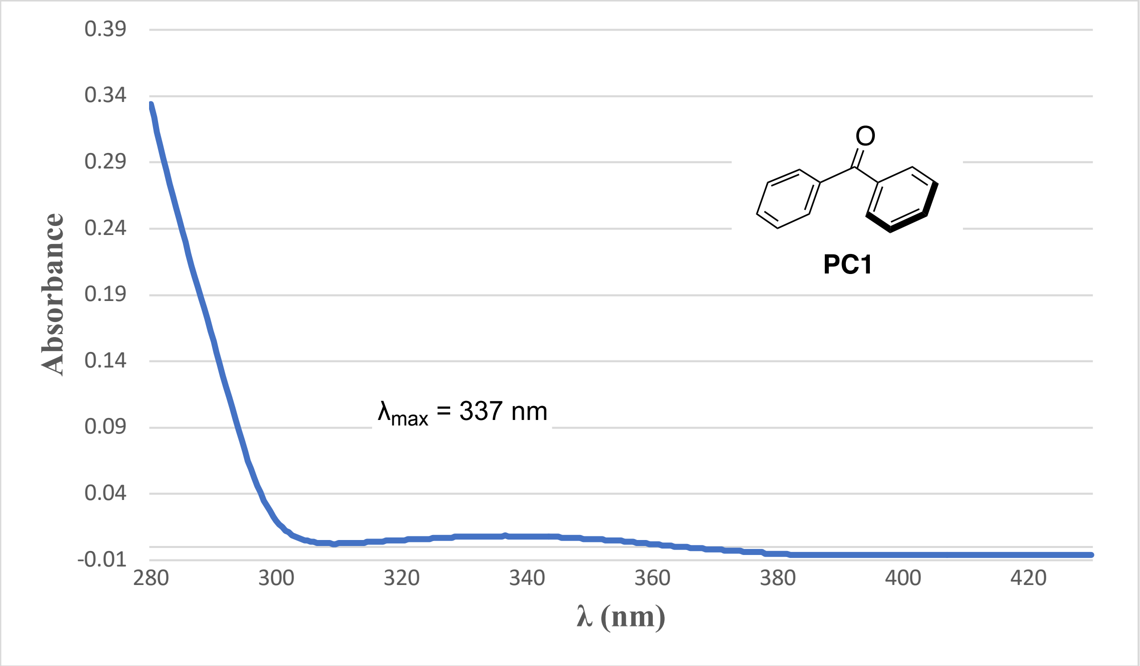

Figure S5. UV-Vis spectrum of **PC1**.

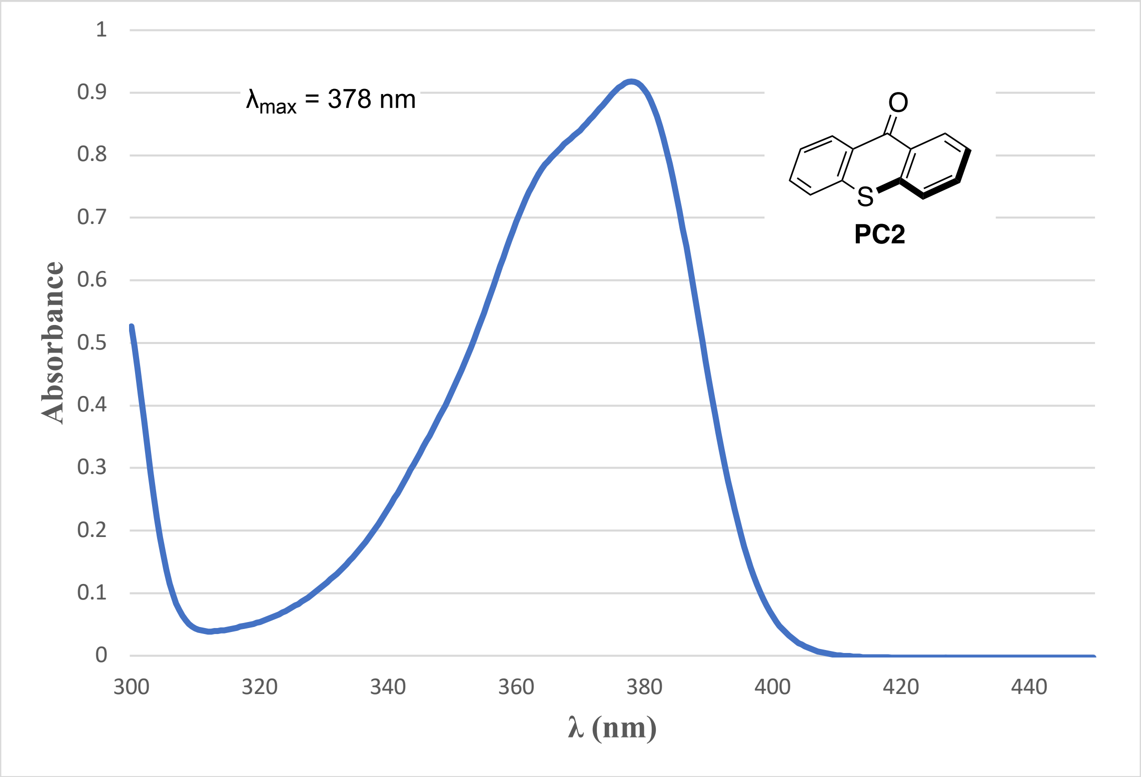

Figure S6. UV-Vis spectrum of **PC2**.

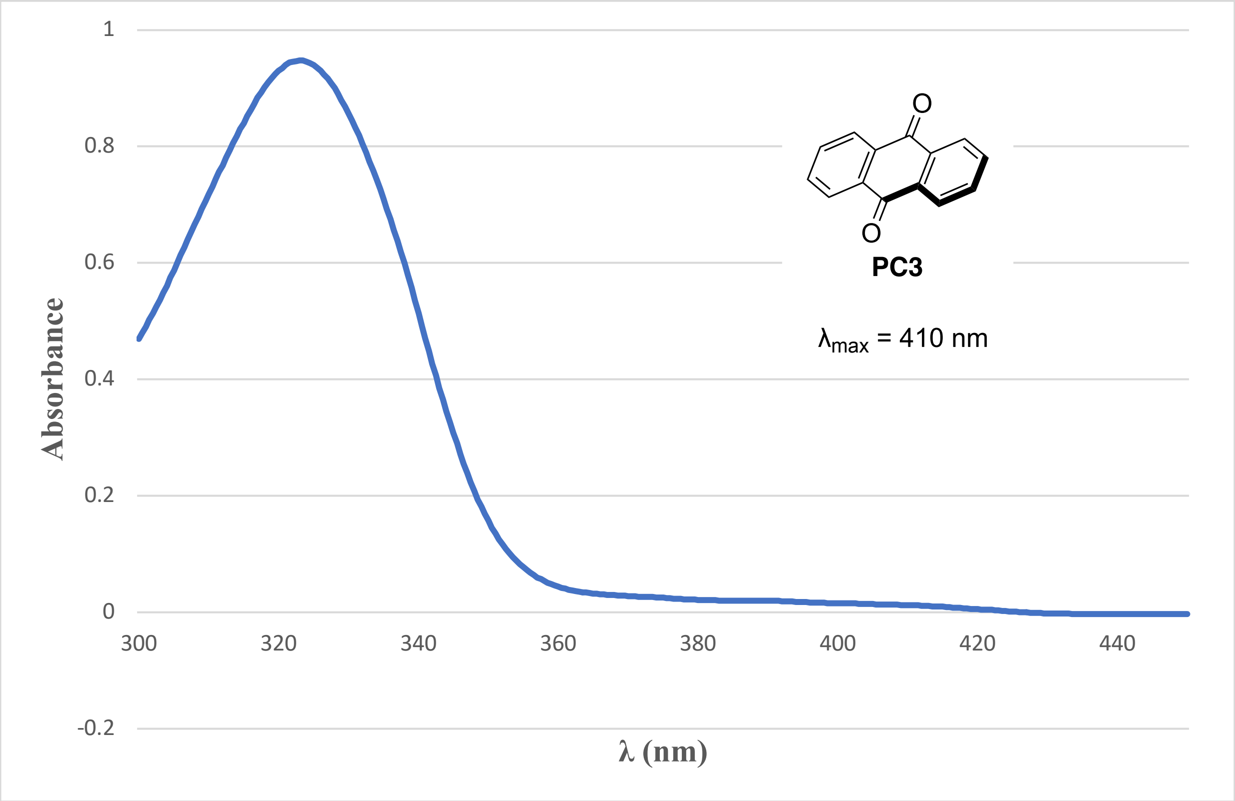

Figure S7. UV-Vis spectrum of **PC3**.

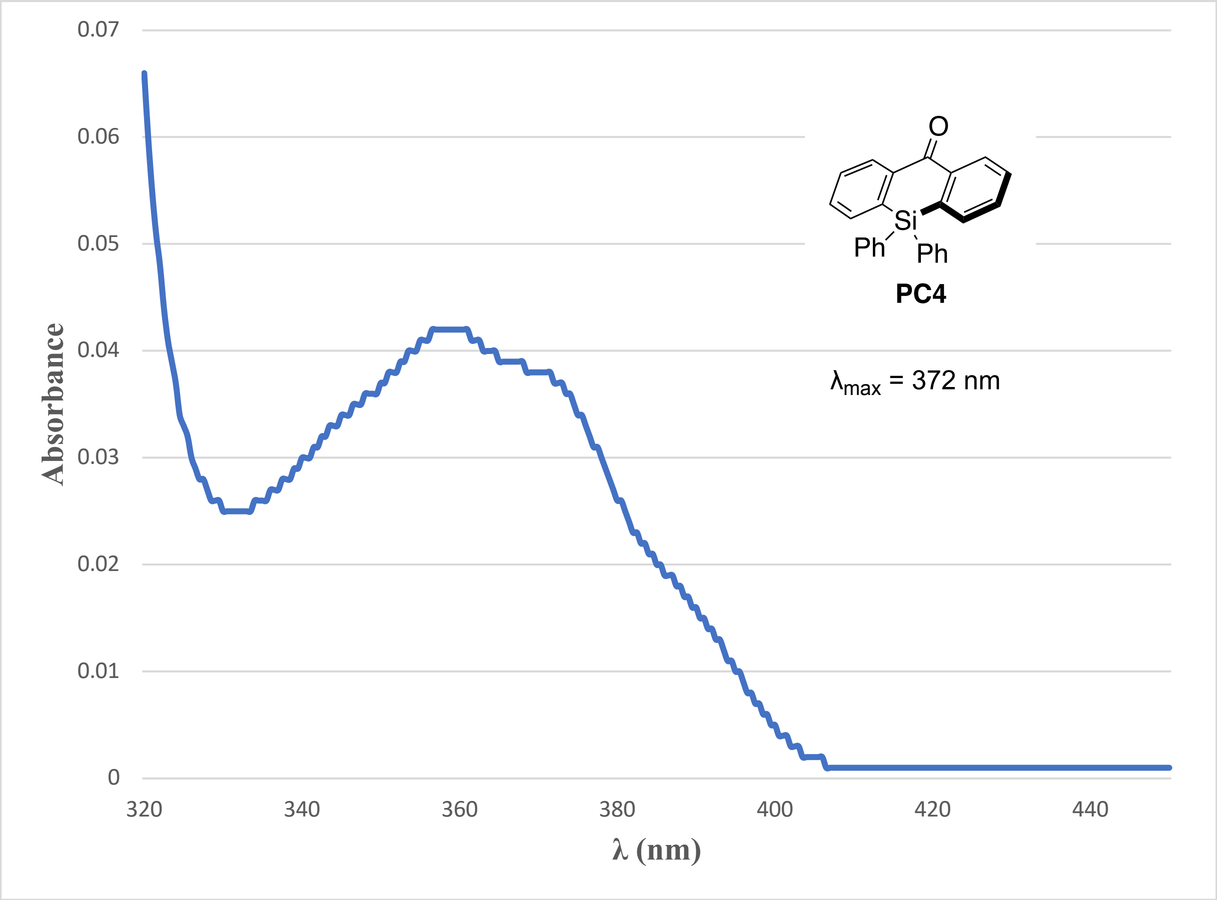

Figure S8. UV-Vis spectrum of **PC4**.

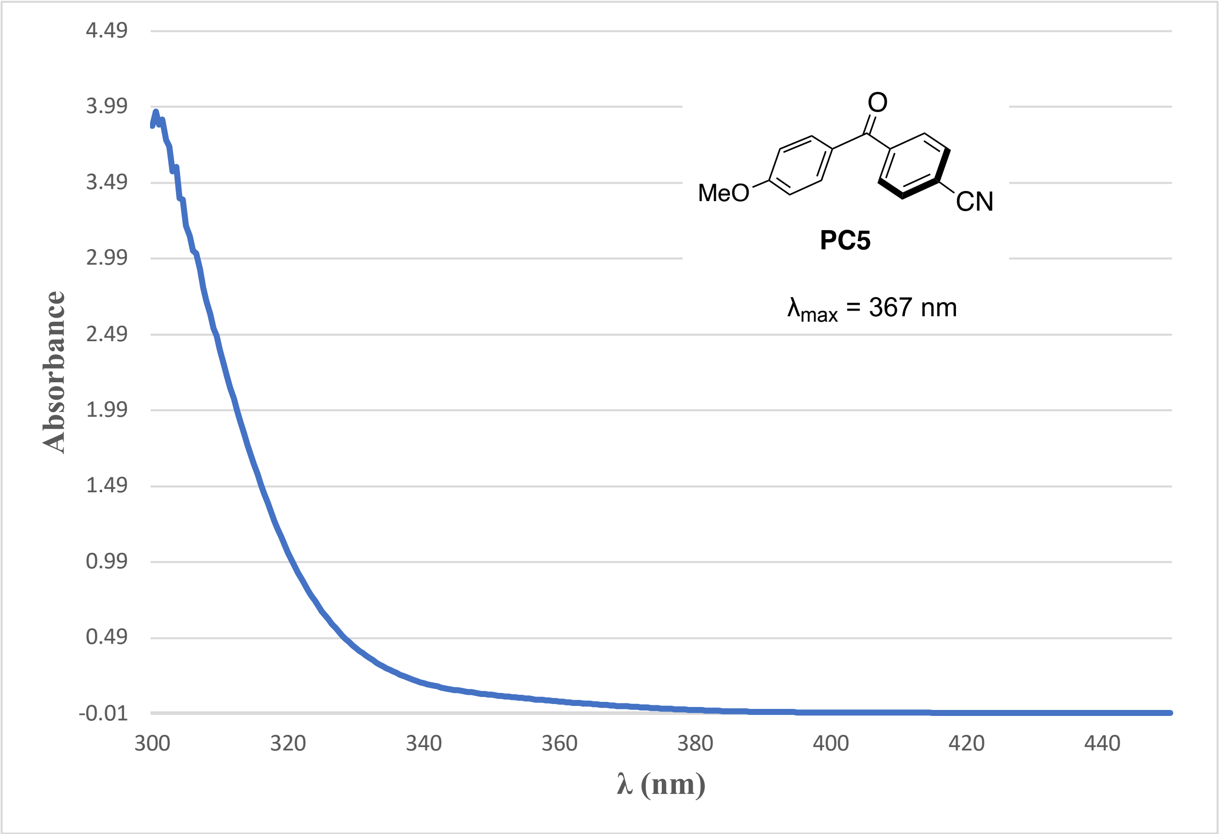

Figure S9. UV-Vis spectrum of **PC5**.

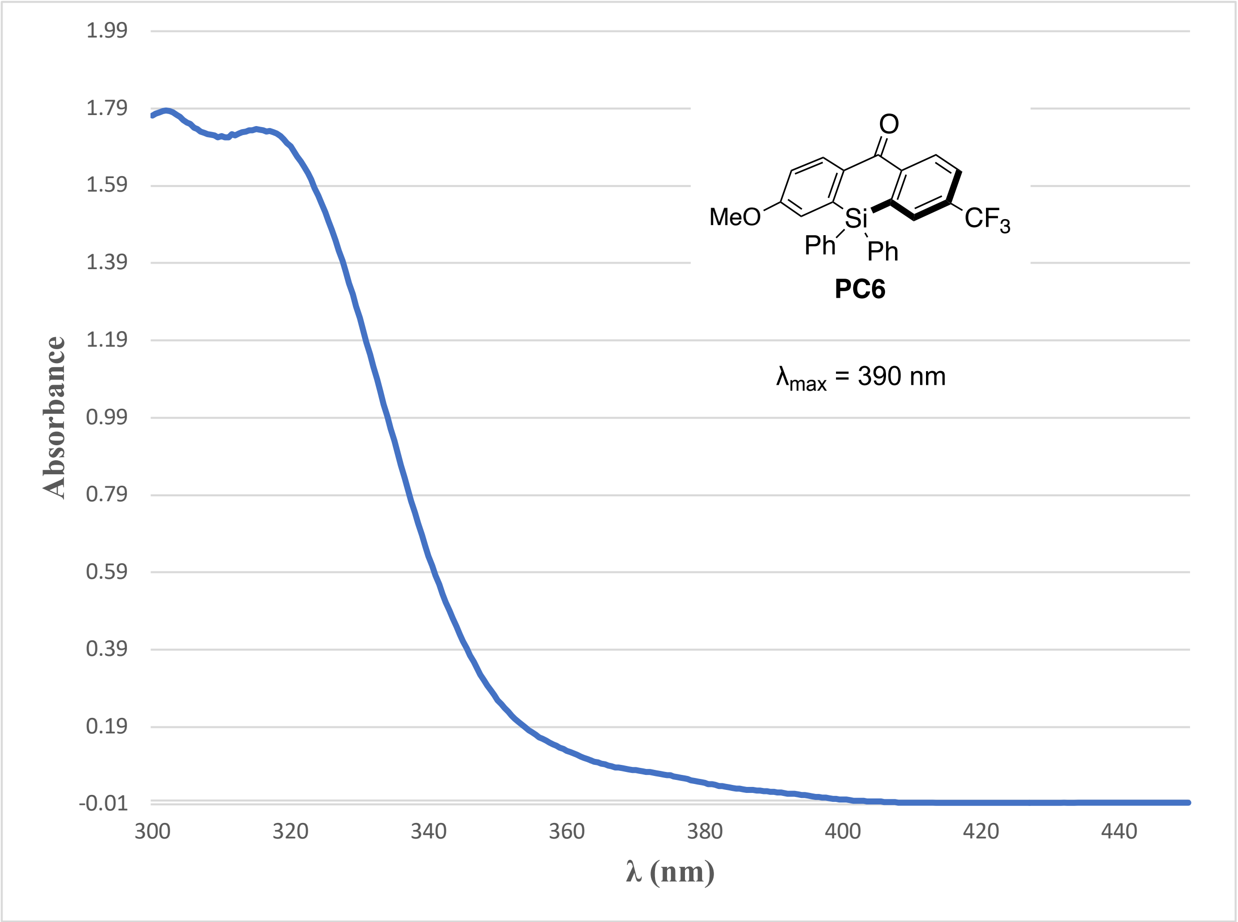

Figure S10. UV-Vis spectrum of **PC6**.

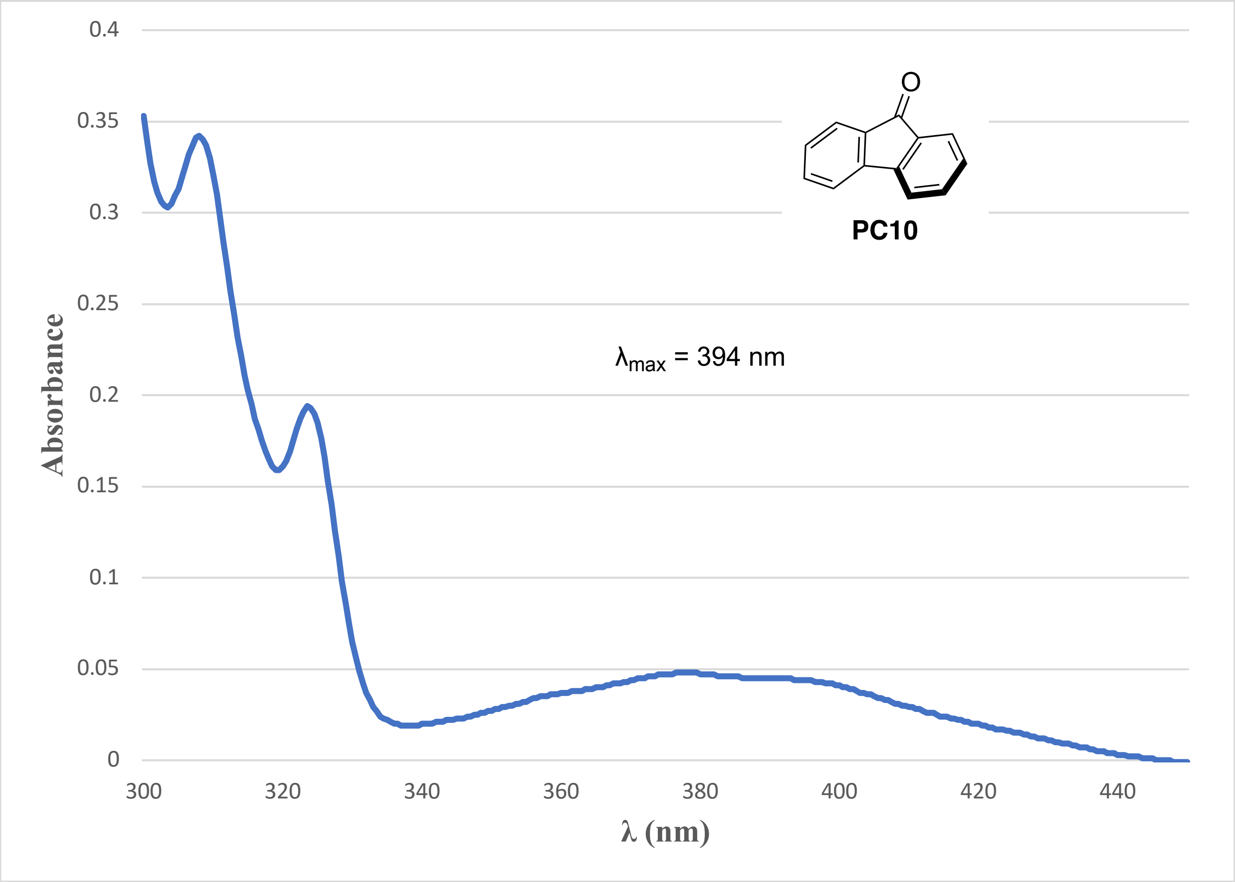

Figure S11. UV-Vis spectrum of **PC10**.

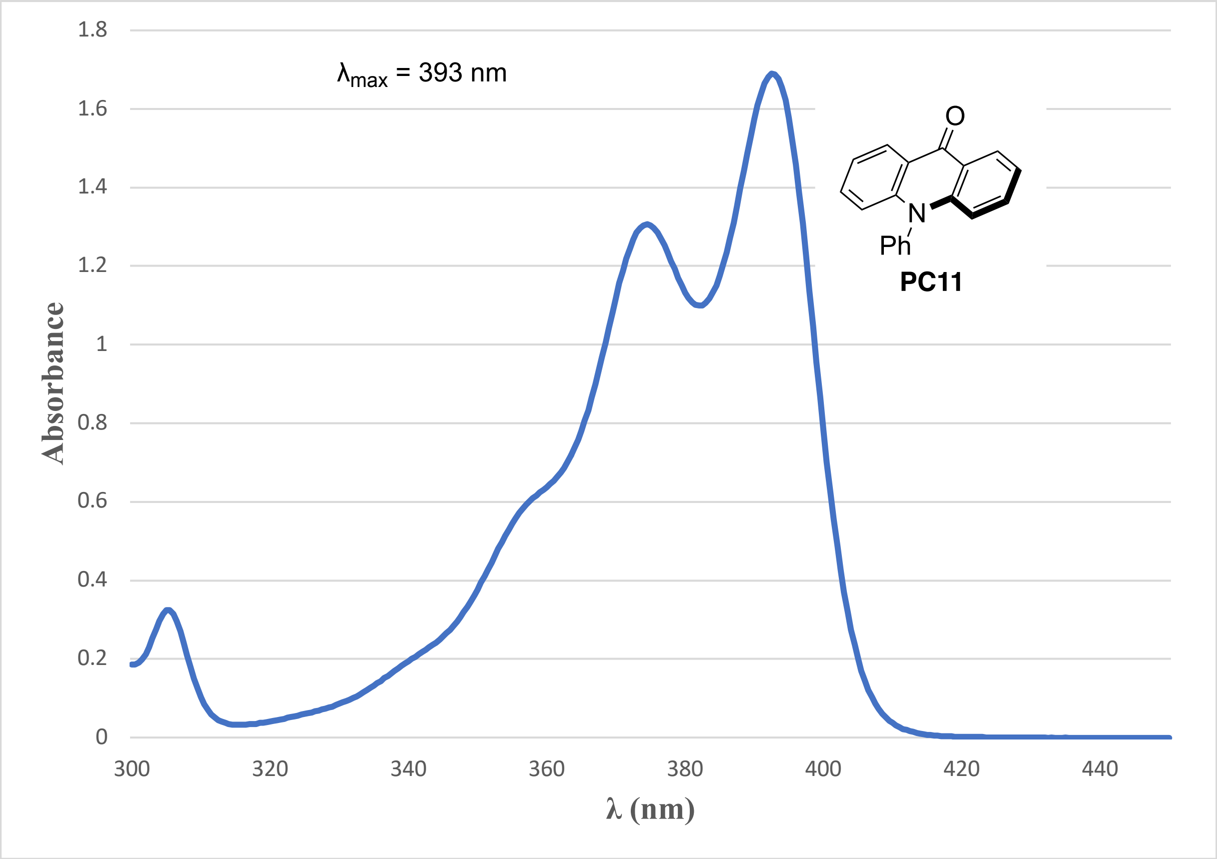

Figure S12. UV-Vis spectrum of PC11.

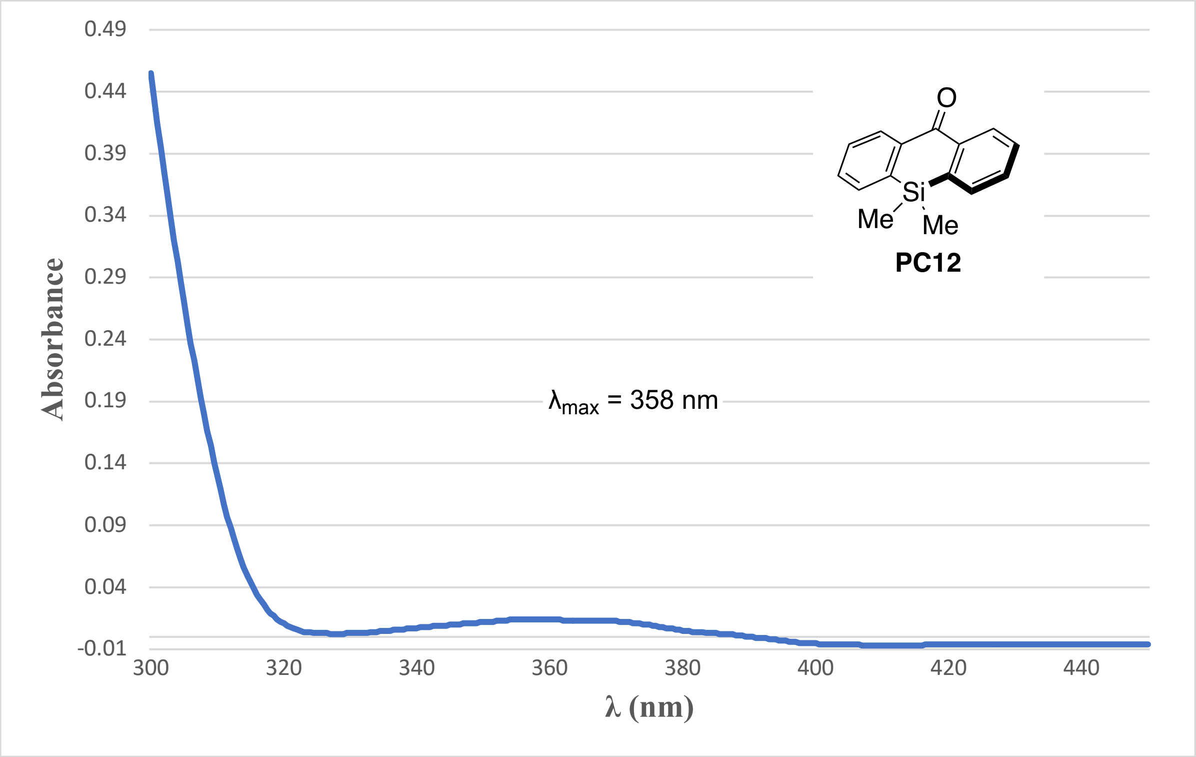

Figure S13. UV-Vis spectrum of **PC12**.

14. Fluorescence Spectra of Photocatalysts

Fluorescence spectra of organophotocatalysts were measured with 200 µM acetonitrile solution.

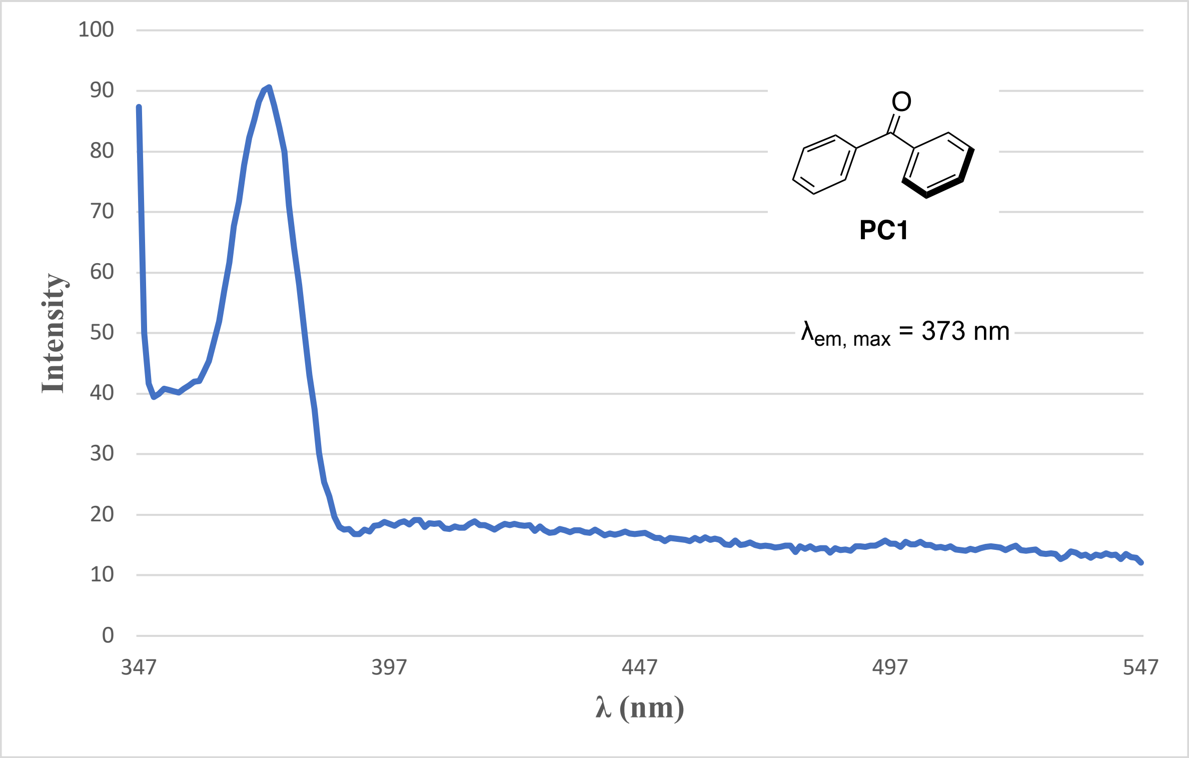

Figure S14. Fluorescence spectrum of **PC1** was scanned after excitation at 337 nm.

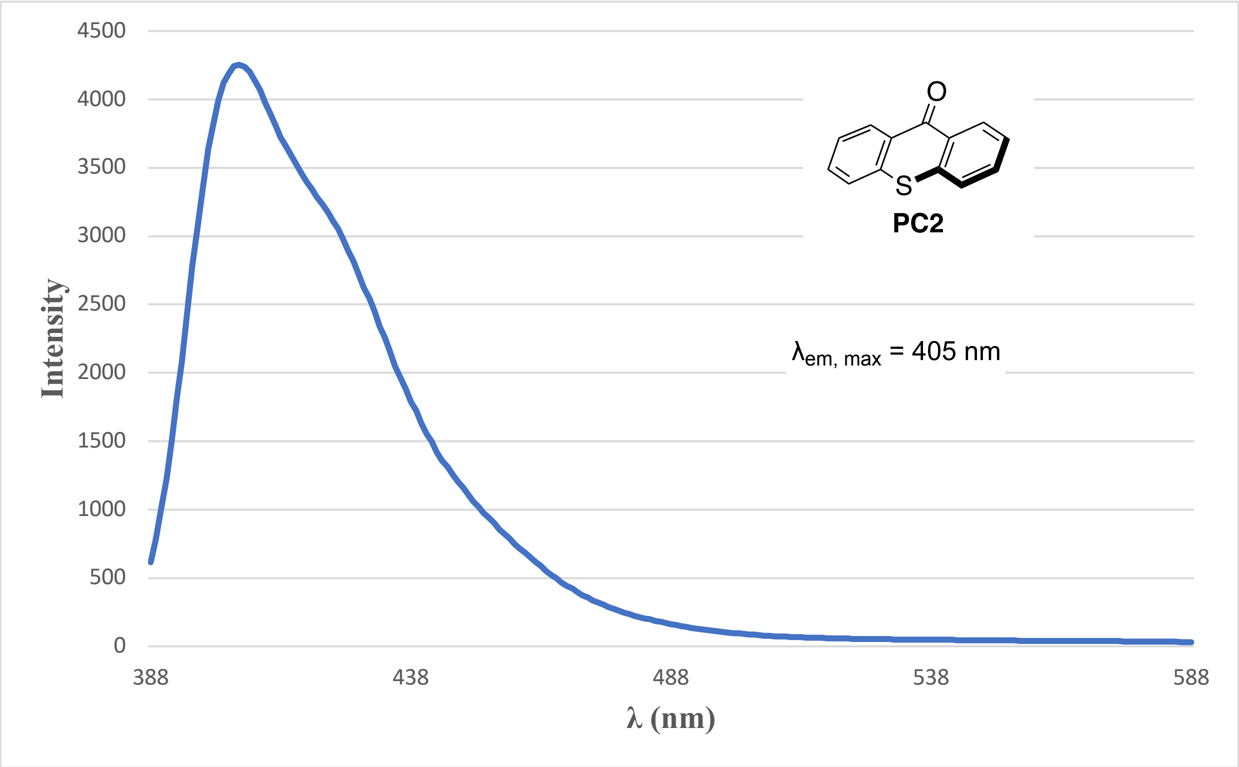

Figure S15. Fluorescence spectrum of **PC2** was scanned after excitation at 378 nm.

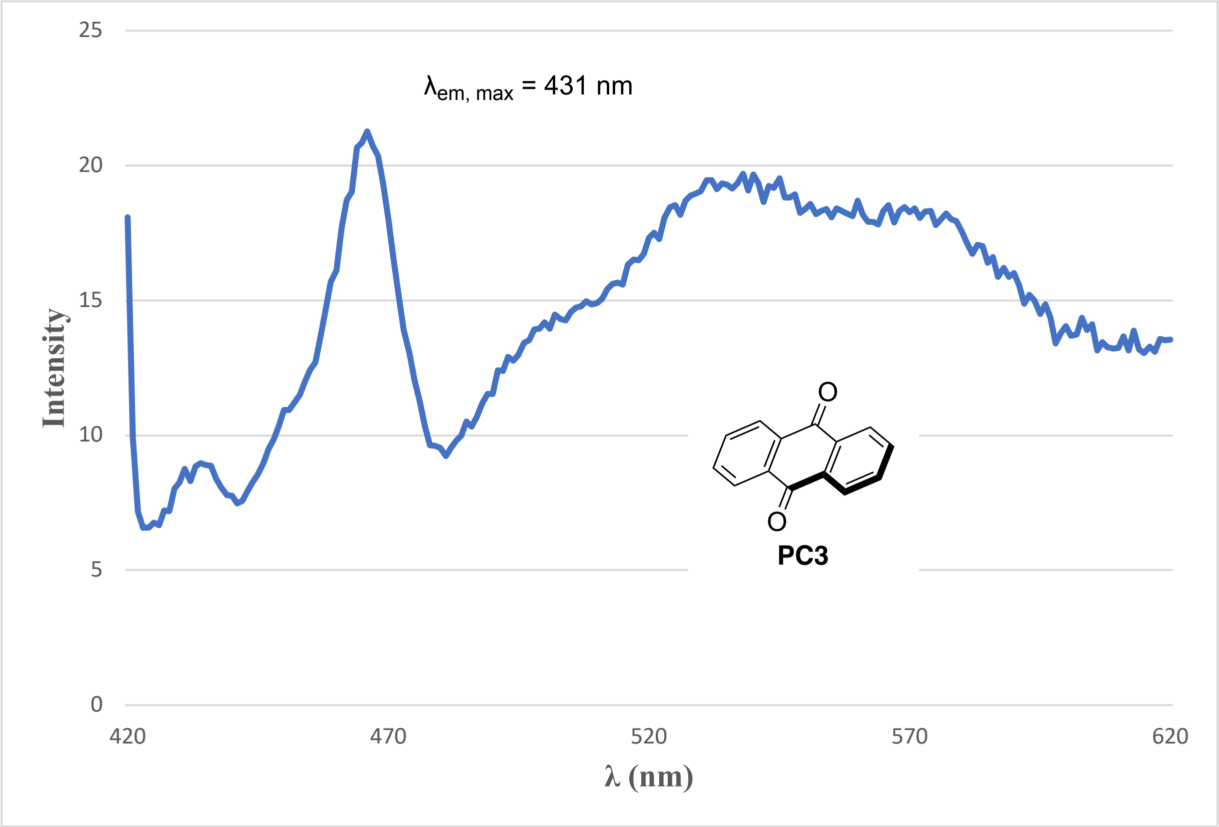

Figure S16. Fluorescence spectrum of **PC3** was scanned after excitation at 410 nm.

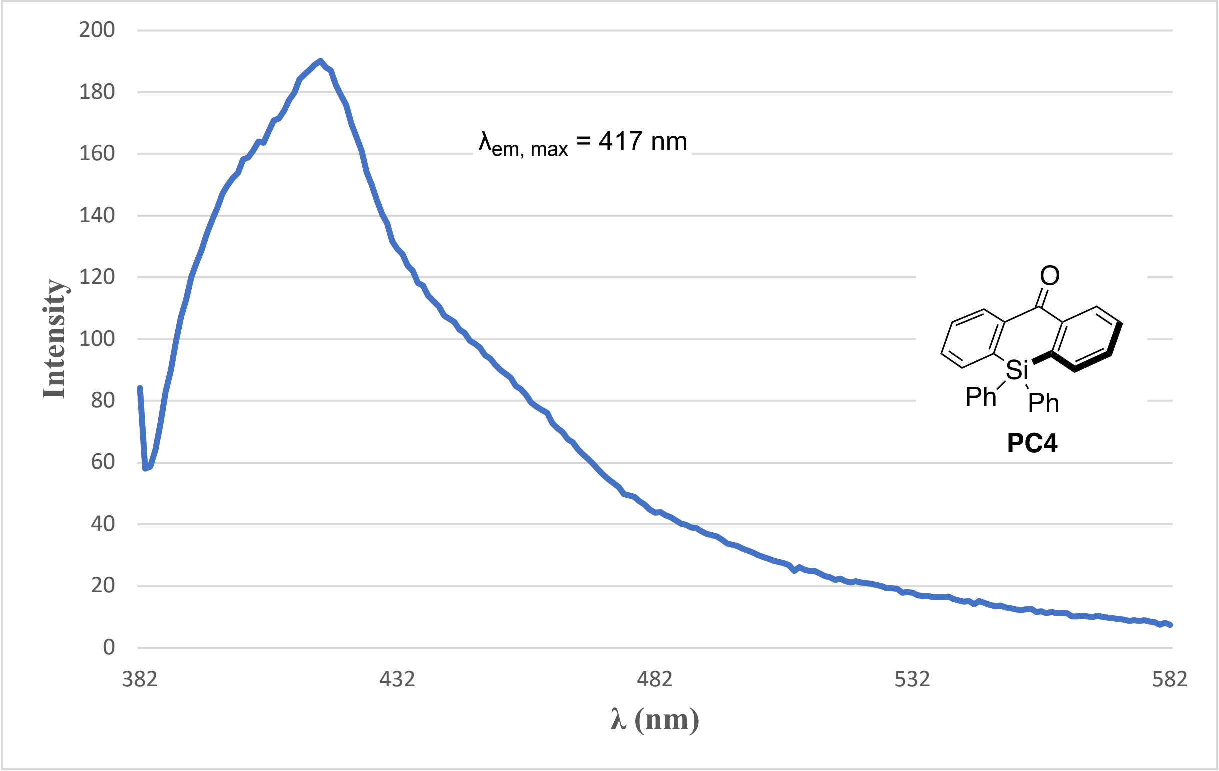

Figure S17. Fluorescence spectrum of PC4 was scanned after excitation at 372 nm.

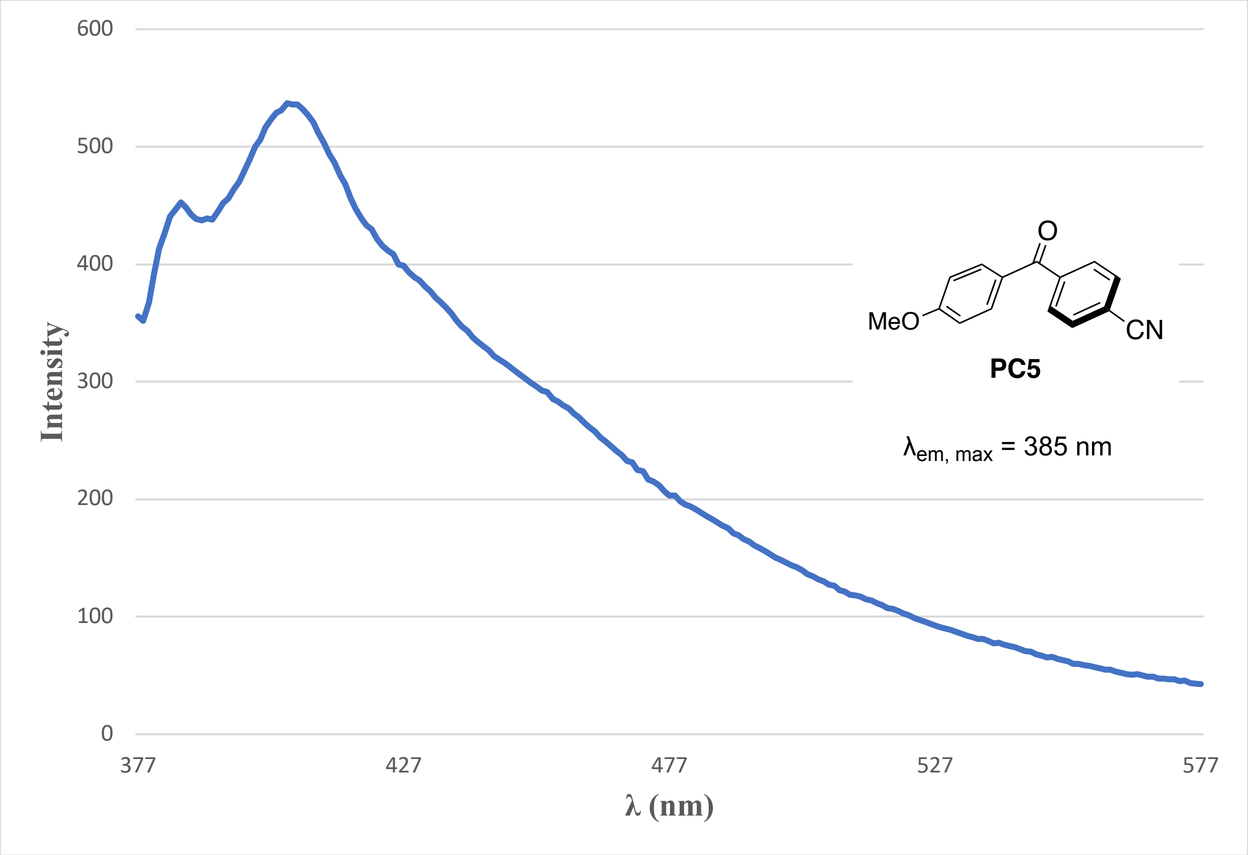

Figure S18. Fluorescence spectrum of **PC5** was scanned after excitation at 367 nm.

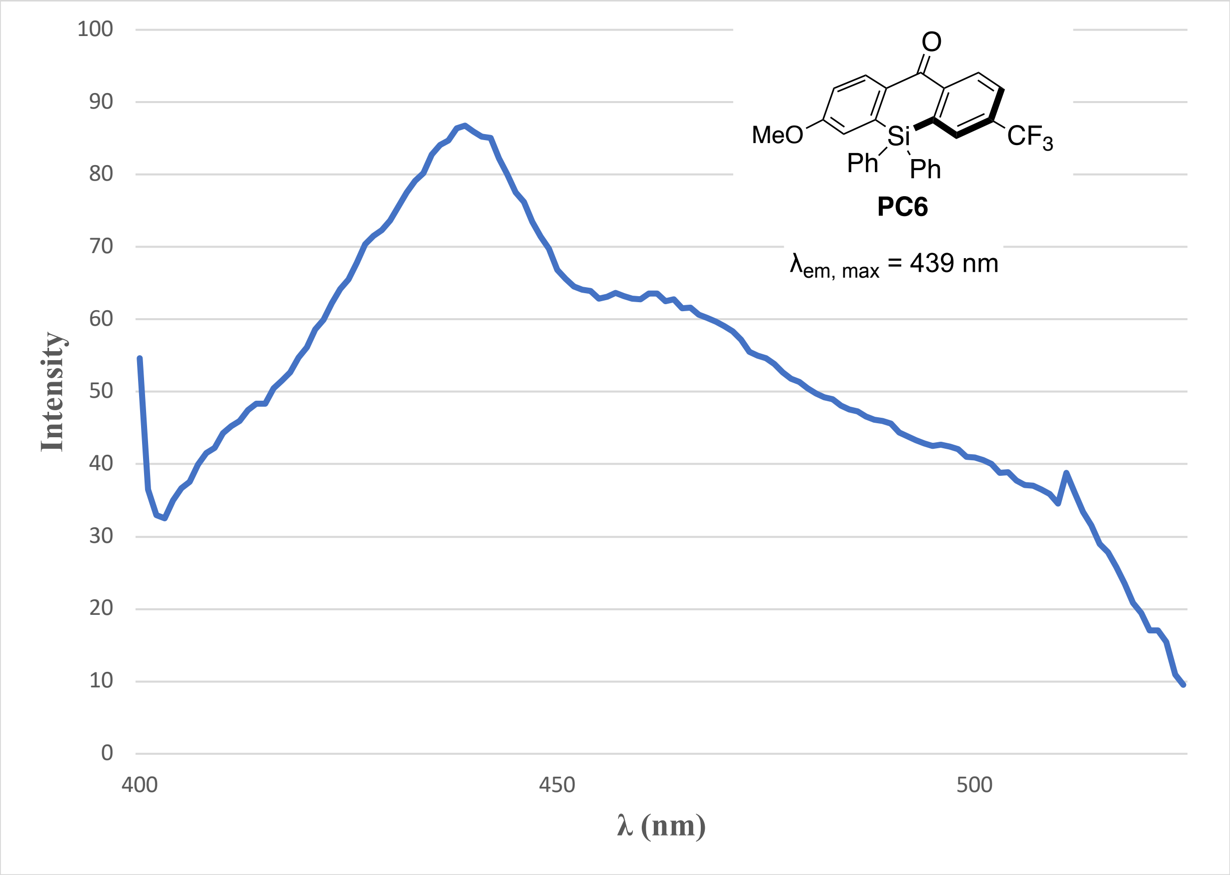

Figure S19. Fluorescence spectrum of **PC6** was scanned after excitation at 390 nm.

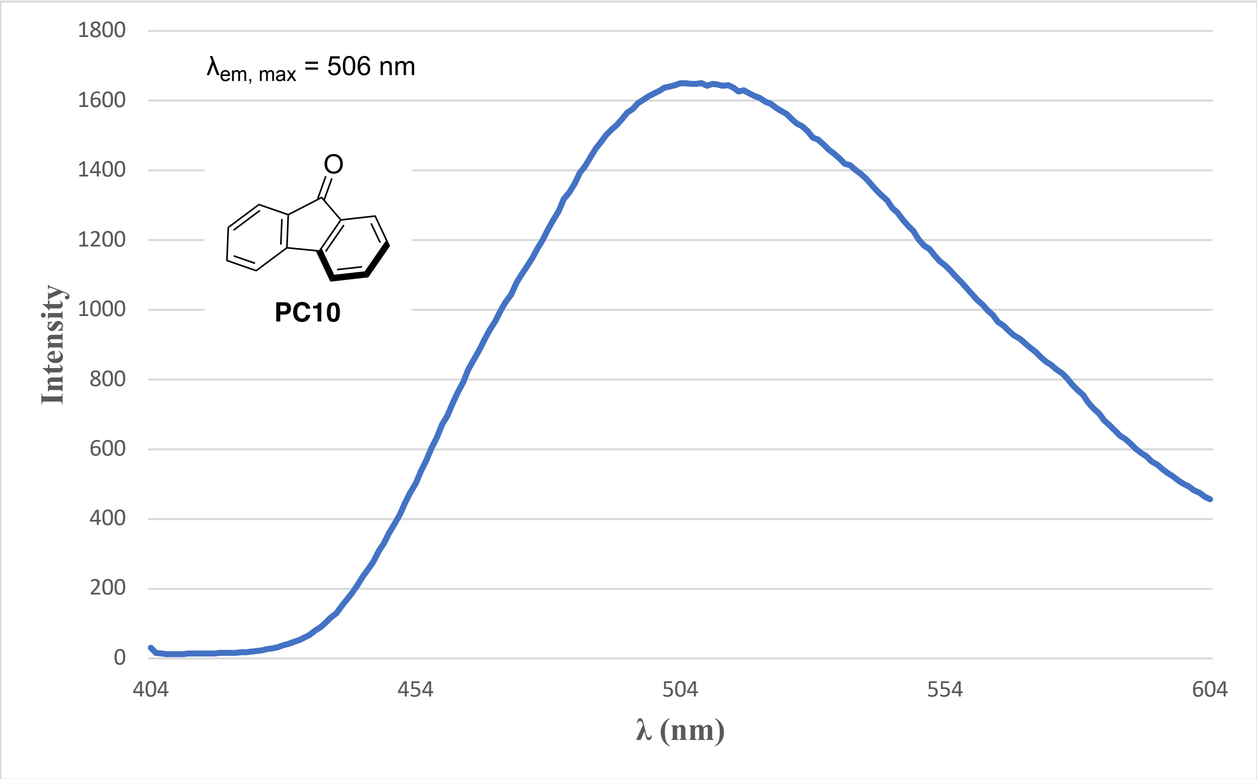

Figure S20. Fluorescence spectrum of **PC10** was scanned after excitation at 394 nm.

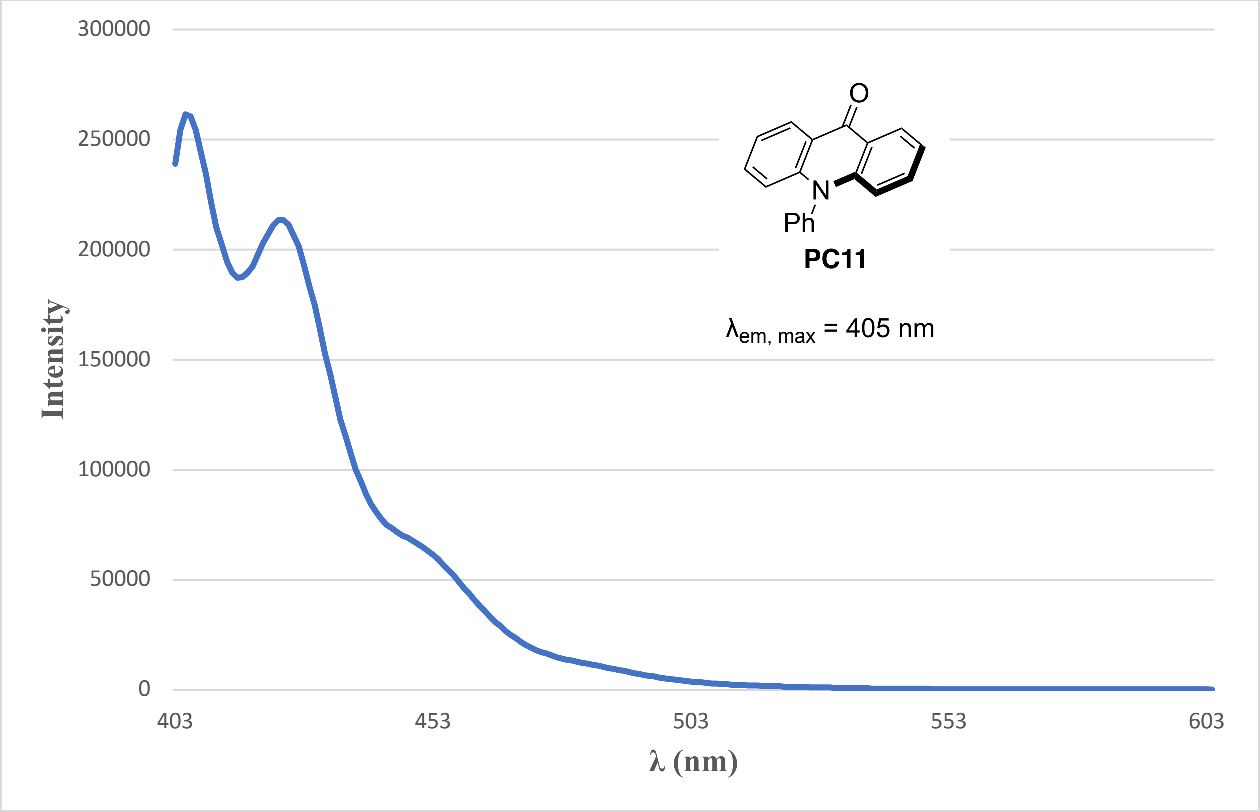

Figure S21. Fluorescence spectrum of **PC11** was scanned after excitation at 393 nm.

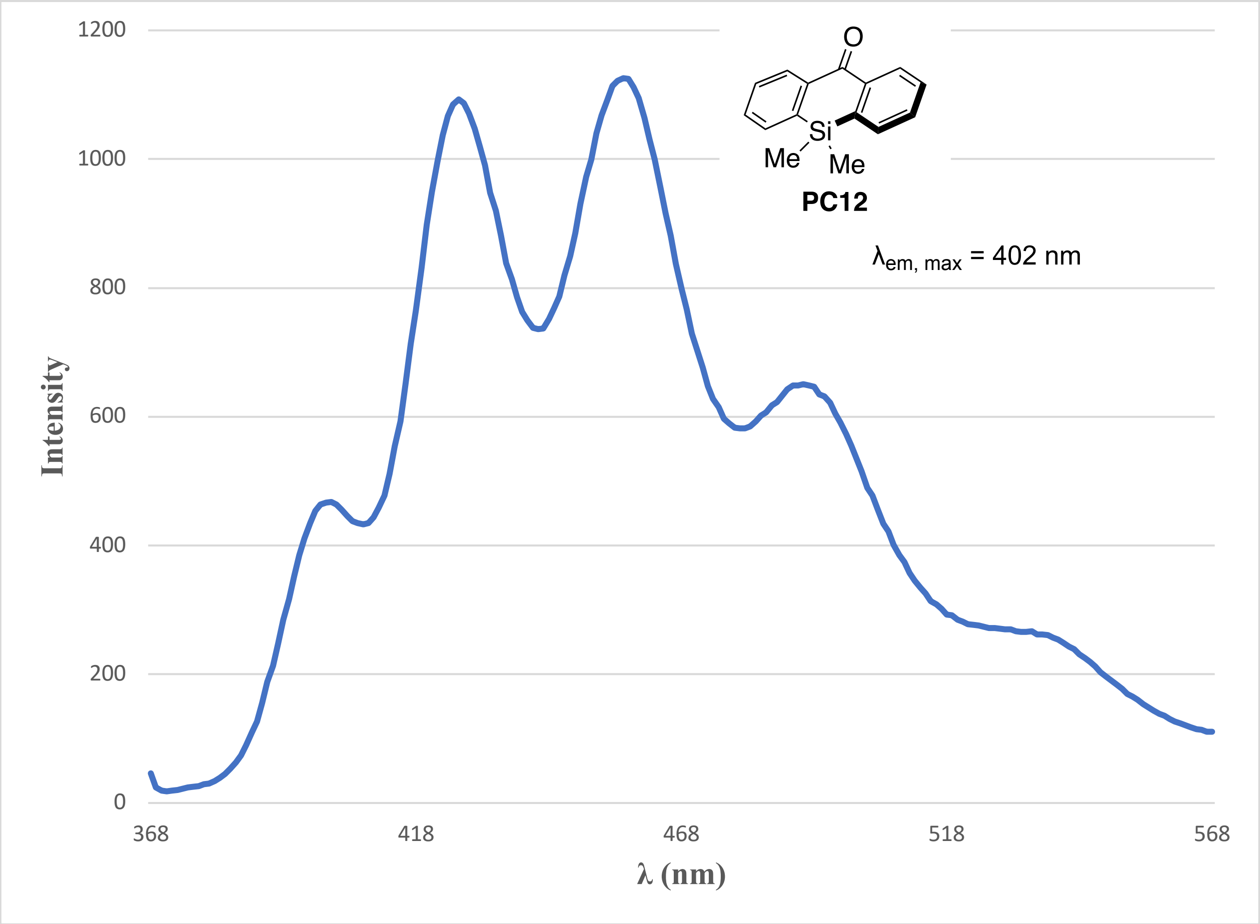

Figure S22. Fluorescence spectrum of **PC12** was scanned after excitation at 358 nm.

15. Cyclic Voltammetry (CV) of Photocatalysts

CV measurements were carried out under nitrogen atmosphere in acetonitrile solutions with 0.1 M of tetrabutylammonium perchlorate (Bu_4NClO_4) as a supporting electrolyte. Measurements were made with a glassy carbon electrode (area = 0.07 cm^2), an $Ag/AgNO_3$ reference electrode, and a Pt wire counter electrode. The concentration of the sample solution was fixed at 10 mM and the sweep rates were set to 100 mV/s. The ferrocenium/ferrocene (Fc^+/Fc) couple was used for calibration.

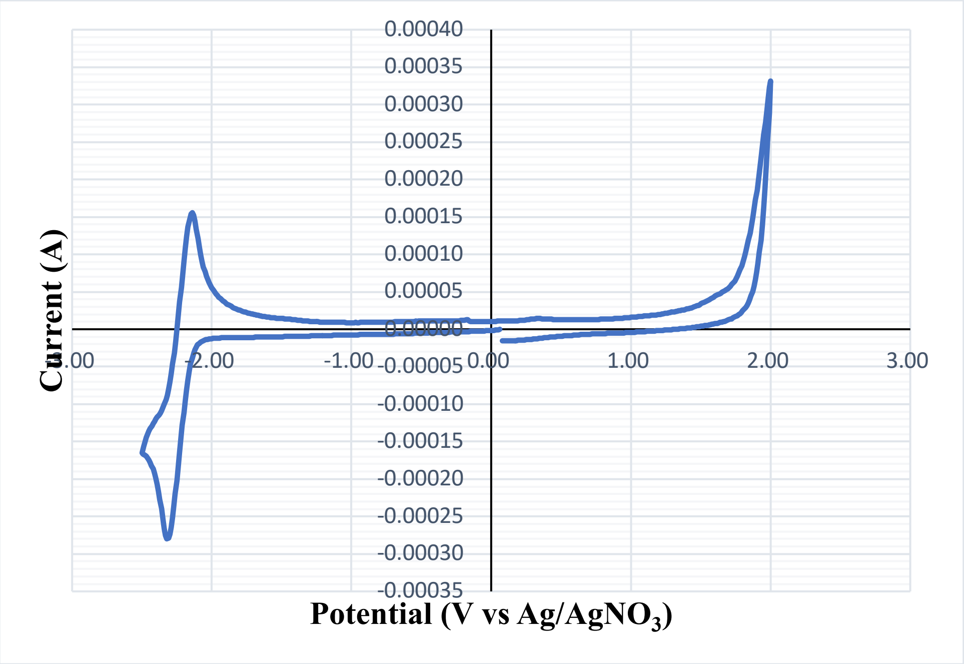

Figure S23. Cyclic voltammogram of **PC1**.

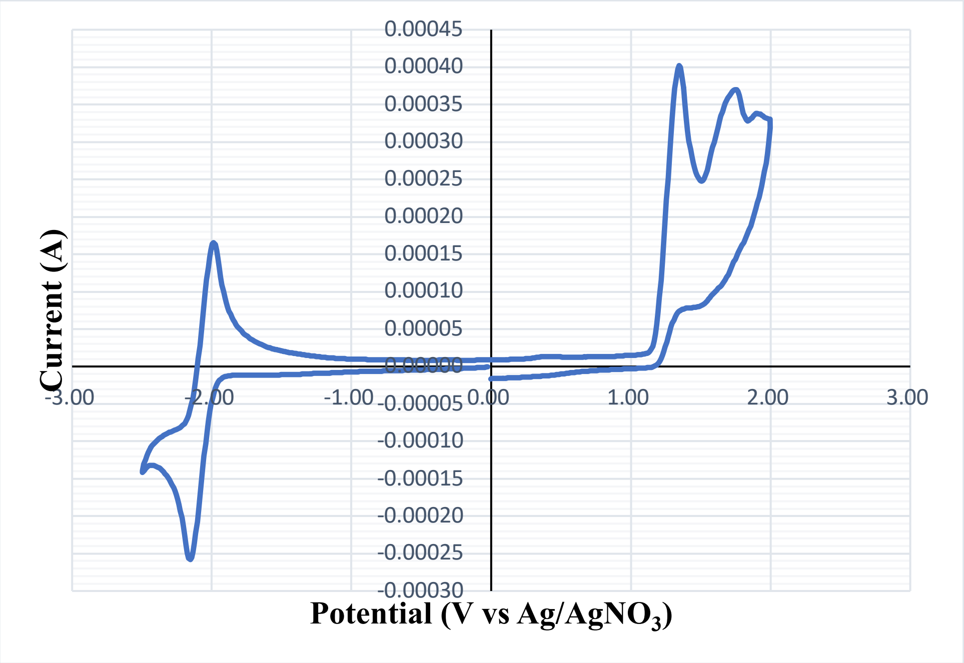

Figure S24. Cyclic voltammogram of **PC2**.

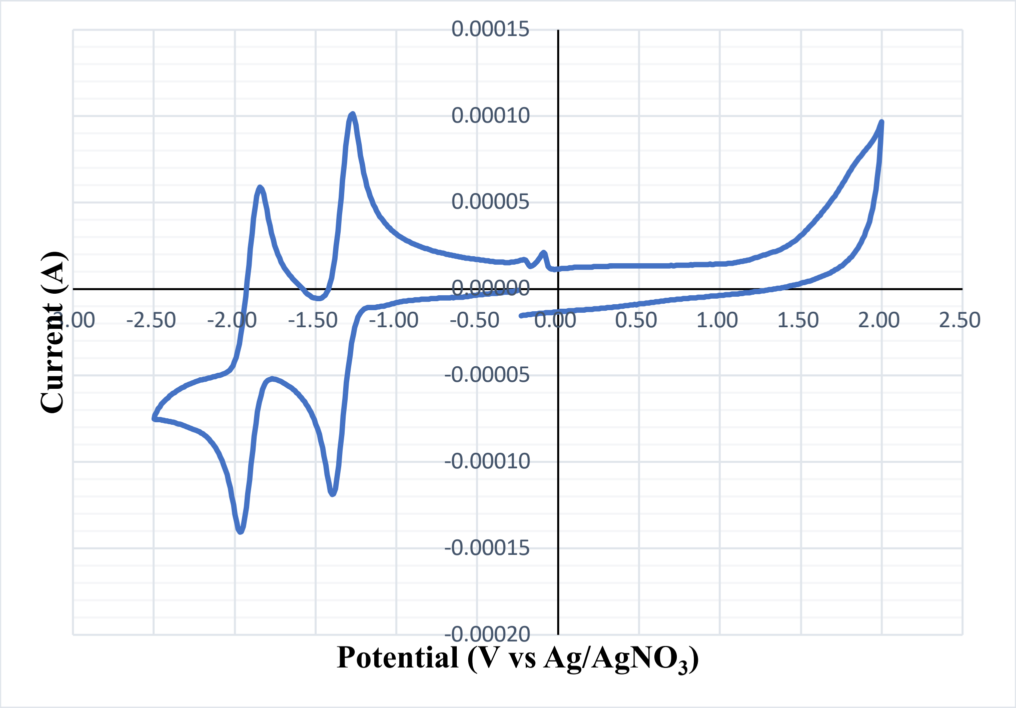

Figure S25. Cyclic voltammogram of **PC3**.

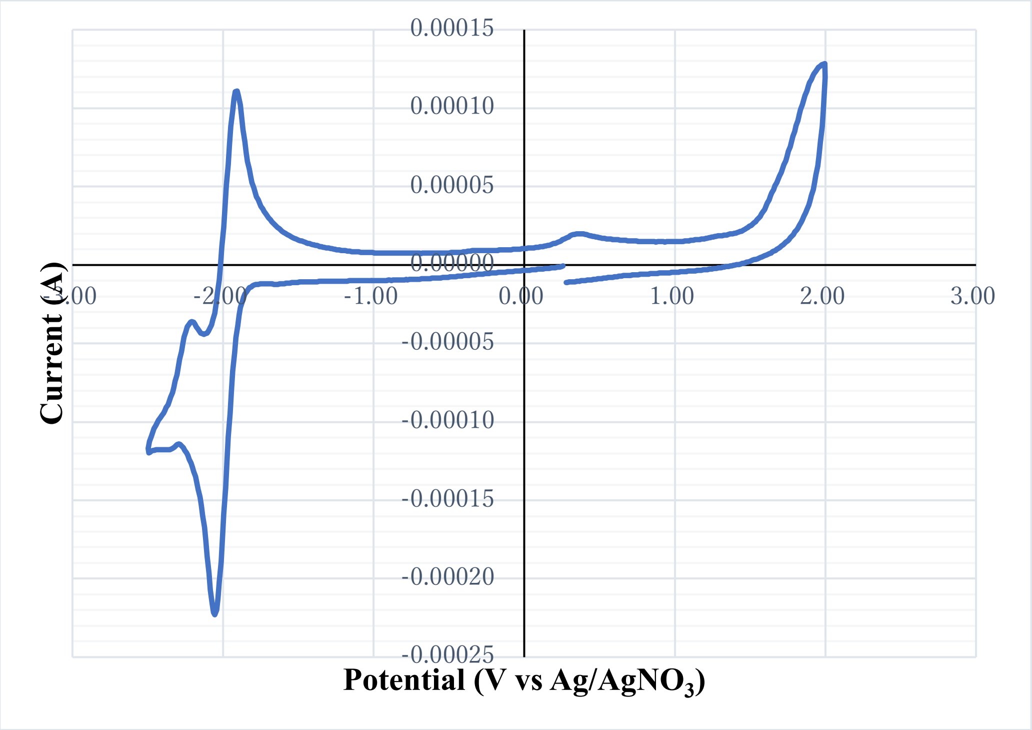

Figure S26. Cyclic voltammogram of **PC4**.

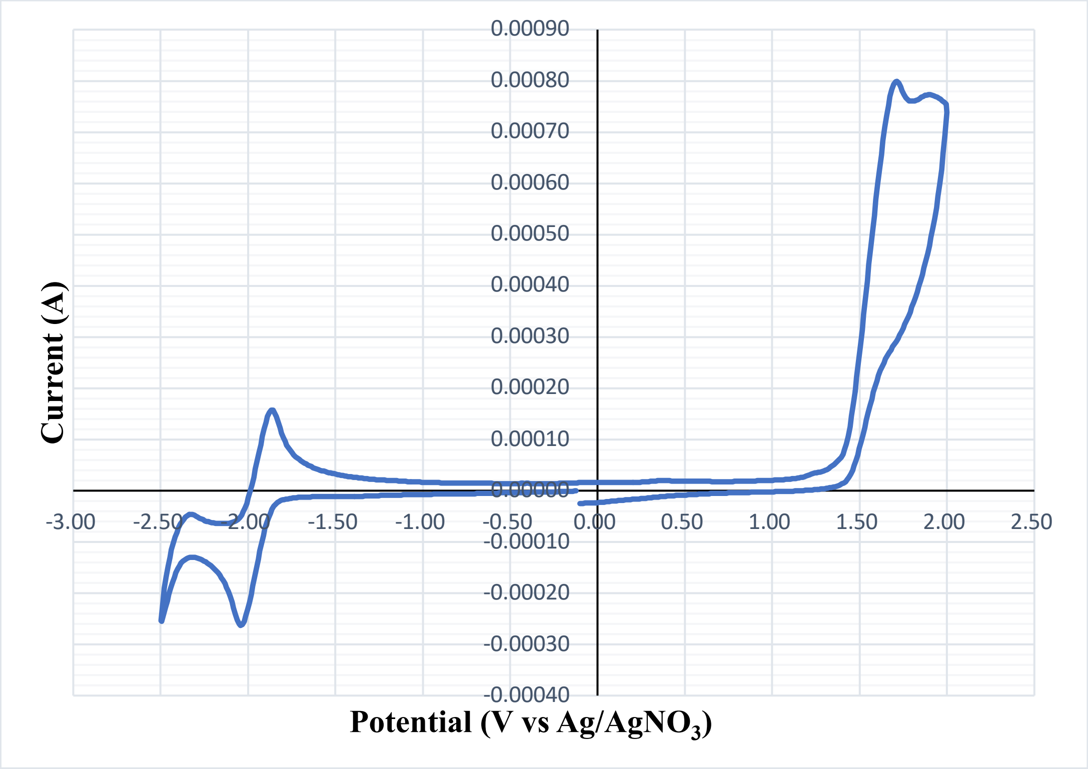

Figure S27. Cyclic voltammogram of **PC5**.

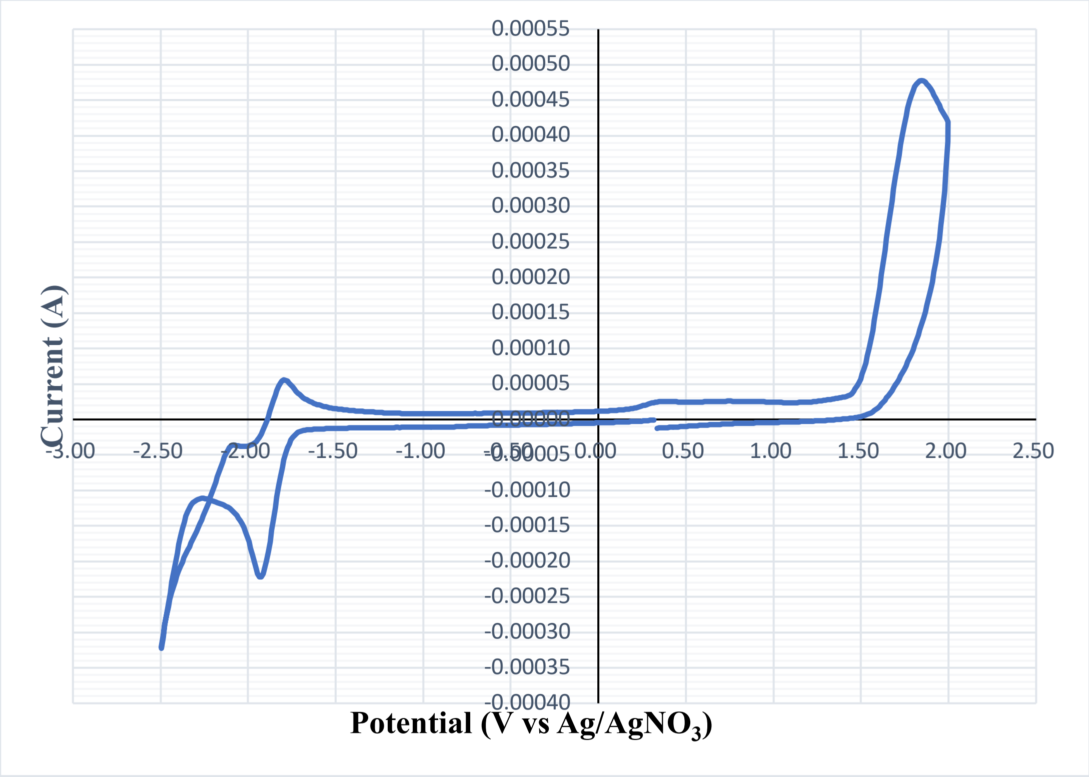

Figure S28. Cyclic voltammogram of **PC6**.

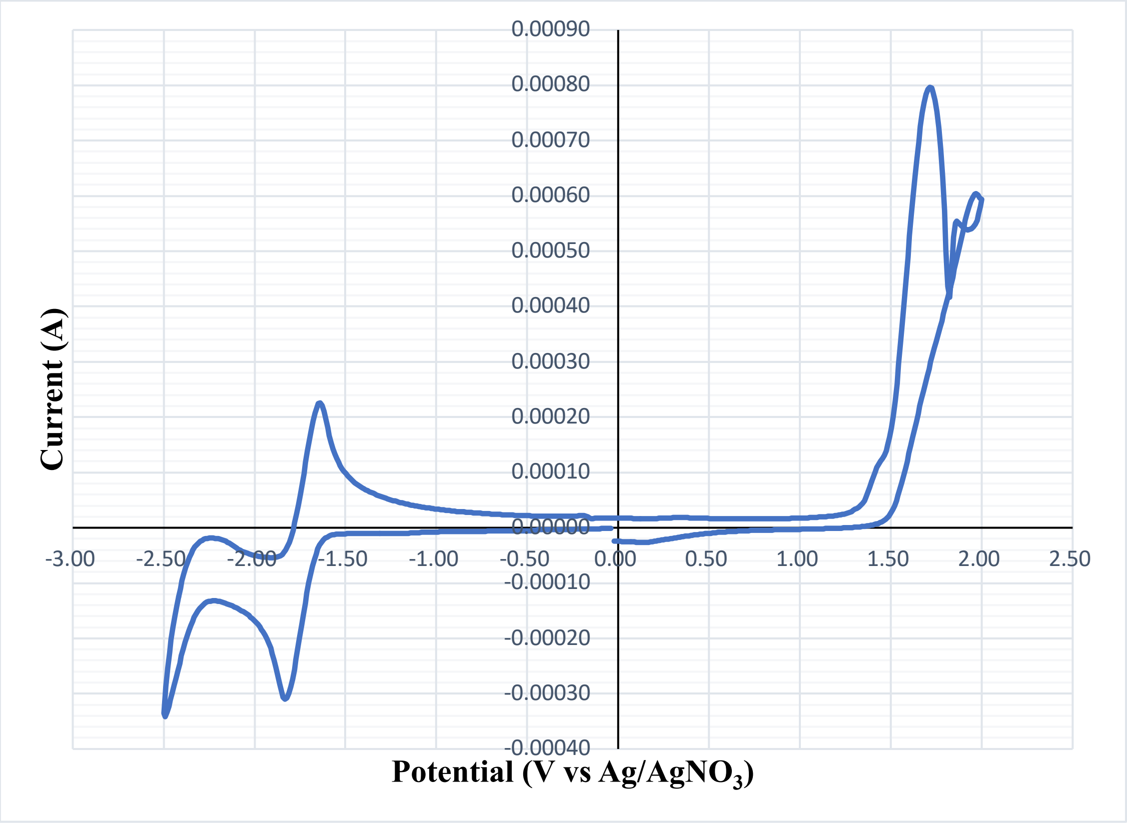

Figure S29. Cyclic voltammogram of **PC10**.

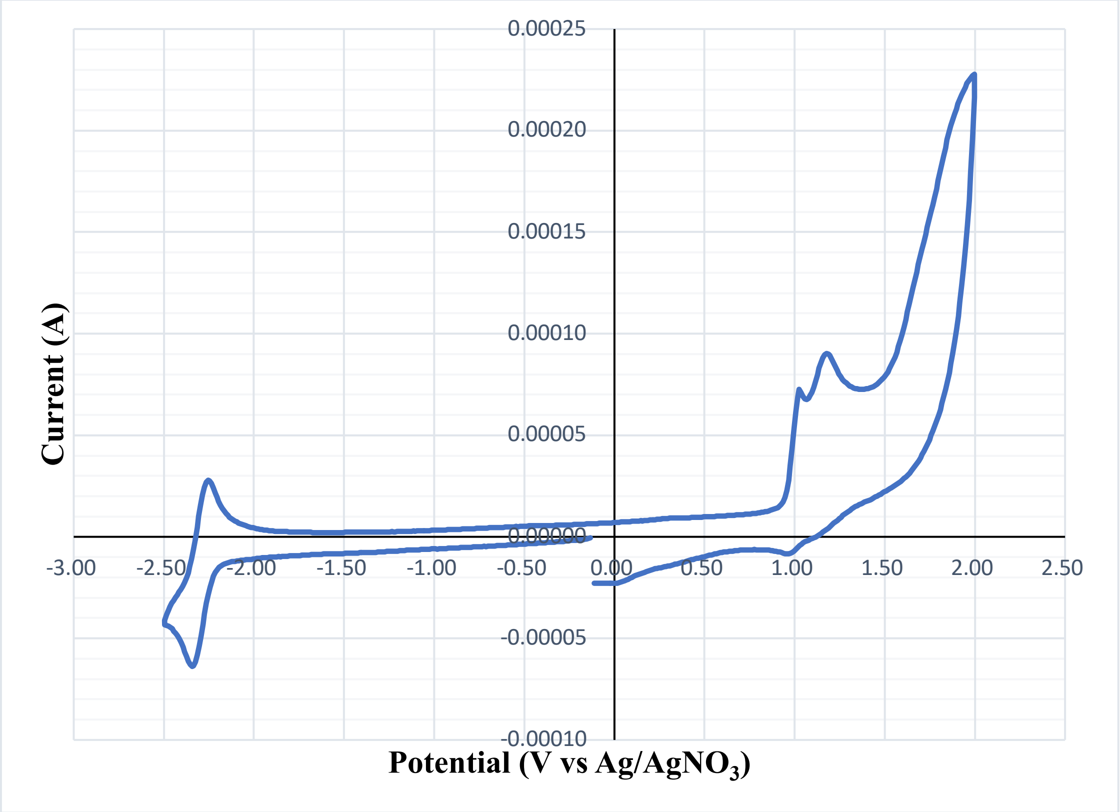

Figure S30. Cyclic voltammogram of **PC11**. The concentration of the sample solution was 2 mM.

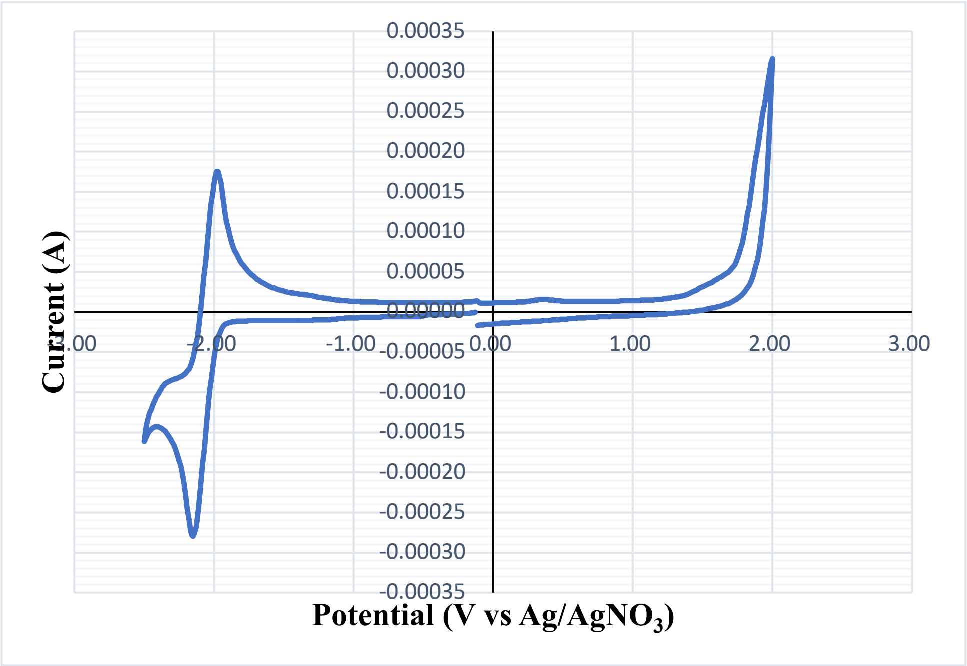

Figure S31. Cyclic voltammogram of **PC12**.

16. Summary of Redox Properties of Organophotocatalysts

	$E_{1/2}$ (PC/PC$^{\bullet -}$) (V vs Fc$^+$/Fc)[a]	$E_{1/2}$ (PC/PC$^{\bullet -}$) (V vs SCE)[b]	$\lambda_{max, em}$ (nm)	$E_{S1, exp}$ (eV)[c]	$E^{0*}_{S1, exp}$ (PC*/PC$^{\bullet -}$) (V vs SCE)[d]
**PC1**	-2.22	-1.79	337	3.32	1.53
**PC2**	-2.06	-1.63	378	3.06	1.43
**PC3**	-1.33	-0.90	410	2.88	1.98
**PC4**	-1.97	-1.54	372	2.97	1.43
**PC5**	-1.90	-1.47	367	3.22	1.75
**PC6**	-1.85	-1.42	390	2.82	1.40
**PC10**	-1.73	-1.30	394	2.45	1.15
**PC11**	-2.29	-1.86	393	3.06	1.20
**PC12**	-2.05	-1.62	358	3.08	1.46

[a] Determined by CV experiments. [b] $E_{1/2}$ (Fc^+/Fc) (V vs SCE) = 0.43 V. [c] Singlet energies were calculated using the maximum wavelength of emission. [d] Singlet excited state reduction potentials were calculated using the singlet energies and the $E_{1/2}$.

17. X-ray Diffraction Analysis for PC6

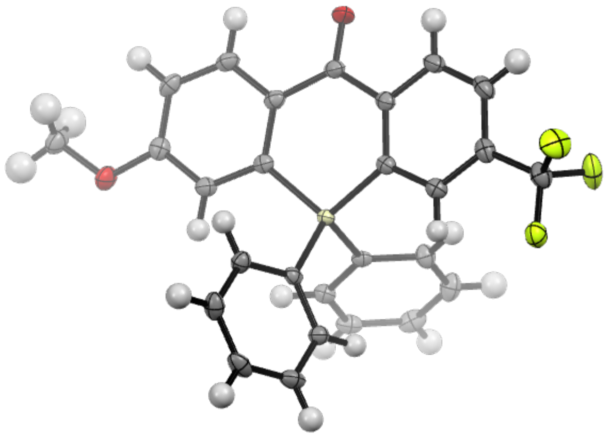

Figure S32. ORTEP diagram of **PC6**. Ellipsoids are drawn at 50% probability. A single crystal used for the analysis was obtained by recrystallization from *n*-hexane and tetrahydrofuran.

Table S9. Crystal data and structure refinement for **PC6**

Bond precision:	C-C = 0.0027 A	Wavelength=0.71073	
Cell:	a=8.589(3)	b=9.902(4)	c=14.679(6)
	alpha=106.882(5)	beta=94.204(3)	gamma=111.756(3)
Temperature:	86 K		
	Calculated	Reported	
Volume	1086.3(7)	1086.2(7)	
Space group	P -1	P -1	
Hall group	-P 1	-P 1	
Moiety formula	C27 H19 F3 O2, Si	?	
Sum formula	C27 H19 F3 O2, Si	C27 H19 F3 O2, Si	
Mr	460.51	460.51	
Dx,g cm-3	1.408	1.408	
Z	2	2	
Mu (mm-1)	0.157	0.157	
F000	476.0	476.0	
F000'	476.43		
h,k,lmax		10,11,17	
Nref		3814	
Tmin,Tmax	0.956,0.981	0.906,0.981	
Tmin'	0.904		

Correction method= # Reported T Limits: Tmin=0.906 Tmax=0.981

Data completeness= Theta(max)= 25.018

R(reflections)= 0.0348(3468) wR2(reflections)= 0.0945(3814)

S = 1.036 Npar= 299

18. Procedure for Radical Trap Experiment

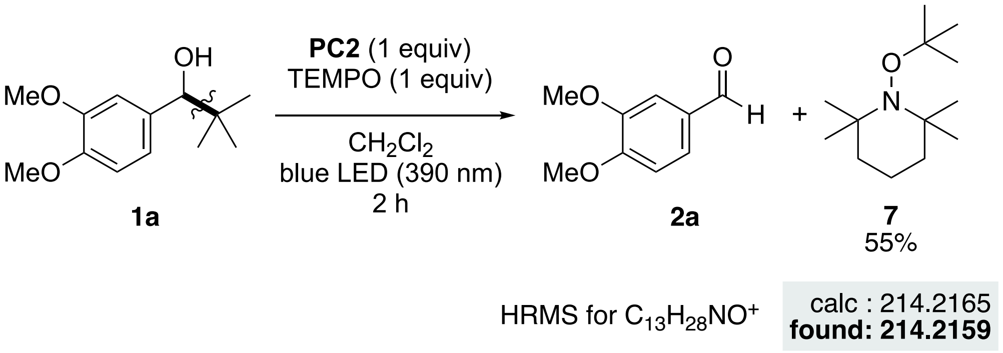

In a glovebox, to a vial equipped with a stirring bar was charged with photoredox catalyst **PC6** (21.2 mg, 0.1 mmol), benzyl alcohol **1a** (22.4 mg, 0.1 mmol), TEMPO (15.6 mg, 0.1 mmol) and CH_2Cl_2 (1.0 mL). After sealing the vial with a cap and removing it from the glovebox, the reaction was stirred and irradiated with a 390 nm blue LED placed 0.5 cm away with a cooling fan to keep the temperature below 50 °C (**Figure S4**). After 2 h, the solvent was removed under reduced pressure, the residue was dissolved in $CDCl_3$ (500 µL) and added 1,1,2,2-tetrachloroethane (18.2 mg, 10.8 µmol) as an internal standard. ^{1}H NMR (400 MHz) yield of **2a** (96%) and yield of **7** (55%) was obtained by comparing the relative value of integration for the singlet peak observed at 9.86 ppm of **2a** and the singlet peak observed at 1.27 ppm of **7** with that of 1,1,2,2-tetrachloroethane observed at 5.96 ppm.

19. Stern–Volmer Experiment

In a typical experiment, the fluoresence intensity was measured using a screw-top quartz cuvette (10 mm light path). The sample was prepared by 120 µL of 5 mM solution of **PC2** in degassed MeCN with degassed MeCN solution of **1a** or TRIP thiol in each concentration (0, 600, 1400, or 1800 µM, respectively) and added until the final volume (3.0 mL). The fluoresence intensity was measured excited absorption maximum (lmax, **PC2** = 378 nm) and the intensity value was determined by the corresponding value of emission maximum (Emax, **PC2** = 405 nm).

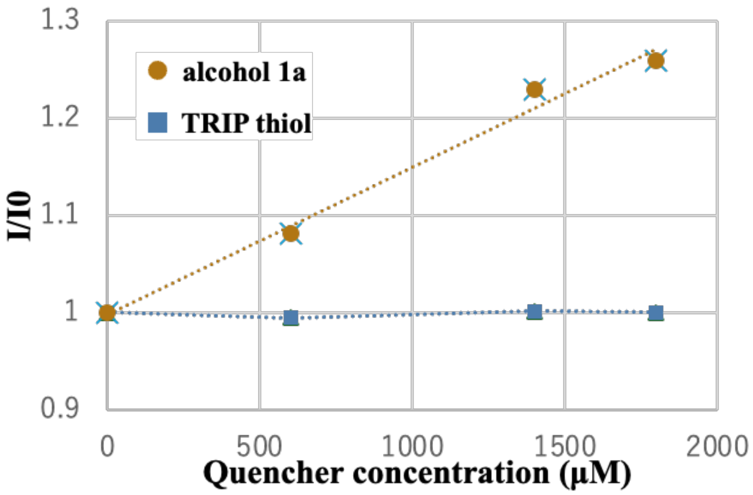

20. EDA Complex

MeCN solution of **PC6** (10 mM) and alcohol **1a** (0.1 M) (red line), MeCN solution of **PC6** (10 mM), and MeCN solution of alcohol **1a** (0.1 M) (red line) in a screw-top 1.0 cm quartz cuvette in glovebox were prepared and the UV-Vis absorption spectra were measured.

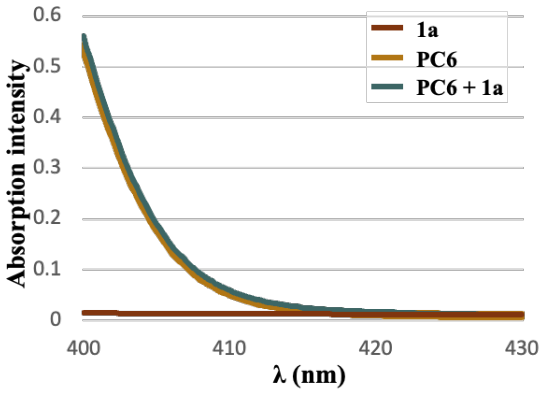

· NMR experiment

The equimolar of substrate **1a** and **PC6** (0.02 M in $CDCl_3$) were mixed and ^1H NMR measurements were performed. Comparison with the ^1H NMR spectra of **1a** and **PC6**, respectively, showed no change in shift values.

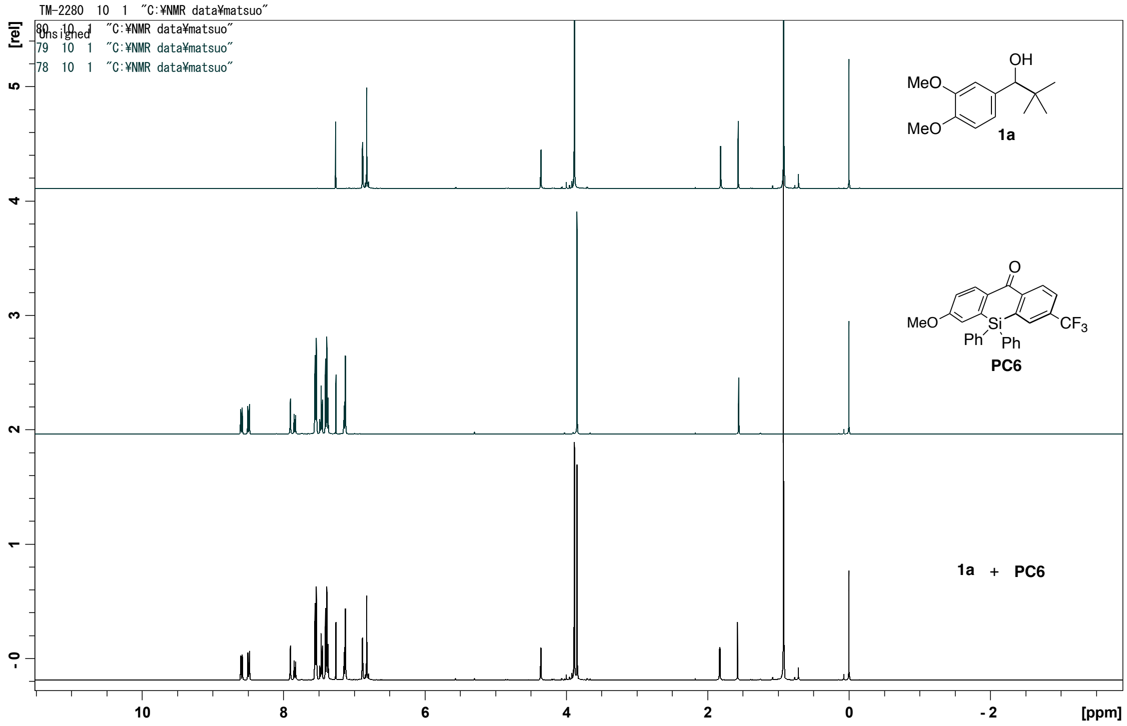

21. Computational Studies

Computational Method. The calculations were performed with Gaussian 16 packages (revision C.01).[22] The DFT method was employed using the (U)M06-2X level[23] with the 6-311+G(d,p) for triplet state of **PC6** and TS calculations. The IRC calculations were performed for each transitions state to confirm the transition state connecting the reaction pathway between the starting materials and the products.

HOMO/LUMO of PC6

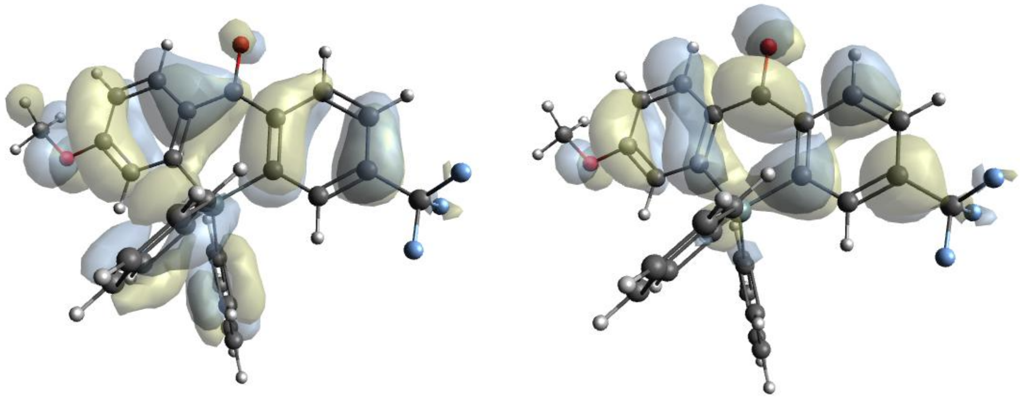

HOMO
(–8.36 eV)

LUMO
(–5.71 eV)

Figure S33. Kohn–Sham orbitals of **PC6** in the triplet states, calculated at the UM062X/6-311++G(dp) level. Orbital energies are shown in parentheses.

Cartesian coordinates of PC6

Center Number	Atomic Number	Atomic Type	Coordinates (Angstroms) X	Y	Z
1	6	0	-0.895252	-2.124924	-0.048277
2	6	0	-1.079879	-0.718476	-0.093477
3	6	0	-2.377150	-0.219028	-0.195349
4	6	0	-3.478727	-1.065429	-0.231903
5	6	0	-3.298927	-2.450785	-0.169575
6	6	0	-2.029486	-2.976145	-0.079944
7	6	0	0.411127	-2.719106	0.017935
8	6	0	1.706749	-2.081240	-0.075613
9	6	0	1.851993	-0.670626	-0.123110
10	6	0	2.858975	-2.886233	-0.140823
11	6	0	4.122222	-2.330162	-0.260661
12	6	0	4.262379	-0.941675	-0.322602

13	6	0	3.123830	-0.132961	-0.252739
14	8	0	0.437061	-4.043597	0.115197
15	1	0	-2.536031	0.854598	-0.237269
16	1	0	-4.160242	-3.108340	-0.190930
17	1	0	-1.897369	-4.051131	-0.039833
18	1	0	2.764773	-3.965896	-0.107298
19	1	0	4.982633	-2.983671	-0.310115
20	1	0	3.271118	0.941995	-0.303432
21	6	0	0.327729	1.250397	1.770152
22	6	0	1.125321	2.368586	2.050276
23	6	0	-0.452514	0.717054	2.802319
24	6	0	1.145130	2.933393	3.320402
25	6	0	-0.437098	1.280979	4.075127
26	6	0	0.362067	2.388785	4.334776
27	1	0	1.766554	3.798819	3.519279
28	1	0	-1.050494	0.857476	4.861874
29	1	0	-1.083811	-0.145364	2.610386
30	1	0	1.731498	2.811468	1.264716
31	14	0	0.367313	0.452562	0.070686
32	6	0	0.941738	1.558525	-2.498489
33	6	0	0.888423	2.524131	-3.497814
34	6	0	0.257250	3.740709	-3.255968
35	6	0	-0.317306	3.990020	-2.013942
36	6	0	-0.257763	3.023492	-1.014476
37	6	0	0.369966	1.791632	-1.240911
38	1	0	1.437581	0.613165	-2.697800
39	1	0	1.338357	2.328798	-4.464235
40	1	0	0.214280	4.493644	-4.034395
41	1	0	-0.808199	4.937105	-1.822629
42	1	0	-0.699063	3.234733	-0.044784
43	1	0	0.373596	2.829776	5.324780
44	8	0	5.445526	-0.295325	-0.446868
45	6	0	6.622836	-1.075533	-0.505227
46	1	0	7.446082	-0.371217	-0.596497
47	1	0	6.748658	-1.667225	0.406541
48	1	0	6.613170	-1.739352	-1.375070
49	6	0	-4.863475	-0.518656	-0.384281
50	9	0	-5.746011	-1.170364	0.392538
51	9	0	-5.315778	-0.636691	-1.646888
52	9	0	-4.937890	0.781029	-0.067246

· ***β*-scission of alcohol via PCET**

The feasibility of the plausible reaction pathway using computational methods to gain mechanistic insights and energy profiles (Fig. 3d). The triplet state (**T1**) of the silicon-bridged benzophenone had higher energy and formed a stabilized complex with substrate **1a** (0 kcal/mol). The formation of alkoxy and ketyl radicals (**INT1**) via **TS1** likely involves either concerted electron/proton transfer (CEPT) or stepwise electron and proton transfer (ET/PT), though DFT calculations couldn't distinguish between them. **TS2** represents the transition state for forming the *t*Bu radical via C–C bond cleavage of the alkoxy radical. Despite the relatively high **TS2** energy (17.9 kcal/mol), it was reasonable.

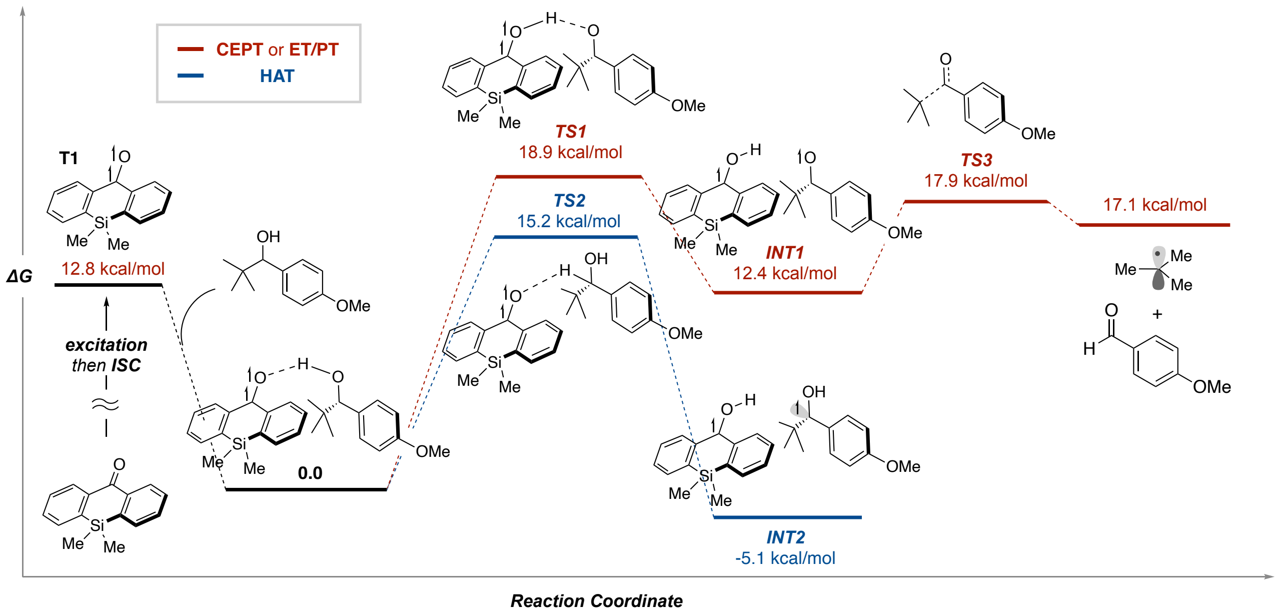

Figure S34. Reaction coordinate for unimolecular PCET and HAT pathway

(U)M06-2X/6-311+G(d,p) (hartree)				
	EE	Thermal Enthalpies	Free Energies	Thermal correction to Gibbs Free Energy
ground state of **PC6**	-944.67102	-944.40806	-944.40901	−0.26201
triplet state of **PC6**	-944.564	-944.30402	-944.30496	−0.25904
benzyl alcohol	-618.45887	-618.1628	-618.16374	−0.29513
**INT0**	-1563.0433	-1562.4855	-1562.4865	−0.5568
**TS1**	-1563.0132	-1562.4631	-1562.4641	−0.54910

**TS2**	-1563.0191	-1562.4657	-1562.4666	−0.55250
SiBP radical	-945.24041	-944.96671	-944.96765	−0.27276
Alkoxy radical	-617.78313	-617.50132	-617.50226	−0.28087
**INT1**	-1563.02354	-1562.46803	-1562.46991	−0.55363
Benzyl radical	-617.80362	-617.52054	-617.52148	−0.28214
**INT2**	-1563.04403	-1562.48725	-1562.48913	−0.55490
**TS3**	-1563.0149	-1562.4596	-1562.4605	−0.55440
*t*Bu radical	-157.74471	-157.62014	-157.62109	−0.12362
anisaldehyde	-460.03085	-459.87752	-459.87847	−0.15238

Cartesian coordinates of ground state of PC6

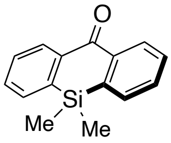

Center Number	Atomic Number	Atomic Type	Coordinates (Angstroms) X	Y	Z
1	6	0	1.327279	-1.000933	0.000077
2	6	0	1.474457	0.395589	-0.000021
3	6	0	2.769191	0.930038	-0.000159
4	6	0	3.890965	0.112263	-0.000235
5	6	0	3.733618	-1.271922	-0.000160
6	6	0	2.462089	-1.821779	-0.000006
7	6	0	0.000000	-1.723380	0.000309
8	6	0	-1.327278	-1.000933	0.000079
9	6	0	-1.474456	0.395588	-0.000015
10	6	0	-2.462088	-1.821779	-0.000005
11	6	0	-3.733618	-1.271923	-0.000156
12	6	0	-3.890964	0.112262	-0.000226
13	6	0	-2.769191	0.930038	-0.000150

14	8	0	-0.000000	-2.936045	0.000229
15	1	0	2.904588	2.008243	-0.000228
16	1	0	4.883164	0.548627	-0.000358
17	1	0	4.602610	-1.919164	-0.000215
18	1	0	2.321433	-2.895197	0.000074
19	1	0	-2.321432	-2.895197	0.000072
20	1	0	-4.602609	-1.919165	-0.000212
21	1	0	-4.883164	0.548626	-0.000347
22	1	0	-2.904588	2.008242	-0.000215
23	14	0	0.000000	1.545989	0.000071
24	6	0	-0.000006	2.623767	-1.534563
25	1	0	-0.882899	3.268147	-1.557780
26	1	0	0.882886	3.268147	-1.557788
27	1	0	-0.000010	2.012433	-2.439092
28	6	0	0.000004	2.623564	1.534847
29	1	0	-0.882893	3.267936	1.558135
30	1	0	0.000014	2.012135	2.439310
31	1	0	0.882894	3.267947	1.558123

Cartesian coordinates of triplet state of PC6

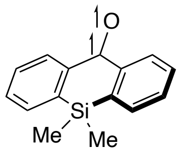

Center Number	Atomic Number	Atomic Type	Coordinates (Angstroms) X	Y	Z
1	6	0	1.305662	-1.004631	0.000035
2	6	0	1.470533	0.403164	-0.000040
3	6	0	2.769637	0.914338	-0.000180
4	6	0	3.890370	0.090082	-0.000216
5	6	0	3.717383	-1.294166	-0.000129
6	6	0	2.447297	-1.838845	-0.000011
7	6	0	-0.000002	-1.622679	0.000133
8	6	0	-1.305659	-1.004625	0.000037
9	6	0	-1.470533	0.403166	-0.000028
10	6	0	-2.447296	-1.838846	-0.000017
11	6	0	-3.717378	-1.294172	-0.000131
12	6	0	-3.890368	0.090081	-0.000213
13	6	0	-2.769641	0.914338	-0.000165
14	8	0	-0.000001	-2.951383	0.000257
15	1	0	2.914062	1.991712	-0.000266
16	1	0	4.885379	0.517982	-0.000314
17	1	0	4.580074	-1.950009	-0.000160
18	1	0	2.325536	-2.916250	0.000038
19	1	0	-2.325526	-2.916250	0.000022
20	1	0	-4.580071	-1.950011	-0.000170
21	1	0	-4.885381	0.517971	-0.000309
22	1	0	-2.914063	1.991712	-0.000243
23	14	0	0.000000	1.559564	0.000122
24	6	0	-0.000012	2.645818	-1.530044
25	1	0	-0.883054	3.290433	-1.550031
26	1	0	0.883024	3.290439	-1.550051
27	1	0	-0.000022	2.038587	-2.437254
28	6	0	0.000009	2.645624	1.530407
29	1	0	-0.883030	3.290249	1.550357
30	1	0	0.000022	2.038457	2.437650
31	1	0	0.883039	3.290264	1.550341

Cartesian coordinates of benzyl alcohol

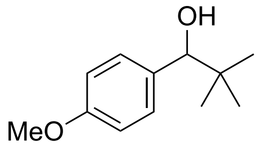

Center Number	Atomic Number	Atomic Type	Coordinates (Angstroms) X	Y	Z
1	6	0	0.493722	1.232066	-0.812129
2	6	0	-0.172831	0.025550	-0.574891
3	6	0	0.570814	-1.051071	-0.104496
4	6	0	1.939043	-0.939515	0.142188
5	6	0	2.580826	0.276010	-0.085866
6	6	0	1.849092	1.364202	-0.568820
7	1	0	-0.062581	2.082101	-1.194991
8	1	0	0.074289	-1.998567	0.073497
9	1	0	2.483078	-1.800126	0.507211
10	8	0	3.905487	0.496580	0.120565
11	6	0	4.680908	-0.576147	0.611280
12	1	0	5.694591	-0.195063	0.711090
13	1	0	4.319839	-0.910495	1.588940
14	1	0	4.676513	-1.419464	-0.086443
15	6	0	-1.662855	-0.096566	-0.822720
16	1	0	-1.941911	0.654194	-1.577168
17	6	0	-2.548270	0.175431	0.418223
18	6	0	-2.279285	1.590552	0.937437
19	1	0	-1.255723	1.698199	1.303416
20	1	0	-2.445653	2.338919	0.155706
21	1	0	-2.959618	1.815242	1.762927
22	6	0	-2.267935	-0.842373	1.527450
23	1	0	-2.400229	-1.862237	1.160101
24	1	0	-1.252532	-0.737973	1.917057
25	1	0	-2.962955	-0.683278	2.356382
26	8	0	-1.999978	-1.393817	-1.298513
27	1	0	-1.433742	-1.595016	-2.048298
28	6	0	-4.015562	0.068062	-0.015646

29	1	0	-4.673126	0.298050	0.826932
30	1	0	-4.236550	0.776845	-0.819746
31	1	0	-4.244265	-0.936324	-0.373384
32	1	0	2.370501	2.295677	-0.751704

Cartesian coordinates of INT0

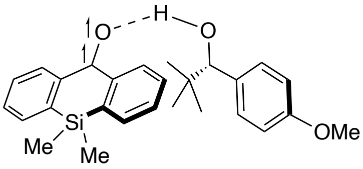

Center Number	Atomic Number	Atomic Type	Coordinates (Angstroms) X	Y	Z
1	6	0	0.594712	1.967472	0.987897
2	6	0	0.198019	2.524184	-0.254621
3	6	0	1.129361	3.285232	-0.962004
4	6	0	2.412004	3.525091	-0.475120
5	6	0	2.776688	3.011004	0.769355
6	6	0	1.884691	2.245832	1.497079
7	6	0	-0.265976	1.072620	1.724706
8	6	0	-1.564343	0.544602	1.372522
9	6	0	-2.252319	0.964591	0.206446
10	6	0	-2.147706	-0.428744	2.213066
11	6	0	-3.376322	-0.981871	1.901095
12	6	0	-4.051062	-0.589312	0.746089
13	6	0	-3.484454	0.377662	-0.079026
14	8	0	0.255344	0.597039	2.857233
15	1	0	0.846330	3.708326	-1.922363
16	1	0	3.114818	4.114357	-1.051416
17	1	0	3.766446	3.199141	1.168285
18	1	0	2.191987	1.836916	2.453133
19	1	0	-1.624004	-0.753057	3.105752
20	1	0	-3.809651	-1.725881	2.560250
21	1	0	-5.007446	-1.030804	0.491853
22	1	0	-4.020092	0.676126	-0.976197
23	14	0	-1.555307	2.322319	-0.876616
24	6	0	0.356160	-0.914490	-1.288825
25	6	0	1.062720	-1.417426	-0.190713
26	6	0	0.472388	-2.428256	0.559187
27	6	0	-0.786300	-2.936488	0.236048
28	6	0	-1.470464	-2.426621	-0.864217
29	6	0	-0.886865	-1.414801	-1.631360
30	1	0	0.792231	-0.108015	-1.872113
31	1	0	1.001563	-2.828123	1.417466
32	1	0	-1.217950	-3.713039	0.853294

33	8	0	-2.702319	-2.839607	-1.267052
34	6	0	-3.329673	-3.848746	-0.506980
35	1	0	-4.295383	-4.025713	-0.976048
36	1	0	-2.745044	-4.774326	-0.518673
37	1	0	-3.479519	-3.524815	0.528120
38	6	0	2.424176	-0.856509	0.168282
39	1	0	2.446141	0.193953	-0.159231
40	6	0	3.619719	-1.559864	-0.519694
41	6	0	3.483620	-1.441224	-2.040011
42	1	0	2.612758	-1.985891	-2.411530
43	1	0	3.393453	-0.394129	-2.347999
44	1	0	4.372633	-1.855958	-2.522535
45	6	0	3.689583	-3.035655	-0.119428
46	1	0	3.744353	-3.138179	0.966332
47	1	0	2.816054	-3.585586	-0.478659
48	1	0	4.580658	-3.496702	-0.554604
49	8	0	2.655155	-0.923205	1.568949
50	1	0	1.868786	-0.606440	2.025704
51	6	0	4.900249	-0.841560	-0.075039
52	1	0	5.768550	-1.272573	-0.581070
53	1	0	4.852027	0.222798	-0.327382
54	1	0	5.042737	-0.930491	1.002358
55	6	0	-1.604033	1.926684	-2.711921
56	1	0	-2.589265	1.552256	-3.003674
57	1	0	-1.414884	2.832164	-3.295236
58	1	0	-0.859119	1.180835	-2.991861
59	6	0	-2.522244	3.906046	-0.585040
60	1	0	-3.560641	3.790717	-0.908402
61	1	0	-2.523531	4.167086	0.475358
62	1	0	-2.083317	4.738264	-1.142182
63	1	0	-1.446790	-1.026207	-2.473775

Cartesian coordinates of TS1

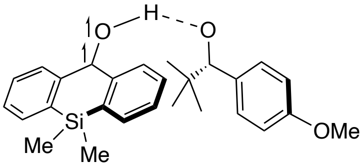

Center Number	Atomic Number	Atomic Type	Coordinates (Angstroms) X	Y	Z
1	6	0	-2.494739	-1.048460	-1.118795
2	6	0	-3.582854	-0.604348	-0.327969
3	6	0	-4.609455	-1.503191	-0.026720
4	6	0	-4.593888	-2.815529	-0.485424
5	6	0	-3.526425	-3.245272	-1.272104
6	6	0	-2.493654	-2.378374	-1.585116
7	6	0	-1.337049	-0.218567	-1.454007
8	6	0	-1.101983	1.113301	-1.010014
9	6	0	-2.003966	1.859851	-0.163000
10	6	0	0.131529	1.750116	-1.410478
11	6	0	0.455890	3.017207	-0.972596
12	6	0	-0.404514	3.712863	-0.122666
13	6	0	-1.622853	3.116733	0.262392
14	8	0	-0.364173	-0.775667	-2.166519
15	1	0	-5.445715	-1.172397	0.584274
16	1	0	-5.401351	-3.493489	-0.235775
17	1	0	-3.500221	-4.264086	-1.641367
18	1	0	-1.664057	-2.713813	-2.192862
19	1	0	0.797290	1.202251	-2.063533
20	1	0	1.389262	3.467650	-1.290909
21	1	0	-0.150253	4.705328	0.228558
22	1	0	-2.286711	3.682148	0.911833
23	14	0	-3.677408	1.151802	0.291701
24	6	0	3.075495	0.917194	0.418655
25	6	0	2.622862	-0.392183	0.203395
26	6	0	3.532933	-1.339932	-0.260284
27	6	0	4.866751	-1.013385	-0.487135
28	6	0	5.301299	0.292144	-0.256945
29	6	0	4.394724	1.258548	0.197984
30	1	0	2.376008	1.675370	0.754728

31	1	0	3.186828	-2.350589	-0.443510
32	1	0	5.547874	-1.776457	-0.838334
33	8	0	6.571858	0.720065	-0.444901
34	6	0	7.521082	-0.212811	-0.921613
35	1	0	8.459110	0.330274	-1.007483
36	1	0	7.643292	-1.043433	-0.220039
37	1	0	7.232513	-0.601299	-1.902789
38	6	0	1.185366	-0.778710	0.450911
39	1	0	0.576371	0.139261	0.216787
40	6	0	0.790324	-1.105674	1.926597
41	6	0	1.152350	0.062824	2.845057
42	1	0	2.231455	0.222963	2.895538
43	1	0	0.676560	0.990683	2.508316
44	1	0	0.794852	-0.146252	3.856747
45	6	0	1.520989	-2.376449	2.370761
46	1	0	1.273513	-3.210013	1.709863
47	1	0	2.604427	-2.230533	2.362982
48	1	0	1.221184	-2.640881	3.388519
49	8	0	0.775805	-1.794937	-0.374984
50	1	0	0.418427	-1.419706	-1.321902
51	6	0	-0.724739	-1.323687	1.960786
52	1	0	-1.044199	-1.570145	2.977287
53	1	0	-1.249653	-0.413038	1.651664
54	1	0	-1.028415	-2.130586	1.291622
55	6	0	-3.970086	1.233430	2.145383
56	1	0	-3.966859	2.269429	2.495655
57	1	0	-4.943581	0.805094	2.399691
58	1	0	-3.203085	0.682844	2.693506
59	6	0	-5.039024	2.106929	-0.580732
60	1	0	-5.056949	3.149927	-0.252393
61	1	0	-4.889779	2.090700	-1.662229
62	1	0	-6.017382	1.668269	-0.365651
63	1	0	4.756831	2.266208	0.360691

Cartesian coordinates of TS2

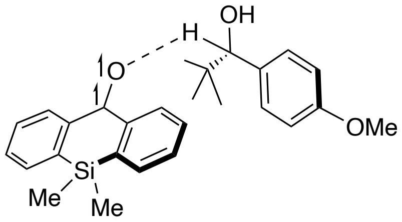

Center Number	Atomic Number	Atomic Type	Coordinates (Angstroms) X	Y	Z
1	6	0	-2.811116	-0.961996	-1.016910
2	6	0	-3.955063	-0.329075	-0.466257
3	6	0	-5.196895	-0.946015	-0.619698
4	6	0	-5.337445	-2.160343	-1.285759
5	6	0	-4.204204	-2.790307	-1.799105
6	6	0	-2.955989	-2.208186	-1.663353
7	6	0	-1.486093	-0.369650	-0.924025
8	6	0	-1.202538	1.035935	-0.705017
9	6	0	-2.126266	1.907027	-0.069939
10	6	0	0.021148	1.562536	-1.176798
11	6	0	0.325620	2.902568	-1.010680
12	6	0	-0.572860	3.759638	-0.375911
13	6	0	-1.788246	3.251768	0.076960
14	8	0	-0.477887	-1.181326	-1.231894
15	1	0	-6.080410	-0.472167	-0.200728
16	1	0	-6.314254	-2.615918	-1.394403
17	1	0	-4.297746	-3.740716	-2.311912
18	1	0	-2.081217	-2.696292	-2.075642
19	1	0	0.713944	0.916817	-1.703833
20	1	0	1.266874	3.284395	-1.391384
21	1	0	-0.333582	4.808097	-0.246093
22	1	0	-2.489976	3.925553	0.560782
23	14	0	-3.719968	1.192674	0.592893
24	6	0	3.033406	0.710583	0.176510
25	6	0	2.897272	-0.680835	0.043695
26	6	0	4.016951	-1.416434	-0.345566
27	6	0	5.247167	-0.804703	-0.562847
28	6	0	5.365824	0.577370	-0.413165
29	6	0	4.245708	1.332151	-0.049072

30	1	0	2.173961	1.316162	0.432498
31	1	0	3.921588	-2.486537	-0.475637
32	1	0	6.095086	-1.411404	-0.850259
33	8	0	6.511355	1.273130	-0.604945
34	6	0	7.667095	0.553538	-0.985055
35	1	0	8.460515	1.290328	-1.084583
36	1	0	7.943661	-0.179518	-0.221333
37	1	0	7.517913	0.046087	-1.942772
38	6	0	1.593314	-1.370331	0.291820
39	1	0	0.768658	-0.823547	-0.336958
40	6	0	1.061458	-1.375369	1.763534
41	6	0	0.784680	0.035923	2.291856
42	1	0	1.704927	0.602829	2.438878
43	1	0	0.123276	0.600716	1.626975
44	1	0	0.291527	-0.041035	3.264660
45	6	0	2.129357	-2.053056	2.635975
46	1	0	2.363696	-3.049647	2.255164
47	1	0	3.048951	-1.463866	2.659250
48	1	0	1.756785	-2.151062	3.659400
49	8	0	1.638016	-2.655499	-0.244520
50	1	0	0.731012	-2.912418	-0.450217
51	6	0	-0.237383	-2.189434	1.838160
52	1	0	-0.601255	-2.188281	2.868670
53	1	0	-1.022286	-1.765588	1.206501
54	1	0	-0.079595	-3.232230	1.553360
55	6	0	-3.471042	0.668937	2.380859
56	1	0	-3.251374	1.532660	3.014366
57	1	0	-4.360117	0.168778	2.773914
58	1	0	-2.630064	-0.026649	2.451188
59	6	0	-5.150304	2.396262	0.456956
60	1	0	-4.973527	3.283781	1.070268
61	1	0	-5.297351	2.716712	-0.576330
62	1	0	-6.078595	1.939372	0.809979
63	1	0	4.355313	2.405703	0.043590

Cartesian coordinates of SiBP radical

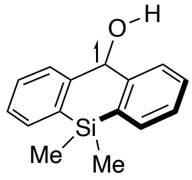

Center Number	Atomic Number	Atomic Type	Coordinates (Angstroms) X	Y	Z
1	6	0	-1.299733	-0.979329	0.013549
2	6	0	-1.468147	0.427229	-0.069606
3	6	0	-2.758530	0.943234	-0.199111
4	6	0	-3.879167	0.121138	-0.245345
5	6	0	-3.710603	-1.259776	-0.148352
6	6	0	-2.446711	-1.805331	-0.017449
7	6	0	-0.003752	-1.606776	0.122667
8	6	0	1.292446	-0.985946	0.001621
9	6	0	1.463097	0.426571	-0.002009
10	6	0	2.447986	-1.795716	-0.125157
11	6	0	3.713232	-1.240949	-0.203898
12	6	0	3.880175	0.141302	-0.173944
13	6	0	2.751624	0.951474	-0.086881
14	8	0	-0.063661	-2.955473	0.289499
15	1	0	-2.896239	2.019677	-0.261150
16	1	0	-4.869403	0.548085	-0.349082
17	1	0	-4.573778	-1.914749	-0.176035
18	1	0	-2.326040	-2.877385	0.055047
19	1	0	2.369293	-2.873444	-0.218593
20	1	0	4.574148	-1.891355	-0.305687
21	1	0	4.869446	0.577892	-0.234043
22	1	0	2.883739	2.030447	-0.086753
23	14	0	-0.007911	1.577835	0.092077
24	6	0	-0.048626	2.477270	1.740101
25	1	0	0.836991	3.107075	1.862201
26	1	0	-0.931286	3.118943	1.810045
27	1	0	-0.078660	1.765893	2.567936
28	6	0	0.040354	2.838231	-1.297847
29	1	0	0.918724	3.483629	-1.211406
30	1	0	0.073111	2.344141	-2.270719

31	1	0	-0.843513	3.481389	-1.270514
32	1	0	0.771648	-3.281890	0.633649

Cartesian coordinates of alkoxy radical

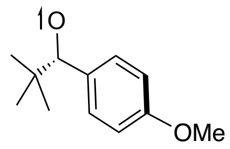

Center Number	Atomic Number	Atomic Type	Coordinates (Angstroms) X	Y	Z
1	6	0	0.473867	1.271995	-0.723247
2	6	0	-0.174116	0.040064	-0.603801
3	6	0	0.576133	-1.068136	-0.234272
4	6	0	1.939099	-0.961789	0.039176
5	6	0	2.565949	0.278667	-0.065655
6	6	0	1.825446	1.397905	-0.454565
7	1	0	-0.089188	2.146587	-1.034843
8	1	0	0.091043	-2.035803	-0.173896
9	1	0	2.493133	-1.846687	0.321627
10	8	0	3.885278	0.495614	0.176407
11	6	0	4.673251	-0.611529	0.559041
12	1	0	5.681184	-0.229285	0.702965
13	1	0	4.312733	-1.048967	1.495370
14	1	0	4.682945	-1.378834	-0.221374
15	6	0	-1.658362	-0.081472	-0.885414
16	1	0	-1.981980	0.724412	-1.565679
17	6	0	-2.581879	0.117985	0.405126
18	6	0	-2.270052	1.483849	1.017014
19	1	0	-1.245281	1.537540	1.388236
20	1	0	-2.421965	2.290328	0.293731
21	1	0	-2.946650	1.656556	1.858667
22	6	0	-2.311628	-0.985221	1.429317
23	1	0	-2.520144	-1.973258	1.013247
24	1	0	-1.275414	-0.962422	1.773285
25	1	0	-2.958086	-0.836124	2.298600

26	8	0	-2.009600	-1.291752	-1.397575
27	6	0	-4.044392	0.077104	-0.045455
28	1	0	-4.696103	0.278549	0.808952
29	1	0	-4.241111	0.837969	-0.805971
30	1	0	-4.307137	-0.896156	-0.460522
31	1	0	2.336710	2.348161	-0.546643

Cartesian coordinates of INT1

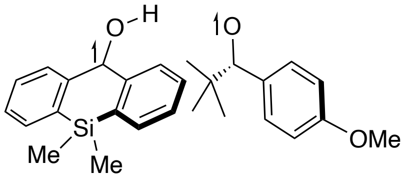

Center Number	Atomic Number	Atomic Type	Coordinates (Angstroms) X	Y	Z
1	6	0	-0.396275	2.058141	-0.291391
2	6	0	-0.023438	0.346574	1.397474
3	6	0	-1.172740	-0.319808	0.999607
4	6	0	-1.934739	0.209559	-0.057325
5	6	0	-1.537161	1.404619	-0.700710
6	1	0	-0.092445	2.978383	-0.780136
7	1	0	0.574786	-0.058953	2.208280
8	1	0	-1.466683	-1.237212	1.495237
9	1	0	-2.152781	1.784378	-1.509941
10	6	0	-3.515774	-1.566370	0.063798
11	1	0	-4.421979	-1.832462	-0.478069
12	1	0	-2.770134	-2.359185	-0.049211
13	1	0	-3.748527	-1.414657	1.122085
14	8	0	-3.054713	-0.353042	-0.529724
15	6	0	1.639546	-0.452973	-1.056370
16	6	0	3.074486	-1.257219	0.208676
17	7	0	2.348118	0.607186	-0.508106
18	1	0	0.821020	-0.289382	-1.745708
19	1	0	3.703766	-1.965049	0.731763
20	6	0	0.386764	1.534874	0.762179
21	1	0	1.226300	2.098439	1.150222

22	7	0	2.088434	-1.624289	-0.658251
23	7	0	3.226360	0.070769	0.328877

Cartesian coordinates of benzyl radical

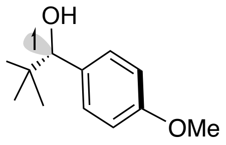

Center Number	Atomic Number	Atomic Type	Coordinates (Angstroms) X	Y	Z
1	6	0	0.428322	1.137731	-0.000293
2	6	0	-0.097589	-0.185458	-0.000138
3	6	0	0.866784	-1.228238	0.000008
4	6	0	2.231308	-0.982575	0.000058
5	6	0	2.707277	0.327705	-0.000058
6	6	0	1.784262	1.378782	-0.000246
7	1	0	-0.220516	1.996244	-0.000509
8	1	0	0.525169	-2.253102	0.000111
9	1	0	2.910822	-1.824770	0.000188
10	8	0	4.021504	0.680301	-0.000034
11	6	0	4.973663	-0.360260	0.000269
12	1	0	5.949189	0.120977	0.000309
13	1	0	4.876480	-0.985731	0.893508
14	1	0	4.876716	-0.986044	-0.892775
15	6	0	-1.477747	-0.545473	-0.000205
16	6	0	-2.768990	0.256065	0.000090
17	6	0	-2.595877	1.776800	0.000426
18	1	0	-2.070952	2.124355	0.891485
19	1	0	-2.071509	2.124835	-0.890775
20	1	0	-3.587724	2.235634	0.000840
21	6	0	-3.586746	-0.110662	1.259519
22	1	0	-3.834310	-1.174368	1.313896
23	1	0	-3.030637	0.147577	2.163187
24	1	0	-4.530631	0.440673	1.261508
25	8	0	-1.673163	-1.904977	-0.000395
26	1	0	-2.611272	-2.104036	-0.000319

27	6	0	-3.587053	-0.110143	-1.259276
28	1	0	-4.530916	0.441235	-1.260833
29	1	0	-3.031144	0.148413	-2.162977
30	1	0	-3.834664	-1.173820	-1.313964
31	1	0	2.163487	2.393694	-0.000380

Cartesian coordinates of INT2

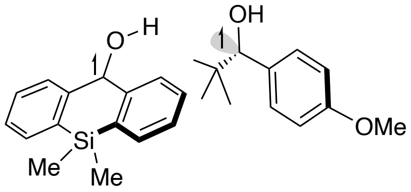

Center Number	Atomic Number	Atomic Type	Coordinates (Angstroms) X	Y	Z
1	8	0	0.063382	-1.504391	-1.196842
2	6	0	-0.550059	0.491545	-0.141887
3	7	0	-1.353205	1.570778	0.085744
4	6	0	-0.555355	2.634535	0.390794
5	7	0	0.703683	2.320148	0.392649
6	7	0	0.709335	0.979892	0.063264
7	6	0	-2.174530	-1.425112	-0.362442
8	6	0	-2.558252	-2.568982	-1.086095
9	6	0	-3.788635	-3.149038	-0.817688
10	6	0	-4.607162	-2.583933	0.163502
11	6	0	-4.139457	-1.455494	0.834038
12	1	0	-4.742802	-0.990707	1.611556
13	1	0	-5.575444	-3.006060	0.409384
14	1	0	-4.111002	-4.030150	-1.365128
15	1	0	-1.885661	-2.968606	-1.836597
16	6	0	-2.762519	1.943762	-0.080235
17	1	0	-3.172603	1.476665	-0.977154
18	1	0	-3.326601	1.602441	0.788841
19	6	0	-0.843726	-0.837704	-0.609837
20	7	0	-2.955799	-0.886766	0.592943
21	6	0	1.949212	0.265404	0.104543

22	6	0	2.904027	0.531021	-0.885421
23	6	0	2.175482	-0.638749	1.145247
24	6	0	4.118572	-0.149231	-0.813326
25	6	0	3.406541	-1.302150	1.171547
26	6	0	4.386537	-1.070632	0.205773
27	1	0	4.871352	0.036938	-1.576873
28	1	0	3.601241	-2.012200	1.972309
29	6	0	1.131573	-0.906961	2.195436
30	1	0	0.674307	0.021752	2.551824
31	1	0	0.328785	-1.538238	1.796905
32	1	0	1.575260	-1.423787	3.049288
33	6	0	2.616452	1.503350	-1.997691
34	1	0	1.663442	1.269743	-2.484052
35	1	0	2.547980	2.528604	-1.620383
36	1	0	3.406949	1.465337	-2.750378
37	6	0	5.712641	-1.785428	0.253495
38	1	0	5.910295	-2.304427	-0.689755
39	1	0	6.531664	-1.076463	0.414248
40	1	0	5.735985	-2.521201	1.061036
41	6	0	-1.371804	3.870674	0.579126
42	1	0	-1.558490	4.028407	1.646331
43	1	0	-0.883334	4.757547	0.175064
44	6	0	-2.666322	3.482048	-0.175715
45	1	0	-2.574067	3.775167	-1.224887
46	1	0	-3.551702	3.962441	0.241972

Cartesian coordinates of TS3

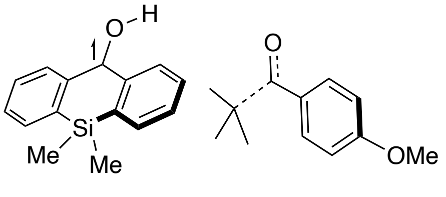

Center Number	Atomic Number	Atomic Type	Coordinates (Angstroms) X	Y	Z
1	6	0	12.959357	0.675645	-1.116248
2	6	0	12.220435	-0.433602	-0.696147

3	6	0	12.640665	-1.128788	0.432153
4	6	0	13.761385	-0.723638	1.150920
5	6	0	14.481752	0.394522	0.726516
6	6	0	14.078340	1.090664	-0.416886
7	1	0	12.656190	1.214771	-2.008805
8	1	0	12.081352	-2.006353	0.737105
9	1	0	14.065067	-1.285415	2.023819
10	8	0	15.587252	0.874083	1.350341
11	6	0	16.042446	0.194226	2.501746
12	1	0	16.925389	0.731844	2.839320
13	1	0	15.285461	0.204544	3.292013
14	1	0	16.312698	-0.840229	2.268710
15	6	0	11.018300	-0.893775	-1.459999
16	1	0	10.889602	-0.386981	-2.435628
17	6	0	9.484099	0.163049	-0.705819
18	6	0	9.865924	1.609614	-0.871683
19	1	0	10.720050	1.882567	-0.250914
20	1	0	10.084487	1.857209	-1.913640
21	1	0	9.012893	2.227505	-0.561323
22	6	0	9.384481	-0.302220	0.720816
23	1	0	9.256842	-1.384568	0.769564
24	1	0	10.264798	-0.016478	1.299632
25	1	0	8.509501	0.170447	1.185364
26	8	0	10.584535	-2.063263	-1.320557
27	6	0	8.352753	-0.286929	-1.592876
28	1	0	7.433769	0.225609	-1.281909
29	1	0	8.536521	-0.031325	-2.639267
30	1	0	8.197566	-1.362368	-1.516480
31	1	0	14.664729	1.944077	-0.734019
32	6	0	-11.142317	-1.107312	0.259967
33	6	0	-11.505848	0.263077	0.199904
34	6	0	-12.858243	0.603070	0.260111
35	6	0	-13.854822	-0.359336	0.380194
36	6	0	-13.492495	-1.703991	0.453995
37	6	0	-12.161928	-2.076674	0.399282
38	6	0	-9.772680	-1.557178	0.178013
39	6	0	-8.602219	-0.774869	-0.135451
40	6	0	-8.622488	0.647504	-0.166117
41	6	0	-7.381173	-1.429066	-0.432404
42	6	0	-6.226164	-0.715351	-0.699587
43	6	0	-6.241108	0.677182	-0.696342

44	6	0	-7.441609	1.334461	-0.442783
45	8	0	-9.628180	-2.898205	0.354594
46	1	0	-13.144826	1.650710	0.216978
47	1	0	-14.897406	-0.067995	0.422017
48	1	0	-14.256103	-2.466676	0.554199
49	1	0	-11.891153	-3.121982	0.455338
50	1	0	-7.329592	-2.509523	-0.512116
51	1	0	-5.311356	-1.249552	-0.928360
52	1	0	-5.338272	1.238019	-0.904290
53	1	0	-7.454952	2.421211	-0.463799
54	14	0	-10.203136	1.597964	0.144015
55	6	0	-10.121494	2.516620	1.779868
56	1	0	-9.318910	3.259323	1.770374
57	1	0	-11.061778	3.038285	1.978558
58	1	0	-9.936232	1.823832	2.603261
59	6	0	-10.525946	2.826278	-1.238014
60	1	0	-9.739370	3.584603	-1.280029
61	1	0	-10.567480	2.322204	-2.205399
62	1	0	-11.475179	3.346217	-1.082325
63	1	0	-8.715678	-3.105047	0.572043

Cartesian coordinates of *t*Bu radical

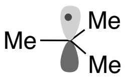

Center Number	Atomic Number	Atomic Type	Coordinates (Angstroms) X	Y	Z
1	6	0	-0.000018	0.000014	-0.183509
2	6	0	1.200223	0.865806	0.019138
3	1	0	1.058984	1.857184	-0.419091
4	1	0	1.410367	1.017241	1.090553
5	1	0	2.096363	0.419133	-0.419306
6	6	0	0.149709	-1.472298	0.019140
7	1	0	-0.685659	-2.025018	-0.418416
8	1	0	1.078416	-1.845812	-0.419974
9	1	0	0.176894	-1.729893	1.090553

10	6	0	-1.349937	0.606495	0.019140
11	1	0	-1.586445	0.711995	1.090577
12	1	0	-1.410995	1.606217	-0.418609
13	1	0	-2.137790	-0.011151	-0.419746

Cartesian coordinates of anisaldehyde

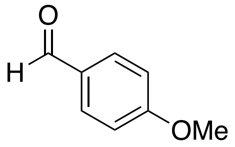

Center Number	Atomic Number	Atomic Type	Coordinates (Angstroms) X	Y	Z
1	6	0	-0.803116	1.413869	-0.000259
2	6	0	-1.470549	0.185372	-0.000201
3	6	0	-0.730343	-0.994727	0.000239
4	6	0	0.656474	-0.962080	0.000657
5	6	0	1.313610	0.274049	0.000619
6	6	0	0.577121	1.464122	0.000243
7	1	0	-1.377107	2.335333	-0.000713
8	1	0	-1.260624	-1.940267	0.000384
9	1	0	1.213101	-1.889069	0.001177
10	8	0	2.656648	0.417133	0.000877
11	6	0	3.453910	-0.751736	-0.001182
12	1	0	4.485743	-0.409490	-0.002715
13	1	0	3.270383	-1.354380	0.892978
14	1	0	3.267323	-1.353303	-0.895419
15	6	0	-2.946569	0.145582	-0.000560
16	1	0	1.116516	2.402751	0.000266
17	8	0	-3.604107	-0.864296	0.000149
18	1	0	-3.438892	1.139023	-0.001503

· **Calculated BDFEs**

The BEFE was calculated as follows: BDFE (A–H) = G°(A$^\bullet$) + G°(H$^\bullet$) – G°(A–H)

Where:

G°(A$^\bullet$) is the Gibbs free energy of the radical A$^\bullet$

G°(H$^\bullet$) is the Gibbs free energy of the hydrogen radical

G°(A–H) is the Gibbs free energy of the A–H molecule

substrate	X radical	Free Energy of substrate (Hartree)	Free Energy of X radical (Hartree)	Free Energy of H radical (Hartree)	calculated BDFE (kcal/mol)
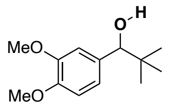	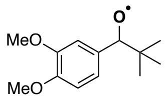	-618.21901	-617.55805	-0.50885	95.5
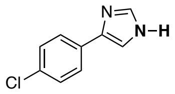	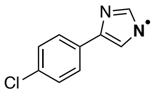	-916.69869	-916.05973	-0.50885	81.6
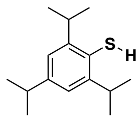	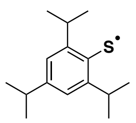	-983.80913	-983.19273	-0.50885	67.5
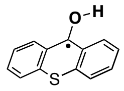	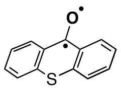	-973.95796	-973.29152	-0.50885	98.9
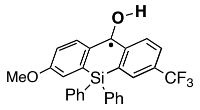	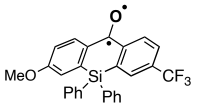	-1779.8772	-1779.2157	-0.50885	95.8

■ Supplementary References ■

(1) Campbell, M. W.; Yuan, M.; Polites, V. C.; Gutierrez, O.; Molander, G. A. Photochemical C–H Activation Enables Nickel-Catalyzed Olefin Dicarbofunctionalization. *J. Am Chem. Soc.* **2021**, *143*, 3901–3910. https://doi.org/10.1021/jacs.0c13077.

(2) Erchinger, J. E.; Hoogesteger, R.; Laskar, R.; Dutta, S.; Hümpel, C.; Rana, D.; Daniliuc, C. G.; Glorius, F. EnT-Mediated N–S Bond Homolysis of a Bifunctional Reagent Leading to Aliphatic Sulfonyl Fluorides. *J. Am. Chem. Soc.* **2023**, *145*, 2364–2374. https://doi.org/10.1021/jacs.2c11295.

(3) Zhao, K.; Wang, H.; Li, T.; Liu, S.; Benassi, E.; Li, X.; Yao, Y.; Wang, X.; Cui, X.; Shi, F. Identification of a Potent Palladium-Aryldiphosphine Catalytic System for High-Performance Carbonylation of Alkenes. *Nat. Commun.* **2024**, *15*. https://doi.org/10.1038/s41467-024-46286-9.

(4) Hertz, V. M.; Ando, N.; Hirai, M.; Bolte, M.; Lerner, H.-W.; Yamaguchi, S.; Wagner, M. Steric Shielding vs Structural Constraint in a Boron-Containing Polycyclic Aromatic Hydrocarbon. *Organomet.* **2016**, *36*, 2512–2519. https://doi.org/10.1021/acs.organomet.6b00800.

(5) Bomon, J.; Van Den Broeck, E.; Bal, M.; Liao, Y.; Sergeyev, S.; Van Speybroeck, V.; Sels, B. F.; Maes, B. U. W. Brønsted Acid Catalyzed Tandem Defunctionalization of Biorenewable Ferulic Acid and Derivates into Bio-Catechol. *Angew. Chem. Int. Ed.* **2020**, *59*, 3063–3068. https://doi.org/10.1002/anie.201913023.

(6) Liang, J.; Wang, M.; Zhao, Y.; Yan, W.; Si, X.; Yu, G.; Cao, J.; Wei, X. Nano WO3-Catalyzed One-Pot Process for Mild Oxidative Depolymerization of Lignin and Its Model Compounds. *ChemCatChem* **2021**, *13*, 3836–3845. https://doi.org/10.1002/cctc.202100670.

(7) Wu, H.; Li, X.; Yang, L.; Chen, W.; Zou, C.; Deng, W.; Wang, Z.; Hu, J.; Li, Y.; Huang, Y. Cathodic Carbonyl Alkylation of Aryl Ketones or Aldehydes with Unactivated Alkyl Halides. *Org. Lett.* **2022**, *24*, 9342–9347. https://doi.org/10.1021/acs.orglett.2c04019.

(8) Reddel, J. C. T.; Wang, W.; Koukounas, K.; Thomson, R. J. Triflimide-Catalyzed Allylsilane Annulations of Benzylic Alcohols for the Divergent Synthesis of Indanes and Tetralins. *Chem. Sci.* **2017**, *8*, 2156–2160. https://doi.org/10.1039/c6sc04762a.

(9) Suh, S. M.; Jambu, S.; Chin, M. T.; Diao, T. Selective Cleavage of Lignin Model Compounds via a Reverse Biosynthesis Mechanism. *Org. Lett.* **2023**, *25*, 4792–4796. https://doi.org/10.1021/acs.orglett.3c01416.

(10) Liu, C.; Han, J.; Marcelina, O.; Nugrahaningrum, D. A.; Huang, S.; Zou, M.; Wang, G.; Miyagishi, M.; He, Y.; Wu, S.; Kasim, V. Discovery of Salidroside-Derivated Glycoside Analogues as Novel Angiogenesis Agents to Treat Diabetic Hind Limb Ischemia. *J. Med. Chem.* **2021**, *65*, 135–162. https://doi.org/10.1021/acs.jmedchem.1c00947.

(11) Antico, E.; Schlichter, P.; Werlé, C.; Leitner, W. Reduction of Carboxylic Acids to Alcohols via Manganese(I) Catalyzed Hydrosilylation. *JACS Au* **2021**, *1*, 742–749. https://doi.org/10.1021/jacsau.1c00140.

(12) Drymona, M.; Kaplanai, E.; Vougioukalakis, G. C. An In-Situ-Formed Copper-Based Perfluorinated Catalytic System for the Aerobic Oxidation of Alcohols. *Eur. J. Org. Chem.* **2024**, *27*. https://doi.org/10.1002/ejoc.202301179.

(13) Benischke, A. D.; Anthore-Dalion, L.; Berionni, G.; Knochel, P. Preparation of Functionalized Diaryl- and Diheteroaryllanthanum Reagents by Fast Halogen–Lanthanum Exchange. *Angew. Chem. Int. Ed.* **2017**, *56*, 16390–16394. https://doi.org/10.1002/anie.201709553.

(14) Mandal, A.; Ganguli, K.; Pradhan, M.; Gorai, A.; Kundu, S. Selective Transfer Hydrogenation of C=O and Conjugated C=C Bonds Using An NHC-Based Pincer (CNC)MnI Complex in Methanol**. *ChemSusChem* **2023**, *16*. https://doi.org/10.1002/cssc.202300683.

(15) Pieber, B.; Malik, J. A.; Cavedon, C.; Gisbertz, S.; Savateev, A.; Cruz, D.; Heil, T.; Zhang, G.; Seeberger, P. H. Semi-heterogeneous Dual Nickel/Photocatalysis Using Carbon Nitrides: Esterification of Carboxylic Acids with Aryl Halides. *Angew. Chem. Int. Ed.* **2019**, *58*, 9575–9580. https://doi.org/10.1002/anie.201902785.

(16) Wang, L.; Yu, J.; Duan, Z.; Jin, J.; Zhang, Y. Cobalt-Catalyzed Synthesis of Aryl Ketones and Aldehydes from Redox-Active Esters. *Org. Biomol. Chem*. **2022**, *20*, 6554–6557. https://doi.org/10.1039/d2ob01275h.

(17) Borthakur, I.; Joshi, A.; Kumari, S.; Kundu, S. Metal-Free Visible-Light Induced Oxidative Cleavage of C(Sp^3)−C, and C(Sp^3)−N Bonds of Nitriles, Alcohols, and Amines. *Chem. Eur. J.* **2024**, *30*. https://doi.org/10.1002/chem.202303295.

(18) Dilauro, G.; Azzollini, C. S.; Vitale, P.; Salomone, A.; Perna, F. M.; Capriati, V. Scalable Negishi Coupling between Organozinc Compounds and (Hetero)Aryl Bromides under Aerobic Conditions When Using Bulk Water or Deep Eutectic Solvents with No Additional Ligands. *Angew. Chem. Int. Ed.* **2021**, *60*, 10632–10636. https://doi.org/10.1002/anie.202101571.

(19) Davenport, E.; Negru, D. E.; Badman, G.; Lindsay, D. M.; Kerr, W. J. Robust and General Late-Stage Methylation of Aryl Chlorides: Application to Isotopic Labeling of Drug-like Scaffolds. *ACS Catal.* **2023**, *13*, 11541–11547. https://doi.org/10.1021/acscatal.3c02761.

(20) Brandhofer, T.; Mancheño, O. G. Versatile Ru-Photoredox-Catalyzed Functionalization of Dehydro-Amino Acids and Peptides. *ChemCatChem* **2019**, *11*, 3797–3801. https://doi.org/10.1002/cctc.201900446.

(21) Kemmochi, M.; Miyamoto, Y.; Sumida, Y.; Ohmiya, H. Direct Photoexcitation of Borate Enabling Minisci Reaction. Asian J. Org. Chem. **2021**, *11*. https://doi.org/10.1002/ajoc.202100640.

(22) Gaussian 16, Revision C.01, Frisch, M. J.; Trucks, G. W.; Schlegel, H. B.; Scuseria, G. E.; Robb, M. A.; Cheeseman, J. R.; Scalmani, G.; Barone, V.; Petersson, G. A.; Nakatsuji, H.; Li, X.; Caricato, M.; Marenich, A. V.; Bloino, J.; Janesko, B. G.; Gomperts, R.; Mennucci, B.; Hratchian, H. P.; Ortiz, J. V.; Izmaylov, A. F.; Sonnenberg, J. L.; Williams-Young, D.; Ding, F.; Lipparini, F.; Egidi, F.; Goings, J.; Peng, B.; Petrone, A.; Henderson, T.; Ranasinghe, D.; Zakrzewski, V. G.; Gao, J.; Rega, N.; Zheng, G.; Liang, W.; Hada, M.; Ehara, M.; Toyota, K.; Fukuda, R.; Hasegawa, J.; Ishida, M.; Nakajima, T.; Honda, Y.; Kitao, O.; Nakai, H.; Vreven, T.; Throssell, K.; Montgomery, J. A., Jr.; Peralta, J. E.; Ogliaro, F.; Bearpark, M.; Heyd, J. J.; Brothers, E. N.; Kudin, K. N.; Staroverov, V. N.; Keith, T. A.; Kobayashi, R.; Normand, J.; Raghavachari, K.; Rendell, A. P.; Burant, J. C.; Iyengar, S. S.; Tomasi, J.; Cossi, M.; Millam, J. M.; Klene, M.; Adamo, C.; Cammi, R.; Ochterski, J. W.; Martin, R. L.; Morokuma, K.; Farkas, Ö.; Foresman, J. B.; Fox, D. J. Gaussian, Inc., Wallingford, CT, 2016.

(23) Zhao, Y. & Truhlar, D. G. The M06 suite of density functionals for main group thermochemistry, thermochemical kinetics, noncovalent interactions, excited states, and transition elements: two new functionals and systematic testing of four M06-class functionals and 12 other functionals. *Theor. Chem. Acc.* **2008**, *120*, 215–241
